# Multi‐Targeting Non‐Specific Genome Engineering in Bacteria

**DOI:** 10.1002/advs.202521532

**Published:** 2026-03-09

**Authors:** Runze Sun, Ruixiang You, Yiwen Zhou, Huiyan He, Wenfang Wang, Xudong Qu, Yinhua Lu, Lei Li

**Affiliations:** ^1^ State Key Laboratory of Microbial Metabolism and School of Life Sciences and Biotechnology Shanghai Jiao Tong University Shanghai China; ^2^ College of Life Sciences Shanghai Normal University Shanghai China

**Keywords:** bacteria, genome engineering, microbial drugs, multi‐targeting integrases, synthetic biology

## Abstract

Genome engineering plays a crucial role in the rapidly growing fields of metabolic engineering and synthetic biology. Chromosomal integration and stable expression of functional genes or large metabolic pathways necessitate the development of host‐independent enabling technologies in diverse bacteria. Here, a generalizable genome engineering approach, MNGE (Multi‐targeting Non‐specific Genome Engineering), is developed based on the multi‐targeting integrase (MTI) systems for multi‐copy (at least three copies), highly random (only requiring the core TT dinucleotide) integration of metabolic genes or pathways in both Gram‐positive bacteria (i.e., *Streptomyces* and *Saccharopolyspora*) and Gram‐negative bacteria (i.e., *Burkholderia* and *Chromobacterium*). Using MNGE, the fungicide UK‐2 BGC (41 kb) and the polyether antibiotic salinomycin BGC (106 kb) were randomly integrated into a heterologous host *Streptomyces albus*, significantly enhancing their fermentation levels based on chromosome position effects. Furthermore, the potent G_q/11_‐signaling inhibitor FR900359 BGC (66 kb) was successfully expressed in *Burkholderia gladioli* by the MTI1 system. Together, the MNGE approach exhibits broad applicability for next‐generation genome engineering in diverse bacteria, thereby achieving highly efficient production of high‐value compounds.

## Graphical abstract

In this study, we provide the first case to use the multi‐targeting integrase (MTI) systems in bacteria and develop a host‐independent generalizable approach, MNGE (Multi‐targeting Non‐specific Genome Engineering), for multi‐copy and random integration of metabolic genes or pathways in both Gram‐positive and Gram‐negative bacteria, which will significantly accelerate efficient production of high‐value compounds in metabolic engineering and synthetic biology.

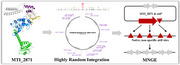

## 1 Introduction

Genome engineering plays a crucial role in metabolic engineering and synthetic biology for industrial production of biofuels, chemicals, pharmaceuticals, and other high‐value compounds [1, 2, 3]. Compared to plasmid‐based systems, chromosomal integration can overcome the population heterogeneity and has intrinsic genetic stability for reliable maintenance and expression of desired metabolic genes or pathways at reduced metabolic burden, especially in large‐scale and long‐term fermentation [4]. Different from homologous recombination (HR) or transposition‐mediated DNA integration, large serine recombinases (LSRs)‐driven site‐specific integration exhibits a naturally mechanistic advantage that there is no obvious upper limit on the size of the donor DNA, which is particularly suitable for genomic insertion of large metabolic pathways such as bacterial natural product biosynthetic gene clusters (BGCs, ∼10–150 kb) and nitrogen fixation BGCs (∼10–60 kb) [5, 6, 7, 8]. Actually, LSRs can integrate self‐located mobile genetic elements (MGEs, i.e., entire phage genomes and conjugative elements) into recipient chromosomes in larger DNA sizes (20 to >500 kb) without relying on recipient genetic repair machinery or requiring any cellular cofactors [9]. These features make LSRs highly attractive genome engineering tools for the construction of microbial cell factories [10, 11]. However, the practical application of LSRs has been limited in the rapidly growing fields of metabolic engineering and synthetic biology by several factors, including low integration efficiency, limited numbers of identified LSRs (i.e., Bxb1 and PhiC31) and most notably sequence‐defined landing sites (∼30–50 nucleotides long) that do not exist in most of bacteria and require pre‐installation of their preferred attachment sites [5, 6]. To overcome these limitations, it is necessary and urgent to develop versatile, host‐independent genome engineering tools for chromosome‐based stable expression of functional genes or large metabolic pathways in diverse bacteria [12, 13, 14].

**FIGURE 1:**
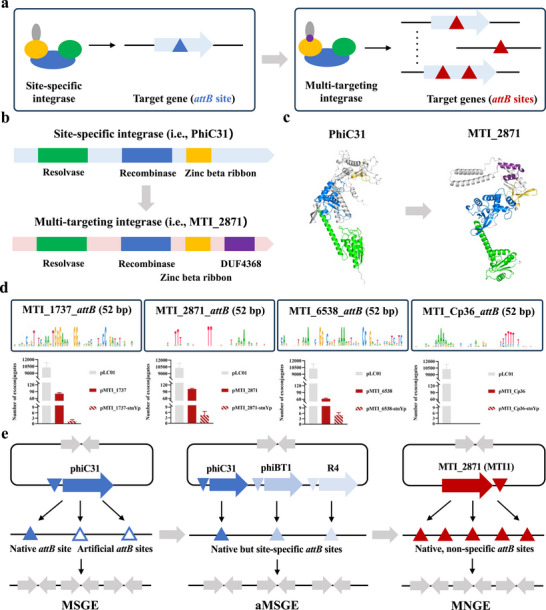
Design and construction of MNGE based on multi‐targeting integrases (MTIs). (a) Comparison in target genes (*attB* sites) between site‐specific integrases and multi‐targeting integrases. (b) Comparison in domain architectures between site‐specific integrases (i.e, PhiC31) and multi‐targeting integrases (i.e., MTI_2871). (c) Comparison in AlphaFold2‐predicted protein structures between the site‐specific integrase PhiC31 and the multi‐targeting integrase MTI_2871. (d) Native or previously identified *attB* sequences as well as integration efficiency of four MTIs (MTI_1737, MTI_2871, MTI_6538, and MTI_Cp36) in the heterologous host *S. albus* J1074. (e) Schematics of the MSGE, aMSGE, and MNGE approaches.

Usually, LSRs can recombine two distinct but highly conserved DNA attachment sites found on an invading MGE (*attP*) and in the target prokaryotic genome (*attB*) [15, 16]. Intriguingly, a few LSRs have evolved multi‐targeting or transposition capabilities, allowing them to target variable *attB* sites in a given bacterial genome [17]. Efficient insertion of metabolic genes or pathways into a defined series of native attachment sites would be very useful because of no requirement of time‐consuming and usually low‐efficiency pre‐installation of genomic landing pads. Theoretically, compared to canonical site‐specific LSRs such as PhiC31, multi‐targeting integrases (MTIs) have more relaxed sequence specificity and could target sequences that occurred at multiple different genomic sites (Figure 1a). Recently, a systematic survey of LSRs from sequencing bacterial (meta)genomic data has provided over 60 newly characterized LSRs in human cells for efficient integration of large DNA sequences (up to 7 kb) [18]. Notably, a unique MTI clade of the LSR family has been discovered, and three MTIs, including MTI_Cp36, MTI_Enc9, and MTI_Pc01, have been demonstrated to efficiently integrate DNA cargoes into multiple, non‐specific sites of the human genome. In the MTI clade, an unknown Pfam domain DUF4368 is found in 63% of identified LSRs (i.e., MTI_2871), but is rarely (0.73%) found in site‐specific integrases (i.e., PhiC31), which might be responsible for the multi‐targeting feature of MTIs (Figure 1b, c) [18]. The conserved DNA integration mechanism of LSRs raises the possibility that the MTI systems could be harnessed for next‐generation, host‐independent genome engineering (i.e., multi‐targeting, non‐specific chromosomal integration of large DNA cargoes) in diverse organisms. However, the MTI systems have yet to be demonstrated in other organisms besides human cells, including plants, fungi, and bacteria.

In this study, we sought to extend the MTI systems from human cells to bacteria for random chromosomal integration and stable expression of functional genes or large metabolic pathways. Taking advantage of these key features of MTIs, we developed a host‐independent generalizable MNGE approach (Multi‐targeting, Non‐specific Genome Engineering) in both Gram‐positive bacteria (i.e., *Streptomyces* and *Saccharopolyspora*) and Gram‐negative bacteria (i.e., *Burkholderia* and *Chromobacterium*) and demonstrated three key applications: (1) highly random integration of the reporter gene cassette *idgS‐sfp* with up to three copies in a single genome; (2) high‐efficient production of the next‐generation fungicide fenpicoxamid precursor UK‐2 and the anti‐tumor compound salinomycin in a heterologous *Streptomyces* host based on chromosome position effects; (3) heterologous expression of the potent G_q/11_‐signaling inhibitor FR900359 BGC (66 kb) in *Burkholderia gladioli*. Together, our study emphasizes the untapped potential of MTIs for developing the MNGE approach for next‐generation genome engineering in bacteria and overcoming the limitations of existing technologies.

## 2 Results

### 2.1 Design and Construction of MNGE

To develop MNGE for multi‐targeting, non‐specific integration of metabolic genes or pathways in bacteria, we first selected four MTIs for experimental verification, including MTI_1737, MTI_2871, MTI_6538, and MTI_Cp36 (Figure S1‐S4) [18]. As shown in the Figure 1d and Figure S5, MTI_1737 contains the conserved GG dinucleotide cores with 5′ and 3′ ends enriched for A/G and C nucleotides; MTI_2871 contains the conserved TT dinucleotide cores with 5′ and 3′ ends enriched for T and A nucleotides; MTI_6538 contains the conserved AA dinucleotide cores with 5′ and 3′ ends enriched for A/G and C/G nucleotides; MTI_Cp36 contains a non‐conserved dinucleotide core with 5′ and 3′ ends enriched for A and T nucleotides. The four MTIs target 17, 14, 21, and 33 distinct native or previously identified *attB* sites, respectively, which suggests that they most likely have highly relaxed sequence specificity [18]. With diverse and relaxed targeting sequences, the four MTI systems we selected here are expected to work in different bacterial systems.

With the four codon‐optimized MTI genes with native *attP* sites under the control of two strong promoters ermEp* and *stnYp*), we tested the four MTI systems in the model strain *Streptomyces albus* J1074 (Figure S6). As shown in Figure 1d, three integration plasmids with MTI_1737, MTI_2871, and MTI_6538 were successfully introduced into *S. albus* J1074, and the promoter ermEp*‐driven expression of MTI_1737, MTI_2871, and MTI_6538 was significantly better than that of the promoter *stnYp*. Interestingly, MTI_Cp36 could work well in human cells but could not introduce foreign plasmids into *S. albus* J1074 (Figure 1d) [18]. Although the integration efficiencies for MTI_1737, MTI_2871, and MTI_6538 were lower than that of PhiC31, we here demonstrated for the first time that multiple MTI systems could achieve genomic integration of foreign DNA cargoes in bacteria (i.e., *Streptomyces*).

In the three above MTIs that could work in *S. albus* J1074, the integration efficiency for MTI_2871 was the highest (Figure 1d). Meanwhile, its specificity for the native target *attB* sites was more relaxed than that of MTI_1737 or MTI_6538 (Figure 1d and Figure S5). Therefore, we designed the MNGE approach based on MTI_2871, which was renamed as MTI1. The novel approach is distinct from our previously developed MSGE (Multiplexed Site‐specific Genome Engineering) or aMSGE (advanced MSGE) methods for multi‐copy, site‐specific integration of metabolic genes or pathways (Figure 1e) [19, 20]. The MSGE method, based on the “one LSR‐multiple artificial *attB* sites” concept, requires multi‐round, time‐consuming pre‐installation of artificial *attB* sites (Figure 1e) [19]. In particular, it is difficult and even impossible to introduce foreign *attB* sites into the genomes of genetically intractable bacteria [4, 21]. The aMSGE method, based on the “multiple LSRs‐multiple native *attB* sites” concept, takes advantage of native *attB* sites of different compatible LSR systems in the actinomycetal genomes, which efficiently overcomes the limitations of MSGE (Figure 1e) [20]. However, the method could only be used in actinomycetes, especially *Streptomyces*, because there are no active *attB* sites for known LSRs in most bacteria [4, 22]. Considering the native *attB* sites of MTI1 are not conserved and widely distributed in a single genome, the MNGE method based on the “one MTI‐multiple native *attB* sites” concept is theoretically expected to enable multi‐targeting, non‐specific integration of metabolic genes or pathways in other bacteria besides actinomycetes, especially without pre‐installation of artificial *attB* sites (Figure 1e). Together, compared to the MSGE and aMSGE methods, we expect that the MNGE method would be widely used in different bacteria, which was further confirmed in our following study.

### 2.2 Implementing MNGE for Multi‐Targeting, Non‐Specific Integration of Functional Genes

**FIGURE 2:**
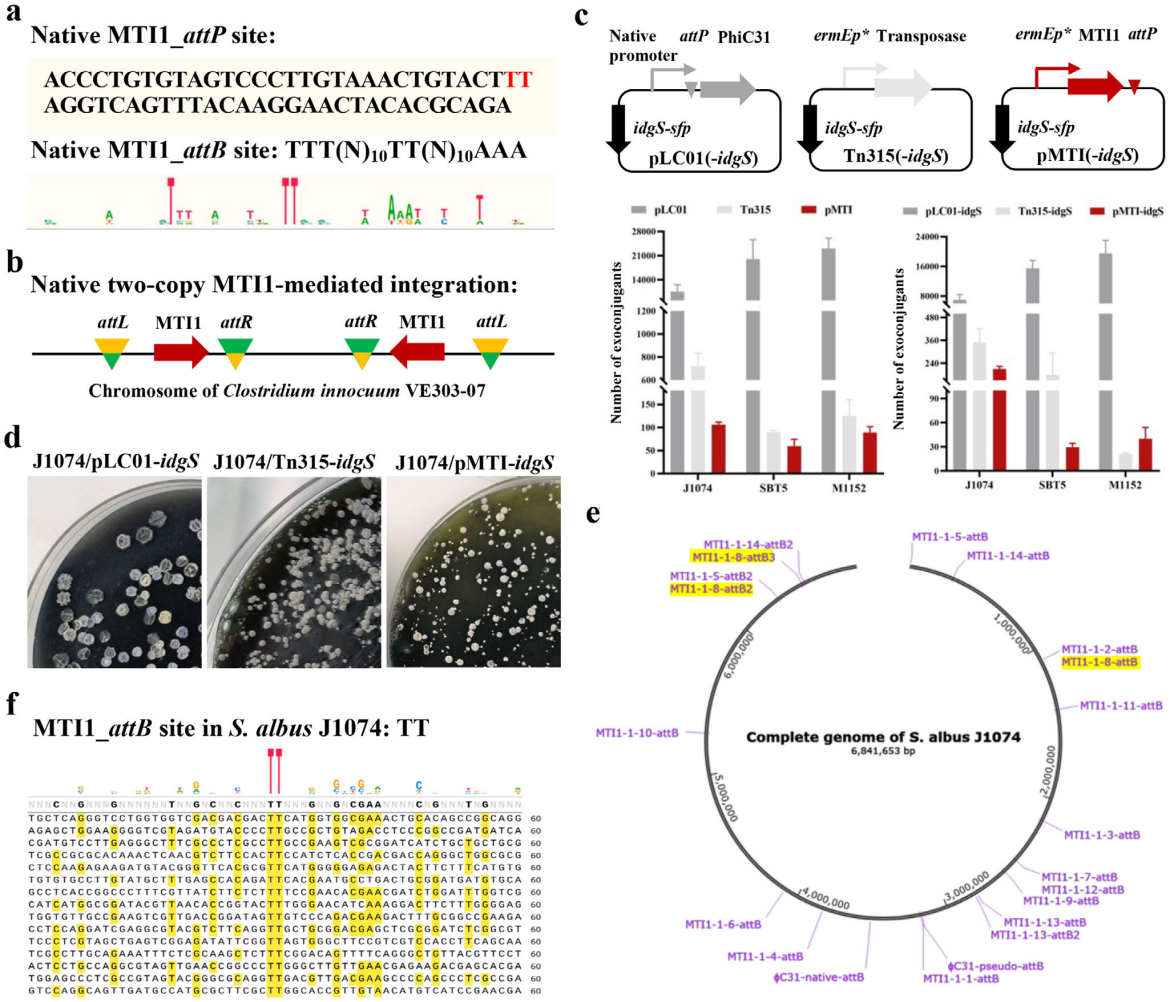
Implementing MNGE for multi‐targeting, non‐specific integration of functional genes (i.e., *idgS‐sfp*). (a) The native *attP* and *attB* sites of MTI1. (b) MTI1‐mediated two‐copy integration of MGEs in the native host *C. innocuum* VE303‐07. (c) MTI1‐mediated integration efficiency of *idgS‐sfp* in the three heterologous hosts, including *S. albus* J1074, *S. lividans* SBT5, and *S. coelicolor* M1152. pLC01‐*idgS* and Tn315‐*idgS* were used as the controls. (d) Growth phenotypes of exconjugants when MTI1 was used for integrating *idgS‐sfp* into *S. albus* J1074. pLC01‐*idgS* and Tn315‐*idgS* were used as the controls. (e) Genetic map of MTI1‐mediated integration of *idgS‐sfp* in *S. albus* J1074. A total of 14 independent, *idgS‐sfp*‐integrated exconjugants were sequenced. (f) Schematic of an alignment of diverse *attB* sequences that were targeted by MTI1 for the genomic integration of *idgS‐sfp*.

We bioinformatically analyzed whether the MTI1 system could mediate multi‐targeting insertion of MGEs in native hosts (Figure 2a). Intriguingly, we found two identical MGEs containing the MTI1 gene in the chromosome of *Clostridium innocuum* VE303‐07 (Figure 2b and Figure S7). Actually, we also found a similar phenomenon for MTI_1737, which also mediated the integration of two same MGEs in the genome of *Amedibacterium intestinale* JCM 30884 (Figure S7). However, only one copy of their corresponding MGEs in different bacterial genomes was found for MTI_6538 or MTI_Cp36 (Figure S7). These results indicated that the MTI systems are able to achieve at least two‐copy chromosomal integration of MGEs in native hosts. Therefore, we speculated that the MNGE approach could be used for multi‐targeting integration of foreign DNA constructs at least in *Amedibacterium* and *Clostridium*, such as the two important industrial microorganisms *Clostridium acetobutylicum* and *Clostridium ljungdahlii* [23, 24].

To check whether the MTI1 system could be used to mediate chromosomal integration of foreign genes, here the gene cassette *idgS*‐*sfp* (4.8 kb) encoding the blue pigment indigoidine was used as the reporter system, and *Streptomyces*, the main source of bioactive natural products, was used as the tested bacterial chassis [25]. The site‐specific PhiC31 integration plasmid pLC01‐*idgS* and the random transposon plasmid Tn315‐*idgS* were used as the controls (Figure S8). As shown in Figure 2c, the non‐specific integration plasmid pMTI‐*idgS* was efficiently integrated into three model *Streptomyces* hosts, including *S. albus* J1074, *S. lividans* SBT5, and *Streptomyces coelicolor* M1152. Notably, different from the identical growth phenotypes of the J1074/pLC01‐*idgS* exconjugants, we found that the J1074/pMTI‐*idgS* exconjugants exhibited obviously distinct colony sizes, which were similar to those of the J1074/Tn315‐*idgS* exconjugants (Figure 2d). To further increase the MTI1 integration efficiency of the reporter gene cassette *idgS*‐*sfp*, the stronger promoter *stnYp* was used for driving the expression of the MTI1 gene. Interestingly, pMTI‐*stnYp*‐*idgS* had more exconjugants than pMTI‐*idgS* after being introduced into J1074; almost all exconjugants exhibited small‐colony features, which indicated that the overexpression of MTI1 might result in an obvious toxicity to J1074 (Figure S9). Together, these results indicated that the MTI1 system efficiently achieved the chromosomal integration of the reporter gene cassette in three tested model *Streptomyces* strains, potentially in a non‐specific manner.

We then randomly selected 14 exconjugants of J1074/pMTI‐*idgS* for identifying the integration sites of *idgS*‐*sfp* by whole‐genome sequencing (Figure S10). As shown in Figure 2e and Figure S11, we found that the *idgS*‐*sfp* cassette was indeed randomly inserted into the genome of J1074 with up to three‐copy integration in a single exconjugant. Notably, the MTI1 system had more relaxed *attB* sequence specificity in J1074 than that in native hosts, which only contains the conserved TT dinucleotide cores (Figure 2a and f). The phenomenon might be due to the high GC content feature in the genome of *Streptomyces*, which resulted in the 5′ and 3′ ends of the MTI1's *attB* sequences not being enriched for T and A nucleotides. More importantly, considering that the TT dinucleotide is distributed in the genomes of all organisms, the MNGE method based on the MTI1 system is expected to be widely used in bacteria. To explore whether the multi‐copy integration of *idgS*‐*sfp* could enhance indigoidine titers, we cultured 140 exconjugants and then selected 14 highly efficient indigoidine‐producing strains for identifying integrated sites of *idgS‐sfp* (Figure S12). Intriguingly, we found that out of 14 strains, six had two‐copy integration of *idgS‐sfp*, and the others had only one‐copy integration of *idgS‐sfp* with random genomic distribution (Figure S12b). However, the average indigoidine titer of the six two‐copy strains was slightly higher than that of the eight one‐copy strains (Figure S13). We speculated that the precursor L‐glutamine may not be enough for higher production of indigoidine in the two‐copy strains. Indeed, after feeding 5 mM L‐glutamine, the indigoidine titers of the four two‐copy strains, including J1074/pMTI‐*idgS*‐2‐11, 2–29, 2–61, and 2–126, were significantly enhanced (Figure S13d). The indigoidine titer of the two‐copy engineered strain J1074/pMTI‐*idgS*‐2‐29 reached up to the highest level with 457.9 mg/L after feeding 5 mM L‐glutamine, 15.7% and 36.3% higher than those of the one‐copy strain J1074/pMTI‐idgS‐2‐110 and the two‐copy strain J1074/pLC1‐*idgS*, respectively (Figure S13d).

Collectively, the results clearly demonstrated that the MTI1 system exhibited more relaxed *attB* sequence specificity and could directly achieve the random integration of foreign genes with up to three copies in *Streptomyces*. Both the copy number effects due to the multi‐targeting integration feature and the chromosome position effects due to the non‐specific integration feature of the MTI1 system could efficiently increase the expression levels of functional genes, such as the reporter gene cassette *idgS*‐*sfp*.

### 2.3 Implementing MNGE for Non‐Specific Integration of Large‐Size Natural Product BGCs in *Streptomyces*

**FIGURE 3:**
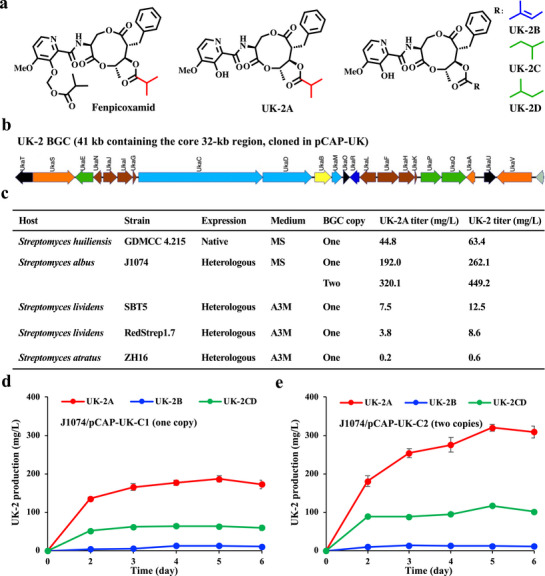
Native and heterologous expression of the UK‐2 BGC. (a) The chemical structures of fenpicoxamid, UK‐2A, UK‐2B, UK‐2C and UK‐2D. (b) The UK‐2 biosynthetic gene cluster (BGC) from *S. huiliensis* GDMCC 4.215. (c) UK‐2A and UK‐2 production titers when the UK‐2 BGC was expressed in native or four different heterologous hosts. Fermentation samples for HPLC analysis were collected on the fifth day. (d) Time‐course UK‐2 production titers in J1074/pCAP‐UK‐C1 (one‐copy integration of the UK‐2 BGC). (e) Time‐course UK‐2 production titers in J1074/pCAP‐UK‐C2 (two‐copy integration of the UK‐2 BGC).

The emergence and widespread distribution of multidrug‐resistant bacterial or fungal pathogens represent a growing and serious risk to global public health and food security, which necessitates the development of antibiotics with novel modes of action [26, 27, 28]. Here we extended the MNGE method for engineering large metabolic pathways (i.e., natural product BGCs) for yield optimization of two important antibiotics, including the fungicide UK‐2A and the polyether antibiotic salinomycin [29, 30]. As the prodrug of the antifungal natural product UK‐2A, fenpicoxamid (Inatreq, Figure 3a) is a member of a novel picolinamide class of fungicides by targeting the Qi quinone binding site of cytochrome b and displays no cross‐resistance to other anti‐fungal agents [29, 31]. However, until now, only one patent from Dow AgroSciences was reported for UK‐2A yield optimization due to the protection of the parental strain *Streptoverticillium* sp. 3–7, in which the production titers of UK‐2A and UK‐2 (including UK‐2A and three analogs UK‐2B, C, D, Figure 3a) were increased up to 466.5 and 564.5 mg/L by introducing an extra copy of UK‐2 BGC, respectively [32]. By bioinformatics and fermentation analysis, we identified a novel UK‐2‐producing strain, *Streptomyces huiliensis* GDMCC 4.215, with UK‐2A and UK‐2 production titers of 44.8 and 63.4 mg/L in the MS medium, respectively (Figure 3b,c, and Figures S14, S15) [33]. After whole‐genome sequencing of *S. huiliensis* GDMCC 4.215 using the third‐generation high‐throughput sequencing technology, the complete BGC was confirmed for the biosynthesis of UK‐2 (Figure 3b and Figure S16, S17) [34]. Unfortunately, we found that the strain *S. huiliensis* GDMCC 4.215 was very difficult for genetic manipulation, such as the introduction of integrative plasmids (i.e., pLC01) or replicating plasmids (i.e., pKC1139), potentially due to the endogenous complex restriction‐modification systems and obvious resistance to some antibiotics (i.e., apramycin and kanamycin) (data not shown). Therefore, we tried to heterologously express the UK‐2 BGC in different *Streptomyces* hosts and then optimize the fermentation level of UK‐2 using the MNGE approach.

Using the CRISPR/Cas9‐assisted TAR cloning technology [35], we successfully captured the entire UK‐2 BGC (41 kb with the core 32‐kb region) into the cloning vector pCAP01 with the site‐specific PhiC31 system, thus generating the plasmid pCAP‐UK with further verification by restriction analysis (Figure S18a). Then, four model *Streptomyces* hosts, including *S. albus* J1074, *S. lividans* SBT5, and RedStrep1.7, as well as *Streptomyces atratus* ZH16, were used for heterologous expression of the UK‐2 BGC [36, 37]. All four hosts with the UK‐2 BGC successfully produced UK‐2 when using five different fermentation media (A3M, MS, R5a, GYM, and ISP2), and the UK‐2 titer in J1074/pCAP‐UK was highest in the medium MS (Figure 3c and Figure S19). Because there is one real and one pseudo PhiC31‐*attB* site in the genome of J1074 [38], two types of exconjugants for J1074/pCAP‐UK were obtained. When one copy of the UK‐2 BGC was integrated, the UK‐2 titer of the engineered strain J1074/pCAP‐UK‐C1 reached up to 262.1 mg/L (Figure 3c, d). When two copies of the UK‐2 BGC were integrated, the UK‐2 titer of the engineered strain J1074/pCAP‐UK‐C2 reached up to 449.2 mg/L, seven‐fold higher than that of the native producer (Figure 3c, e). Together, these results indicated that the genetically tractable *S. albus* J1074 was an excellent host for efficient production of UK‐2, and increasing BGC copy number could further optimize the biosynthesis of UK‐2.

**FIGURE 4:**
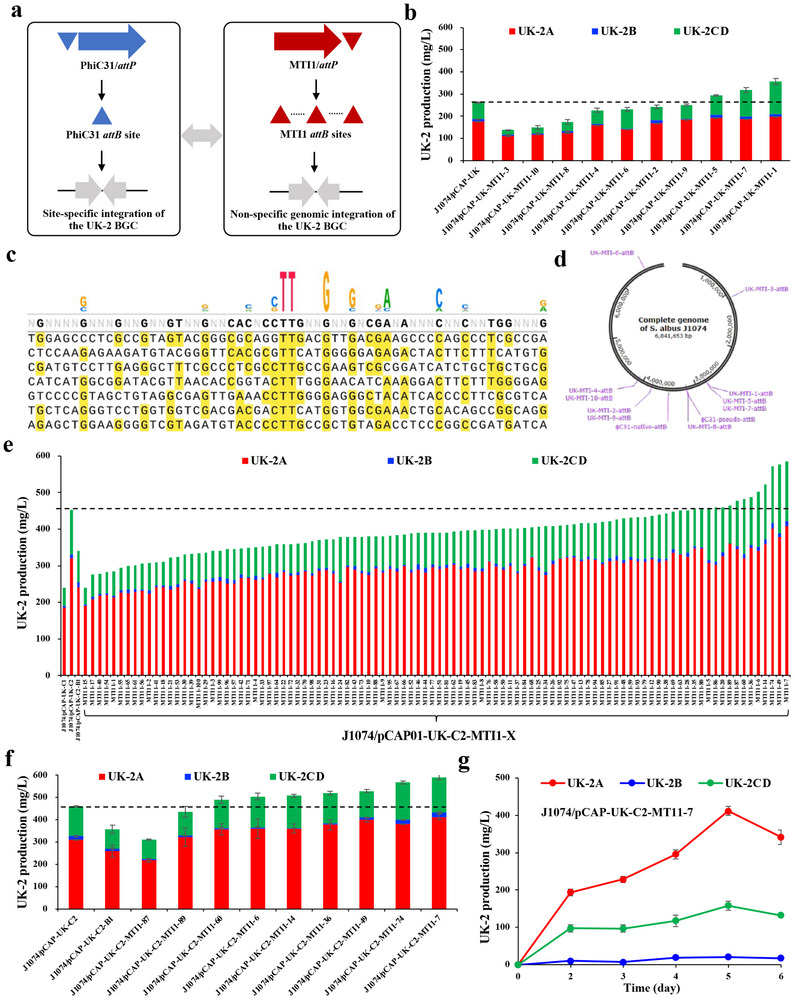
Harnessing MNGE for improving UK‐2 titers in *S. albus* J1074. (a) Schematic of MTI1‐mediated non‐specific genomic integration of the UK‐2 BGC based on *S. albus* J1074. PhiC31‐mediated site‐specific integration of the UK‐2 BGC was used as the control. (b) Fermentation results of 10 MTI1‐mediated exconjugants that produced UK‐2. (c) Schematic of an alignment of diverse *attB* sequences that were targeted by MTI1 for genomic integration of the UK‐2 BGC. (d) Genetic map of MTI1‐mediated integration sites of the UK‐2 BGC in *S. albus* J1074. (e) Screening of exconjugants when introducing pCAP‐UK‐MTI1 into J1074/pCAP‐UK‐C2 for high‐efficiently producing UK‐2. (f) Confirmation of nine engineered strains of J1074/pCAP‐UK‐C2‐MTI1 that efficiently produced UK‐2. (g) Time‐course UK‐2 titers of the best engineered strain J1074/pCAP‐UK‐C2‐MTI1‐7.

Using the iCASRED scarless DNA editing method [39], we first replaced the PhiC31 system in the plasmid pCAP‐UK with the MTI1 system, generating the edited plasmid pCAP‐UK‐MTI1 with verification by restriction analysis (Figure S18b). To test whether the MTI1 system could achieve random genomic integration of large natural product BGCs in *Streptomyces*,

We first introduced pCAP‐UK‐MTI1 into *S. albus* J1074. As shown in Figure 4b, out of 10 exconjugants of J1074/pCAP‐UK‐MTI1, three engineered strains exhibited higher UK‐2 titers than J1074/pCAP‐UK‐C1. The highest UK‐2 titer from the strain J1074/pCAP‐UK‐MTI1‐1 reached up to 357.1 mg/L, with 36.9% higher than that of J1074/pCAP‐UK‐C1 (Figure 4b). By complete genome sequencing, we found that there was only one copy of the UK‐2 BGC, and the integrated sites of the UK‐2 BGC introduced by the MTI1 system in the ten engineered strains showed highly random chromosomal distributions (Figure 4c, d). Our results preliminarily indicated that the MNGE approach enabled highly efficient production of UK‐2 in a heterologous *Streptomyces* host based on chromosome position effects [40].

Then, we tried to increase the copy numbers of the UK‐2 BGC using the MTI1 system based on J1074/pCAP‐UK‐C2 with another site‐specific PhiBT1 system as the control. Using the iCASRED scarless DNA editing method [39], the edited plasmid pCAP‐UK‐BT1 was constructed (Figure S18c). When introducing pCAP‐UK‐BT1 into J1074/pCAP‐UK‐C2, we found that the UK‐2 titer of the three‐copy strain J1074/pCAP‐UK‐C2‐B1 was lower than that of the starting strain J1074/pCAP‐UK‐C2, indicating that the genomic integration of the UK‐2 BGC up to three copies in a site‐specific manner might lead to metabolic burden of the three‐copy strain (Figure 4e, f). Therefore, we increased the number of exconjugants up to 100 for screening highly efficient UK‐2‐producing strains when introducing pCAP‐UK‐MTI1 into J1074/pCAP‐UK‐C2. Out of 100 exconjugants of J1074/pCAP01‐UK‐C2‐MTI1, UK‐2 titers of nine engineered strains were higher than those of the parental strain J1074/pCAP‐UK‐C2 (Figure 4e). Repeated fermentation results showed that seven of the nine J1074/pCAP‐UK‐C2‐MTI1 strains produced higher UK‐2 titers than those of J1074/pCAP‐UK‐C2 (Figure 4f). The highest UK‐2 titer from the strain J1074/pCAP‐UK‐C2‐MTI1‐7 reached up to 590.3 mg/L, 31.4% higher than that of J1074/pCAP‐UK‐C2 (Figure 4g). By complete genome sequencing, there were three copies of the UK‐2 BGC in the engineered strain J1074/pCAP‐UK‐C2‐MTI1‐7, as well as the other six UK‐2 high‐yield engineered strains. Unfortunately, the third copy of the UK‐2 BGC was introduced into the chromosome adjacent to the original UK‐2 BGC by a single crossover, not MTI1‐mediated random integration. The difference in UK‐2 titers in these engineered strains might be due to homologous recombination of different regions between the endogenous and exogenous UK‐2 BGCs. Considering that the integration efficiency of the MTI1 system was far lower than that of the PhiC31 system in *S. albus* J1074 (Figures 1d and 2c), we reasoned that homologous recombination, rather than MTI1‐mediated non‐specific integration, would play a dominant role when introducing a natural product BGC into bacteria already containing this BGC (i.e., a natural producer or a heterologous host with this BGC).

**FIGURE 5:**
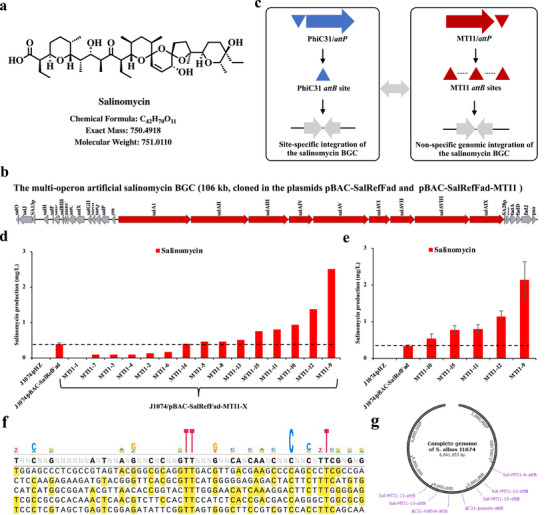
MTI1‐mediated random integration of an extra copy of the salinomycin BGC based on *S. albus* J1074. (a) Chemical structure of salinomycin; (b) The 106‐kb multi‐operon artificial salinomycin biosynthetic gene cluster (BGC); (c) Schematic of MTI1‐mediated non‐specific integration of the salinomycin BGC based on *S. albus* J1074. PhiC31‐mediated site‐specific integration of the salinomycin BGC was used as the control. (d) Screening of exconjugants derived from MTI1‐mediated genomic integration of the salinomycin BGC for highly efficient production of salinomycin. (e) Fermentation confirmation of the MTI1‐mediated exconjugants that efficiently produced salinomycin. (f) Schematic of an alignment of diverse *attB* sequences that were targeted by MTI1 for the genomic integration of the salinomycin BGC in the five salinomycin high‐yield engineered strains. (g) Genetic map of MTI1‐mediated integration of the salinomycin BGC in the five salinomycin high‐yield engineered strains.

The polyether antibiotic salinomycin is also a promising anticancer drug with selective activity against cancer stem cells (Figure 5a) [41]. Previously, the 106‐kb multi‐operon artificial salinomycin BGC was heterologously expressed in three different *Streptomyces* hosts (Figure 5b) [42]. Here, the salinomycin BGC was used to test whether the MTI1 system could achieve random chromosomal integration of larger‐sized natural product BGCs (>100 kb) in *S. albus* J1074 (Figure 5c and Figure S20). As shown in Figure 5d, out of 15 exconjugants of J1074/pBAC‐SalRefFad‐MTI1, salinomycin titers of five engineered strains were higher than those of the control strain J1074/pBAC‐SalRefFad. Repeated fermentation results showed that all five engineered strains produced higher salinomycin titers than the control. The highest salinomycin titer from J1074/pBAC‐SalRefFad‐MTI1‐9 reached up to 2.1 mg/L, with 5.3‐fold higher than the control (Figure 5e). By complete genome sequencing, we found that there was only one copy of the salinomycin BGC and its integrated sites in the five engineered strains also showed highly random genomic distributions (Figure 5f, g).

Collectively, these results demonstrated that our developed MNGE approach could efficiently achieve the non‐specific genomic integration of large‐scale metabolic pathways (i.e., the 41‐kb UK‐2 BGC and the 106‐kb salinomycin BGC) in *Streptomyces*. Distinct from the fixed‐site integration of classical site‐specific integration systems, the non‐specific integration feature of the MTI1 system would be widely used for optimizing the expression of large metabolic pathways in *Streptomyces* and other bacteria based on chromosome position effects.

### 2.4 Extending MNGE to Diverse Gram‐Positive Actinobacteria

**FIGURE 6:**
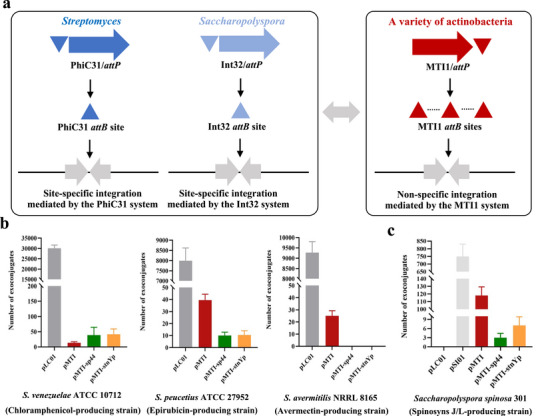
Extending MNGE to diverse Gram‐positive actinobacteria. (a) Schematic of MTI1‐mediated non‐specific integration of functional genes or metabolic pathways in a variety of actinobacteria. PhiC31‐mediated site‐specific integration in *Streptomyces* and Int32‐mediated site‐specific integration in *Saccharopolyspora* were used as the positive controls. (b) Integration efficiencies of three MTI1‐series plasmids in three industrial *Streptomyces* strains, including *S. venezuelae* ATCC 10712, *S. peucetius* ATCC 27952, and *S. avermitilis* NRRL 8165. The plasmid pLC01 was used as the positive control. (c) Integration efficiencies of three MTI1‐series plasmids in the spinosyns J/L‐producing strain *S. spinosa* 301. In the plasmids pMTI, pMTI‐*sp44*, and pMTI‐*stnYp*, the expression of the MTI1 system was under the control of the promoters ermEp*, *sp44*, and *stnYp*, respectively. The plasmids pLC01 and pSI01 were used as the negative and positive controls, respectively.

Furthermore, we tested whether the MTI1 system could work in different, important industrial *Streptomyces* as well as non‐*Streptomyces* actinobacteria (Figure 6a). As shown in Figure 6b, all three plasmids, including pMTI, pMTI‐*sp44*, and pMTI‐*stnYp*, were successfully introduced into the two industrial *Streptomyces* strains, including chloramphenicol‐producing *Streptomyces venezuelae* ATCC 10712 and epirubicin‐producing *Streptomyces peucetius* ATCC 27952. Meanwhile, pMTI was also integrated into the genome of avermectin‐producing *Streptomyces avermitilis* NRRL 8165 (Figure 6b). Although the integration efficiency of the MTI1 system was still lower than that of the PhiC31 system, our results indicated that the MNGE approach could efficiently work in a variety of industrial *Streptomyces* strains. *Saccharopolyspora spinosa* 301 is a Gram‐positive, filamentous, and genetically intractable actinobacterium, which produces the broad‐spectrum macrolide insecticide spinetoram (spinosyns J/L) [43]. Compared to the above tested *Streptomyces* strains, *S. spinosa* lacks all common site‐specific integration systems (i.e., the PhiC31 and PhiBT1 systems), although it can use a rare site‐specific Int32 integration system [20, 43]. We found that all three empty plasmids carrying the MTI1 system under the control of different promoters could be successfully introduced into *S. spinosa* 301 (Figure 6c). In particular, the conjugal efficiency of pMTI was significantly higher than that of pMTI‐*sp44* or pMTI‐*stnYp*, possibly due to the promoter ermEp*, which is from *Saccharopolyspora erythraea* and may work better than *Streptomyces*‐derived promoters *sp44* or *stnYp* in *S. spinosa* 301 (Figure 6c) [44, 45, 46]. Our results indicated that the MTI1 system could be widely used in diverse industrial *Strepmyces* and non‐*Streptomyces* rare actinobacteria. Considering that there often lacks common or identified site‐specific integration systems in a large number of important actinobacterial genera (i.e., *Actinoplanes*, *Amycolatopsis*, *Micromonospora*, and *Salinispora*) [47], the non‐specific MTI1 integration system is predicted to facilitate the expression of functional genes or metabolic pathways in diverse actinobacteria.

### 2.5 Optimizing MNGE for Production of FR900359 in Gram‐Negative Bacteria

Here, we further explored the applicability of the MNGE approach to Gram‐negative bacteria using *B. gladioli* as a representative strain. In recent years, *Burkholderia*, such as *B. gladioli* ATCC 10248 and *Burkholderia thailandensis* E264, have attracted increasing attention as an emerging source of natural products and potential microbial chassis for expressing secondary metabolites and other high‐value compounds [48]. Because a few identified LSRs were reported in *Burkholderia* until now [49], the artificial site‐specific *attB* site is usually required to be pre‐inserted into the genome of different *Burkholderia* classis for the heterologous expression of natural product BGCs‐of‐interest [50, 51]. However, the *attB* pre‐inserted process is time‐consuming, difficult, and even impossible in most genetically intractable *Burkholderia* strains. Therefore, the non‐specific MTI1 system without the pre‐introduction of *attB* sites is expected to enable accelerating gene expression and pathway engineering in *Burkholderia*.

**FIGURE 7:**
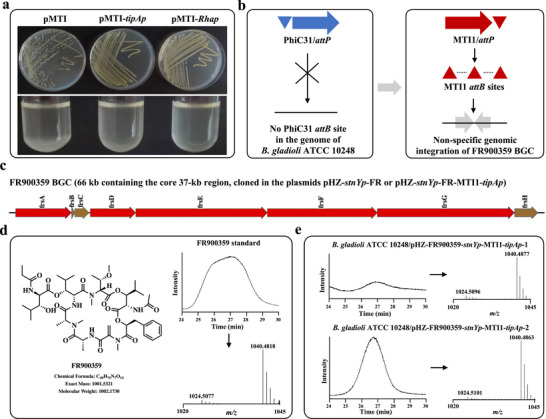
Optimizing MNGE for production of FR900359 in *B. gladioli* ATCC 10248. (a) Growth phenotypes of *B. gladioli* ATCC 10248 in the solid and liquid CYMG media when introducing different MTI1‐series plasmids. In the plasmids pMTI, pMTI‐*tipAp*, and pMTI‐*Rhap*, the expression of the MTI1 system was under the control of the promoters ermEp*, *tipAp*, and *Rhap*, respectively. (b) Schematic of MTI1‐mediated non‐specific integration of the FR900359 BGC. (c) The FR900359 biosynthetic gene cluster (BGC) from *C. vaccinii* DSM 25150. (d) The chemical structure and HRMS analysis of the standard FR900359. (e) HRMS analysis of fermentation samples of two exconjugants derived from MTI1‐mediated genomic integration of the FR900359 BGC in *B. gladioli* ATCC 10248.

However, when all three MTI1 plasmids, including pMTI, pMTI‐*sp44*, and pMTI‐*stnYp*, were introduced into *B. Gladioli* ATCC 10248, the exconjugants grew very poorly on the solid CYMG medium and could not grow in the liquid CYMG medium (Figure S21). We speculated that the overexpression of MTI1 under the control of strong promoters might lead to the obvious toxicity to *B. gladioli* ATCC 10248. Therefore, the weak inducible promoters with a certain level of basal expression, including *tipAp* (thiostrepton‐inducing promoter) from *Streptomyces* and *Rhap* (rhamnose‐inducing promoter) from *Escherichia coli*, were used to replace ermEp*, thus generating the edited plasmids pMTI‐*tipAp* and pMTI‐*Rhap*, respectively (Figure S22). As shown in Figure 7a, the exconjugants grew well both on CYMG agar plates and in liquid CYMG media when introducing either pMTI‐*tipAp* or pMTI‐*Rhap* into *B. gladioli* ATCC 10248.

The cyclic depsipeptide FR900359 of high pharmacological interest is a strong and selective inhibitor of the G_q/11_ subfamily of G protein as well as a potential lead compound for targeted therapy of tumors with oncogenic GNAQ or GNA11 mutations (encoding G_q_ and G_11_ respectively) (Figure 7) [52, 53]. Here, the MTI1 system was used for integrating the FR900359 BGC (66 kb) in the heterologous host *B. gladioli* ATCC 10248 without the native PhiC31 *attB* site (Figure 7b). First, the FR900359 BGC was cloned into the plasmid pHZ‐FR‐3C6 with the PhiC31 system by constructing a BAC library of *Chromobacterium vaccinii* DSM 25150. Then, the original promoter in the plasmid pHZ‐FR‐3C6 was replaced with the strong promoter *stnYp*, generating the edited plasmid pHZ‐*stnYp*‐FR. Furthermore, the PhiC31 system of pHZ‐*stnYp*‐FR was replaced with the MTI1 system under the control of *tipAp*, generating the edited plasmid pHZ‐*stnYp*‐FR‐MTI1‐*tipAp* (Figure 7c and Figure S23). Then, pHZ, pHZ‐*stnYp*‐FR and pHZ‐*stnYp*‐FR‐MTI1‐*tipAp* were introduced into *B. gladioli* ATCC 10248 by conjugal transfer and only 10248/pHZ‐*stnYp*‐FR‐MTI1‐*tipAp* could normally grow on CYMG agar plates or in liquid CYMG medium (Figure S24). By HPLC‐HRMS analysis of fermentation samples, two engineered strains 10248/pHZ‐*stnYp*‐FR‐MTI1‐*tipAp* successfully produced FR900359 (Figure 7d, e). Collectively, these results demonstrated that the optimized MNGE approach could mediate chromosomal integration of large‐size natural product BGC (i.e., the 66‐kb FR900359 BGC), thus achieving production of high‐value compounds in the Gram‐negative bacteria (i.e., *B. gladioli*).

## 3 Discussion

Distinct from site‐specific LSRs, MTIs have evolved transposition capabilities with relaxed sequence specificity [18]. In this study, we demonstrated for the first time that at least three MTI systems including MTI_1737, MTI_2871 and MTI_6538 could achieve genomic integration of foreign DNA cargoes in bacteria. Notably, we demonstrated that the MTI1 (MTI_2871) system could achieve non‐specific genomic integration of large‐size metabolic pathways (up to 106 kb), which transposases are not usually able to do. Therefore, the MTI1 system combines the advantages of both site‐specific LSRs for integration of large DNA cargoes and transposases for non‐specific DNA insertion. Intriguingly, we found that the MTI systems could achieve at least two‐copy, discrete insertion of mobile genetic elements in native hosts (i.e., *Amedibacterium* and *Clostridium*), which indicated that they have potential for multi‐targeting genome engineering in some important industrial microorganisms, such as *C. ljungdahlii* [24].

Based on the MTI1 system under the control of five gradient‐strength promoters, we demonstrated the design, prototyping, implementation, and application of the MNGE approach for multi‐targeting (at least three copies) and non‐specific (highly random and only requiring the core TT dinucleotide) genomic integration of metabolic genes or pathways across multiple bacterial systems. Notably, the highly unconserved *attB* site (the TT dinucleotide) theoretically allows MTI1 to target nearly any genome of bacteria and other organisms. We also tested the MTI1 system in another Gram‐negative bacteria *Chromobacterium* sp. Beijing without the common site‐specific integration systems (i.e., the PhiC31 and PhiBT1 system), which could produce the anti‐tumor drug FK228 (Figure S25) [54, 55]. When all three MTI1 plasmids, including pMTI, pMTI‐*sp44*, and pMTI‐*stnYp*, were introduced into *Chromobacterium* sp. Beijing, the exconjugants grew poorly on the solid CYMG medium and could not grow in the liquid CYMG medium (Figure S26a). By contrast, the exconjugants could grow well both on solid CYMG agar plates and in liquid CYMG media when introducing either pMTI‐*tipAp* or pMTI‐*Rhap* into *Chromobacterium* sp. Beijing (Figure S26b). Collectively, our results indicated that the MTI1 system showed a certain degree of toxicity to *Streptomyces*, *Burkholderia*, and *Chromobacterium*, possibly due to its non‐specific integration features. On the other side, because the integration efficiency of the MTI1 system was far lower than that of the PhiC31 system (Figures 1d and 2c), homologous recombination, rather than MTI1‐mediated integration, could play a dominant role when a natural product BGC is introduced into a bacterial strain that contains this BGC. More versatile MTI systems with lower toxicity and higher integration efficiency will be worthy of being developed via the identification of novel MTIs or directed evolution of the known MTI1 system for the broad applicability of the MNGE approach in the near future.

Finally, we demonstrated the broad applicability of the MNGE approach for next‐generation genome engineering in diverse bacterial hosts, thus efficiently increasing the fermentation levels of small molecule drugs based on chromosomal position effects. Notably, UK‐2 titers of the three engineered strains J1074/pCAP‐UK‐MTI1‐1, 5, and 7 over five‐round passages were comparable to those of their corresponding parental strains (Figure 4b and Figure S27a). Similarly, over five successive sub‐cultivation tests, salinomycin titers of the five engineered strains J0174/pBAC‐SalRefFad‐9, 10, 11, 12, and 15 were not significantly decreased compared to their starting strains (Figure 5e and Figure S27b). These results highlighted the genetic stability of these engineered strains constructed by the MNGE approach. Actually, the unidirectional nature of MTI1‐mediated genomic integration in a bacterial genome theoretically ensures the generation of stable, high‐yield engineered strains. Although only one copy of large‐size natural product BGCs (i.e., the 41‐kb UK‐2 BGC and the 106‐kb salinomycin BGC) was randomly integrated into a heterologous host, different MTI systems (i.e., MTI1, MTI_1737, and MTI_6538) could be used in a combined manner for genomic integration of large metabolic pathways with multiple copy numbers in a single step [20]. Comparison of MNGE and other methods (i.e., MSGE, aMSGE, and PASTE) for multi‐copy genomic integration of large DNA fragments was summarized in Table S4 [19, 20, 56, 57]. We believe that further application of the MNGE approach in other bacteria is feasible for use in a variety of scenarios, ranging from the early‐stage high‐throughput discovery of novel beneficial small molecules to the late‐stage cost‐efficient, sustainable production of high‐value compounds [10, 58, 59, 60].

## 4 Methods

### 4.1 Strains, Plasmids, and Growth Conditions

The strains and plasmids used in this study are listed in Tables S1 and S2, respectively. *S. albus* J1074, *S. coelicolor* M1152, *S. lividans* SBT5, *S. lividans* RedStrep1.7, *S. venezuelae* ATCC 10712, *S. avermitilis* NRRL 8165, *S. huiliensis* GDMCC 4.215, and their derivatives were grown on MS agar medium (g/L, soybean flour 20, mannitol 20, and agar 20) for spore preparation and intergeneric conjugal transfer. *S. atratus* SCSIO ZH16NSEP‐Δligase (ZH16) and its derivatives were grown on YMS medium (g/L, yeast extract 4, malt extract 10, soluble starch 4, oat 7.5, CaCO_3_ 2, agar 20, pH 7.4). *S. peucetius* ATCC 27952 and its derivatives were grown on MY medium (g/L, maltodextrin 20, yeast extract 15, casamino acid 5, glucose 4, peptone 2.5, K_2_HPO_4_ 1.5, CaCO_3_ 2, agar 20, pH 7.2‐7.4) for mycelium preparation and intergeneric conjugal transfer. *S. spinosa* 301 and its derivatives were grown on 2CMC agar medium (g/L, soluble starch 10, tryptone 2, NaCl 1, (NH_4_)_2_SO_4_ 2, K_2_HPO_4_ 1, MgSO_4_·7H_2_O 2, casamino acid 2, CaCO_3_ 2, agar 20 and addition of trace element solution including MnCl_2_·4H_2_O 1.55 mg/L, ZnSO_4_·7H_2_O 1.75 mg/L, pH 7.2) for spore preparation and intergeneric conjugal transfer. *B. gladioli* ATCC 10248 and *Chromobacterium* sp. Beijing was grown on CYMG medium (g/L, tryptone 8, yeast extract 4, MgCl_2_·2H_2_O 4.06, glycerol 10, agar 20) with 50 µg/mL gentamicin. *B. gladioli* ATCC 10248 and *Chromobacterium* sp. Beijing was grown on CYMG liquid medium for intergeneric conjugal transfer.

The MS medium with 10 mM MgCl_2_ was used for conjugal transfer from *E. coli* to *S. albus* J1074, *S. coelicolor* M1152, *S. lividans* SBT5, or *S. avermitilis* NRRL 8165. The M‐Isp4 medium (g/L, soybean flour 5, mannitol 5, starch 5, tryptone 2, yeast extract 1, NaCl 1, (NH_4_)_2_SO_4_ 2, K_2_HPO_4_ 1, CaCO_3_ 2, agar 20, and trace element solution 1 mL, pH 7.0–7.2; Trace Element solution: ZnSO_4_·7H_2_O 1 g/L, FeSO_4_·7H_2_O 1 g/L and MnSO_4_·H_2_O 1 g/L) with 10 mM MgCl_2_ was used for conjugal transfer from *E. coli* to *S. lividans* RedStrep1.7, *S. venezuelae* ATCC 10712, *S. peucetius* ATCC 27952 or *S. atratus* SCSIO ZH16NSEP‐Δligase (ZH16). The 2CMC medium with 20 mM MgCl_2_ was used for conjugal transfer from *E. coli* to *S. spinosa* 301. LB with 0.5 mm diaminoheptanedioic acid (DAP) and CYMG with 50 µg/mL apramycin were used for conjugal transfer from *E. coli* to *B. gladioli* ATCC 10248 or *Chromobacterium* sp. Beijing. The liquid TSB medium was used for genomic DNA isolation of *S. huiliensis* GDMCC 4.215, *S. albus* J1074, and its derivatives. The liquid CYMG medium was used for genomic DNA isolation of *Chromobacterium* sp. Beijing. All *Streptomyces*, *S. spinosa*, *B. gladioli*, and *Chromobacterium* strains were cultivated at 30°C.

*E. coli* DH5α and EPI300 were used for DNA cloning. *E. coli* DH10B was used for cloning the FR900359 BGC by constructing a BAC library. *E. coli* BL23 or *E. coli* DH10B/pCB006 was used for editing the large‐sized natural product BGCs (i.e., the UK‐2 BGC and salinomycin BGC). *E. coli* ET12567/pUZ8002 or S17‐1 was used for conjugal transfer from *E. coli* to *Streptomyces*. *E. coli* S17‐1 was used for conjugal transfer from *E. coli* to *S. spinosa* 301. *E. coli* WM3064, as a nutrient‐deficient strain of DAP, was used for conjugal transfer from *E. coli* to *B. gladioli* ATCC 10248 or *Chromobacterium* sp. Beijing. Integration efficiency of each plasmid was defined as the number of exconjugants obtained after conjugation from the donor *E. coli* strain to a recipient strain (various *Streptomyces* strains, *S. spinosa*, *B. gladioli*, and *Chromobacterium*), using standardized cell quantities for both donor and recipient. *E. coli* strains were grown at 37°C in LB liquid medium or on LB agar plates. Antibiotics (50 µg/mL ampicillin, apramycin, chloramphenicol, gentamicin, kanamycin, or spectinomycin) were added when necessary. *Saccharomyces cerevisiae* V6‐48 was used for capturing the UK‐2 BGC. *S. cerevisiae* strains were grown at 30°C on solid or in liquid YPD media (g/L, glucose 2, yeast extract 1, peptone 2, and adenine sulfate 0.08).

### 4.2 Construction of MTI‐Series Plasmids

The MTI_1737, MTI_2871 (MTI1), MTI_6538, and MTI_Cp36 multi‐targeting non‐specific integration systems, each of which consists of an integrase gene and its corresponding *attP* site, were chemically synthesized after code optimization for expression in bacteria possessing high GC content genomes, such as *Streptomyces* and *Burkholderia*. First, the DNA fragment ermEp*‐MTI_2871 was obtained by double‐digesting the plasmid pUC57‐MTI_2871 with *EcoR* I and *Hind* III. Using the plasmid pLC01 as the template, the plasmid pLC01 skeleton was obtained by PCR with the primer pair pLC‐skeleton‐*Hind*III‐fw/pLC‐skeleton‐*EcoR*I‐rev. Then, the above two fragments were assembled through in vitro recombination using DNA Assembly Mix Plus (ShareBio Biotech, China), thus generating the plasmid pMTI_2871 (pMTI). Using the plasmid pMTI as the template, the plasmid pMTI skeleton was obtained by PCR with the primer pair MTI‐fw and pMTI‐skeleton‐rev. The promoters *sp44*, *stnYp*, *tipAp*, and *Rhap* were obtained by PCR with the primer pairs skeleton‐*sp44*‐fw/*sp44*‐MTI‐rev, skeleton‐*stnYp*‐fw/*stnYp*‐MTI‐rev, MTI‐*tipAp*‐fw/*tipAp*‐MTI‐rev, and MTI‐*Rhap‐*fw/*Rhap*‐MTI‐rev using the plasmids pSET‐*sp44‐indC*, pSET‐*stnYp‐indC*, pKCCas9dO, and pBBR1‐Rha‐Km‐Redγ‐BAS as the templates, respectively. Then, the plasmid pMTI skeleton and different promoters were assembled through in vitro recombination, thus generating the plasmids pMTI‐*sp44*, pMTI‐*stnYp*, pMTI‐*tipAp*, and pMTI‐*Rhap*, respectively. Using the plasmid pMTI as the template, the plasmid pMTI skeleton was obtained by PCR with the primer pair pMTI‐skeleton‐fw/ermEp*‐rev. The codon‐optimized MTI_1737 was obtained by PCR with the primer pair ermEp*‐1737‐fw/1737‐rev. The two PCR products were assembled through in vitro recombination, thus generating the plasmid pMTI_1737. The plasmids pMTI_6538, pMTI_Cp36, pMTI_1737‐*stnYp*, pMTI_6538‐*stnYp*, and pMTI_1737‐*stnYp* were generated using a similar strategy. The plasmid skeletons pMTI‐*Xba*I or pMTI‐*stnYp‐Xba*I were obtained by digesting the plasmid pMTI or pMTI‐*stnYp* with *Xba* I. The reporter gene cassette *idgS‐sfp* was obtained by PCR with the primer pair *Xba*I‐*idgS*‐fw/*idgS*‐ter‐rev using the plasmid Tn315‐*idgS* as the template. The two DNA fragments were assembled through in vitro recombination, thus generating the plasmids pMTI‐*idgS* or pMTI‐*stnYp*‐*idgS*.

### 4.3 Cloning of the UK‐2 BGC and FR900359 BGC

The UK‐2 BGC was cloned from the genome of *S. huiliensis* GDMCC 4.215 using the CRISPR/Cas9‐assisted TAR cloning approach in yeast as described previously, and pCAP01 was used as the cloning vector [35]. The FR900359 BGC was cloned from the genome of *Chromobacterium vaccinii* DSM 25150 using the approach for the construction of a BAC library as described previously, and pHZ was used as the cloning vector [61, 62]. The two detailed BGC cloning procedures were described in the supporting information.

### 4.4 Editing of the Plasmids pCAP‐UK, pBAC‐SalRefFad, and pHZ‐FR‐3C6

The iCASRED approach that we developed previously was used for the editing of the plasmid pCAP‐UK containing the UK‐2 BGC to generate the plasmids pCAP‐UK‐MTI1 or pCAP‐UK‐BT1 [39]. Briefly, the plasmid pCB003 skeleton and the transcription cassette of sgRNA targeting the PhiC31 gene were obtained by PCR using the primer pairs pCB003‐skeleton‐fw/rev and PhiC31‐gRNA‐fw/sgRNA‐rev, respectively. Then, the two PCR products were assembled through in vitro recombination, thus generating the plasmid pCB003‐C31. The DNA fragments *acc(3)IV*‐MTI1 and *acc(3)IV*‐BT1 were obtained by PCR using the primer pairs *acc(3)IV*‐MTI1‐fw/rev and *acc(3)IV*‐BT1‐fw/rev, respectively. Second, the plasmid pCAP‐UK was electroporated into *E. coli* BL23 and the single colony of BL23/pCAP‐UK were inoculated into 4 mL LB liquid medium. After growth overnight at 37°C, 100 µL cultures were transferred to 50 mL LB liquid medium in 250 mL flasks. When OD_600_ reached ∼0.3, 10 mM arabinose was added to induce the expression of the λRed system for 0.5 h. The cultures were collected and washed twice with 10% glycerol. Both the editing plasmid pCB003‐C31 (100–200 ng) and the DNA fragments *acc(3)IV*‐MTI1 or *acc(3)IV*‐BT1 (300–500 ng) were electroporated into the competent cells, followed by growth overnight at 37°C on an LB agar plate with 50 µg/mL kanamycin and 50 µg/mL spectinomycin. After each transformation experiment, 15 colonies were randomly selected by PCR using the primer pairs (i.e., ID‐MTI1‐fw/rev and ID‐BT1‐fw/rev), followed by Sanger sequencing, thus generating the plasmids pCAP‐UK‐MTI1 or pCAP‐UK‐BT1. Similarly, the iCASRED approach was also used for the editing of the plasmids pBAC‐SalRefFad and pHZ‐FR‐3C6 [39]. The detailed BGC editing procedures were described in the Supporting Information.

### 4.5 Integrating Genes or BGCs Into *Streptomyces* and *S. spinosa*

The conjugation approach was used for integrating genes or large BGCs from *E. coli* ET12567/pUZ8002 or S17‐1 into *Streptomyces* or *S. spinosa*. Briefly, the empty plasmids with apramycin resistance (i.e., pLC01 and pMTI), the *idgS* expression plasmids with apramycin resistance (i.e, pLC01‐*idgS* and pMTI‐*idgS*), and the BGC expression plasmids with apramycin resistance (i.e., pCAP‐UK and pCAP‐UK‐MTI1) were transferred into *E. coli* ET12567/pUZ8002 by electroporation. The plasmids with kanamycin resistance (i.e., pCAP01, Tn315, and Tn315‐*idgS*) and the BGC expression plasmids with kanamycin resistance (i.e., pCAP‐UK) were transferred into *E. coli* S17‐1 by electroporation. Then, a single clone of the donor cells *E. coli* ET12567/pUZ8002 or S17‐1 containing a target plasmid was grown overnight in LB with 50 mg/L apramycin/chloramphenicol/kanamycin, and then inoculated into fresh LB broth and grown to an OD_600_ of 0.4‐0.6. Spores of different *Streptomyces* strains and spores of *S. spinosa* were used as the recipient cells. The recipient and donor cells were mixed and inoculated on different agar media with 10 or 20 mM MgCl_2_ as discussed above. The plates were incubated at 30°C for 16–18 h and then overlaid with 1 mL of sterile water containing nalidixic acid and apramycin (or kanamycin). After growth of 5–7 days for *Streptomyces* and 12–14 days for *S. spinosa*, the correct exconjugants were screened by colony PCR validation followed by Sanger sequencing.

### 4.6 Integrating Genes or BGCs Into *B. gladioli* ATCC 10248 and *Chromobacterium* sp. Beijing

The conjugation approach was used for integrating genes or large BGCs from *E. coli* WM3064 into or *B. gladioli* ATCC 10248 and *Chromobacterium* sp. Beijing. Briefly, the empty plasmids (i.e., pHZ, pMTI, pMTI‐*sp44*, pMTI‐*stnYp*, pMTI‐*tipAp*, and pMTI‐*Rhap*) and the BGC expression plasmids (pHZ‐s*tnYp*‐FR and pHZ‐*stnYp*‐FR‐MTI1*‐tipAp*) were transferred into *E. coli* WM3064 by electroporation and then incubated on LB agar medium with 0.5 mM DAP with *B. gladioli* ATCC 10248 and *Chromobacterium* sp. Beijing or at 37°C for 6 h. Then, a portion of the mixed strains was plated on CYMG agar plates containing 50 mg/L apramycin at 30°C for 3 days for *B. gladioli* ATCC 10248 or *Chromobacterium* sp. Beijing. Finally, the correct exconjugants were screened by colony PCR validation followed by Sanger sequencing.

### 4.7 Fermentation of *Streptomyces* Strains and HPLC (or LC‐MS) Analysis of Indigoidine, UK‐2A, and Salinomycin

Fermentation of *Streptomyces albus* strains, as well as production and measurement of indigoidine, were performed as described previously [46]. Briefly, *S. albus* strains were incubated in 5 mL of TSB seed broth in a 13‐mL shake tube for 24–30 h at 30 °C. Then, 2 mL of seed cultures was transferred into 50‐mL fermentation R5a broth (g/L, sucrose 100, glucose 10, yeast extract 5, MgCl_2_·6H_2_O 10.12, K_2_SO_4_ 0.25, casamino acids 0.1, MOPS 21, NaOH 2, Trace Element solution 2 mL, pH 6.85; Trace Element solution: CaCl_2_ 5.88 mg/L, ZnCl_2_ 80 µg/L, FeCl_3_·6H_2_O 400 µg/L, MnCl_2_ 20 µg/L, CuCl_2_ 20 µg/L, Na_2_B_4_O_7_·10H_2_O 20 µg/L, (NH_4_)_6_Mo_7_O_24_·4H_2_O 20 µg/L) in a 250‐mL Erlenmeyer flask at 30 °C and 200 rpm. After 1–5 days, 200 µL fermentation cultures were collected and diluted using 1.8 mL methanol (MeOH), followed by centrifugation at 12 000 rpm for 5 min. The supernatants were measured at OD_600_.

Fermentation of *Streptomyces* strains, as well as production and detection of UK‐2, were performed as described previously [32]. Briefly, spore suspensions of *S. albus*, *S. lividans*, *S. atratus*, or *S. huiliensis* strains were incubated in 50 mL of TSB broths in a 250‐mL shake flask on an orbital shaker (200 rpm) for 24 h at 30 °C. Then, 2 mL of cultures was transferred into 50‐mL fermentation broths, including R5a, MS (g/L, soybean flour 20, mannitol 20), A3M (g/L, glucose 5, glycerol 20, starch 20, cottonseed flour 15, yeast extract 3, pH 7.0‐7.2), GYM (g/L, yeast extract 4, malt extract 10, glucose 4, peptone 1, NaCl 2, ph 7.2‐7.4) and ISP2 (g/L, yeast extract 4, malt extract 10, glucose 4), in a 250‐mL Erlenmeyer flask at 30 °C and 200 rpm. After 2–6 days, 750 µL fermentation cultures were collected and extracted with 750 µL ethyl acetate/petroleum ether (v/v = 1:1), followed by centrifugation at 4,000 rpm for 5 min. 500 µL supernatants were concentrated under vacuum, followed by resuspension in 100 µL DMSO for HPLC analysis (1260 series, Agilent) using a 4.6 × 100 mm XBridge C18 column (5 µm, Waters). For HPLC detection, a water: acetonitrile (ACN) gradient was used as the mobile phase: min 0, 50% ACN; min 2, 50% ACN; min 23, 95% ACN; min 27, 15% ACN; min 29, 50% ACN; and min 30, 50% ACN. The flow rate was 1.0 mL/min, the eluate was monitored at 231 nm, and the column temperature was 30°C. UK‐2A, UK‐2B, and UK‐2CD eluted at approximately 9.1, 10.4, and 11.1 mins, respectively.

Fermentation of *Streptomyces* strains, as well as production and detection of salinomycin, were performed as described previously [42]. Briefly, spore suspensions of *S. albus* strains were incubated in 50 mL TSB broths in a 250‐mL shake flask on an orbital shaker (200 rpm) for 24 h at 30°C. Then, 3 mL of cultures was transferred into 50 mL fermentation broths (g/L, germ powder 8, soybean powder 5, potassium chloride 2, sodium chloride 1, urea 1.6, tartaric acid 2, MgSO_4_ 0.1, KH_2_PO_4_ 0.1, CaCO_3_ 5, soybean oil 150, pH 6.6‐6.9), in a 250‐mL Erlenmeyer flask at 30°C and 200 rpm. After three days, 2% (v/v) Amberlite XAD‐16 resin was added to the fermentation broths and incubated for another 24 h. Both bacterial cells and resin were collected and then extracted with 30 mL of methanol for 3 h with shaking. The extracts were evaporated and dissolved in 0.5 mL methanol for HPLC‐HRMS analysis (1260 series, Agilent) with a 4.6 × 150 mm XBridge C18 column (5 µm, Agilent) using an Agilent 1290 Series HPLC coupled to a 6545 Series QTOF mass spectrometer and controlled by Masshunter software. For HPLC detection, a water: acetonitrile (ACN) gradient was used as the mobile phase: min 0, 80% ACN; min 5, 80% ACN; min 12, 100% ACN; min 23, 100% ACN; min 29, 80% ACN; and min 30, 80% ACN. The flow rate was 1.0 mL/min, the eluate was monitored at 210 nm, and the column temperature was 30°C. Salinomycin eluted at approximately 18.8 min.

### 4.8 Scale‐up Fermentation, Extraction, and Isolation of UK‐2A, UK‐2B, and UK‐2CD

Spore suspensions of *S. albus* J1074/pCAP‐UK‐C1 were inoculated into 50 mL of TSB and shaken for 1 day at 30°C and 200 rpm. 20 mL of seed culture was transferred into 500 mL of MS fermentation broth in a 2‐L shake flask. After 4 days of shaking at 30°C and 200 rpm, 4.5 L cultures were extracted with 4.5 L of ethyl acetate/petroleum ether (v/v = 1:1) and concentrated under vacuum, resulting in 1.31 g of crude extract. Then, the crude extract was packed into Sephadex LH‐20 chromatography and eluted with methanol. Subsequently, the combined UK‐2‐containing subfraction (840.5 mg) from the Sephadex LH‐20 column was subjected to HPLC chromatography equipped with a XBridge BEH Prep C18 (10 × 150 mm, 5 µm, 130 Å) to afford pure UK‐2A (21.8 mg, *t*_R_ = 9.1 min), UK‐2B (2.9 mg, *t*_R_ = 10.4 min) and UK‐2CD (10.5 mg, *t*_R_ = 11.1 min). For all liquid chromatography, a water: acetonitrile (ACN) gradient was used as follows: min 0, 50% ACN; min 2, 50% ACN; min 23, 95% ACN; min 27, 15% ACN; min 29, 50% ACN and min 30, 50% ACN; flow rate 2 mL/min, 25°C. The purities of UK‐2A, UK‐2B, and UK‐2CD were confirmed by HPLC analysis (Figure S15a). The identities of UK‐2A, UK‐2B, and UK‐2CD were further confirmed by LC‐MS analysis using an Agilent 1290 Series HPLC coupled to a 6545 Series QTOF mass spectrometer equipped with a 4.6×150 mm XBridge C18 column (5 µm, Waters) and controlled by Masshunter software. Purified UK‐2A, UK‐2B, and UK‐2CD were used to generate their corresponding standard curves, as shown in Figure S14. UK‐2 production was calculated from these standard curves.

### 4.9 Fermentation of *Burkholderia* Strains and LC‐MS Analysis of FR900359

*B. gladioli* ATCC 10248 with the plasmid pHZ‐*stnYp*‐FR‐MTI1‐*tipAp* was inculated into 5 mL CYMG broths in 13‐mL shake tubes at 30°C and 200 rpm for 12 h. Then, 1 mL overnight cultures were inoculated into 50 mL M9 broths (g/L, glucose 17.89, tryptone 34.98, K_2_HPO_4_·3H_2_O 20.44, KH_2_PO_4_ 4.4, sodium citrate 0.01, pH 7.0) in a 250‐mL shake flask at 30°C and 200 rpm After 48 h, 20 mL fermentation cultures were collected and extracted with the same volume of n‐butanol, followed by centrifugation at 12,000 rpm for 10 min. 20 mL supernatants were concentrated under vacuum, followed by dissolution in 200 µL MeOH for HPLC‐HRMS analysis (1260 series, Agilent) with a 4.6 × 250 mm XBridge C18 column (5 µm, Dikoma) using an Agilent 1290 Series HPLC coupled to a 6545 Series QTOF mass spectrometer and controlled by Masshunter software. The flow rate was 1.0 mL/min, the eluate was monitored at 210 nm, and the column temperature was 30°C. FR900359 eluted at approximately 29.0 min.

### 4.10 Sequencing of the Complete Genome of *S*. *h* *uiliensis* GDMCC 4.215

To access the complete peptide sequences encoded by the UK‐2 BGC, we re‐sequenced the genomes of *S. huiliensis* GDMCC 4.215 using both the third‐generation PacBio RSII Sequencing platform and the second‐generation MGI's DNBSEQ‐T7 platform (BGI Genomics). For sample preparation (0.5–1 g cells), *S. huiliensis* GDMCC 4.215 was cultured in TSB broth for 2 days. Then, bacterial cells (0.5–1 g) were harvested from the corresponding cultures by centrifugation at 12 000 rpm for 10 min. The complete genome was assembled using the Bacteria Genome Sequencing (CompleteGenome) Analysis Method (for the PacBio platform). Finally, antiSMASH 7.1.0 was used to analyze the assembled bacterial genomes for detecting the complete UK‐2 BGC.

Sequencing of the genomes of different *S. albus* J1074/pMTI‐*idgS*, *S. albus* J1074/pCAP‐UK‐MTI1, *S. albus* J1074/pCAP‐UK‐C2‐MTI1 and *S. albus* J1074/pBAC‐SalRefFad‐MTI1 strains to identify the chromosomal integration sites of *idgS‐sfp*, genomes were extracted from different *S. albus* J1074/pMTI‐*idgS* strains and sequenced by the second‐generation MGI's DNBSEQ‐T7 platform (BGI Genomics). For sample preparation, *S. albus* J1074/pMTI‐*idgS* was cultured in TSB broths for 2 days, and then cells (0.1–0.2 g) were harvested from cultures by centrifugation at 12 000 rpm for 10 min. Then, the sequenced genomes were assembled using the Bacteria Genome Sequencing (DraftGenome) Analysis Method. DNBSEQ‐T7 sequencing data using Trimmomatic v0.39 for quality control (removing bases with quality <20, min length 75 bp) were used for genome assembly, followed by SPAdes v3.15 in isolate mode for de novo assembly based on paired‐end and unpaired reads. The chromosomal integration sites of the UK‐2 BGC in *S. albus* J1074/pCAP‐UK‐MTI1 or *S. albus* J1074/pCAP‐UK‐C2‐MTI1, as well as the salinomycin BGC in *S. albus* J1074/pBAC‐Sal‐RefFad‐MTI1, were also confirmed using a similar genome sequencing strategy.

### 4.11 Statistical Analysis

For each experiment, three independent biological replicates (n = 3) were used unless stated otherwise. Data were shown as mean values ± s.e.m and no data were excluded from the statistical analyses.

## Author Contributions

R.Z.S. and R.X.Y. designed the experimental plan and performed the majority of the experiments presented; Y.W.Z. edited the FR900359 BGC and performed the fermentation of *B. gladioli* ATCC 10248 for production of FR900359; H.Y.H. and W.F.W. performed the conjugal transfer in *S. peucetius* and *S. spinosa*; X.D.Q. assisted project management and edited the manuscript; Y.H.L. designed the experimental plan and edited the manuscript; L.L. contributed to project management, designed the experimental plan, and wrote the manuscript. All authors approved the final submission.

## Conflicts of Interest

The authors declare no conflict of interest.

## Supporting information




**Supporting File**: advs74503‐sup‐0001‐SuppMat.pdf.

## Data Availability

The data that support the findings of this study are available in the supplementary material of this article.
