# Supplementary material for: Multi‐Targeting Non‐Specific Genome Engineering in Bacteria
Source: Adv Sci (Weinh). 2026 Mar 9;13(25):e21532. doi: 10.1002/advs.202521532 (PMC13137831; doi:10.1002/advs.202521532)
Supplement: Supplementary file 1 — Supporting File: advs74503‐sup‐0001‐SuppMat.pdf. [file ADVS-13-e21532-s001.pdf]

Supporting Information for:

Title: Multi-Targeting Non-Specific Genome Engineering in Bacteria

Authors: Runze Sun[1#], Ruixiang You[1,2#], Yiwen Zhou[1,2], Huiyan He[2], Wenfang Wang[2], Xudong Qu[1], Yinhua Lu[2*], Lei Li[1*]

Author affiliation:

[1] State Key Laboratory of Microbial Metabolism and School of Life Sciences and Biotechnology, Shanghai Jiao Tong University, China

[2] College of Life Sciences, Shanghai Normal University, China

Corresponding author: Lei Li and Yinhua Lu

Lead contact: School of Life Sciences and Biotechnology
Shanghai Jiao Tong University
800 Dongchuan Road
Shanghai, 200240

Email: lei.li@sjtu.edu.cn (Lei Li)

Contact: School of Life Sciences
Shanghai Normal University
100 Guilin Road
Shanghai, 200234

Email: yhlu@shnu.edu.cn (Yinhua Lu)

Table of Contents:

1. Table S1: Microbial strains used in this study

2. Table S2: Plasmids used in this study

3. Table S3: Oligonucleotide sequences used in this study

4. Table S4: Comparison of integrase-based MNGE and other methods for multi-copy genomic integration of large DNA fragments

5. Figure S1: Protein sequence, codon-optimized gene sequence and *attP* site of MTI_1737

6. Figure S2: Protein sequence, codon-optimized gene sequence and *attP* site of MTI_2871 (MTI1)

7. Figure S3: Protein sequence, codon-optimized gene sequence and *attP* site of MTI_6538

8. Figure S4: Protein sequence, codon-optimized gene sequence and *attP* site of MTI_Cp36

9. Figure S5. Schematic of an alignment of diverse *attB* sequences that are targeted by four tested MTIs

10. Figure S6: Genetic maps of eight MTI-series plasmids

11. Figure S7: Chromosomal positions for MGE integration mediated by four different MTIs in native hosts

12. Figure S8: Genetic maps of the *idgS-sfp*-expressing plasmids pLC01-*idgS*, Tn315-*idgS*, pMTI-*idgS* and pMTI-*stnYp*-*idgS*

13. Figure S9: Integration of *idgS-sfp* mediated by MTI1 under the control of two different strong promoters in *S. albus*

14. Figure S10: Indigoidine titers and growth phenotypes of 14 MTI1-mediated, *idgS-sfp*-integrated *S. albus* exconjugants

15. Figure S11: MTI1-mediated integration sites and copy numbers of *idgS-sfp* in 14 independent *S. albus* exconjugants

16. Figure S12: MTI1-mediated integration of *idgS-sfp* in 140 independent *S. albus* exconjugants

17. Figure S13: MTI1-mediated integration sites and copy numbers of *idgS-sfp* in 14 independent *S. albus* exconjugants that high-efficiently produced indigodine

18. Figure S14: Standard curves of UK-2A, UK-2B and UK-2CD

19. Figure S15: HPLC analysis of UK-2 standards and UK-2 production in *S. huiliensis* GDMCC 4.215

20. Figure S16: Genetic map and antiSMASH analysis of the complete genome of *S. huiliensis* GDMCC 4.215

21. Figure S17: Biosynthetic gene cluster and proposed pathway of UK-2

22. Figure S18: Restriction analysis of pCAP-UK (a), pCAP-UK-MTI1 (b) and pCAP-UK-BT1 (c)

23. Figure S19: HPLC analysis of UK-2 production in four heterologous *Streptomyces* hosts

24. Figure S20: Identification of the edited plasmid pBAC-SalRefFad-MTI1

25. Figure S21: Growth phenotypes of *B. gladioli* ATCC 10248 when introducing three strong promoters controlled

MTI1-series plasmids

26. Figure S22: Genetic maps of the plasmids pMTI, pMTI-*tipAp* and pMTI-*Rhap*

27. Figure S23: Identification of the plasmids pHZ-*stnYp*-FR and pHZ-*stnYp*-FR-MTI1-*tipAp*

28. Figure S24: Growth phenotypes of *B. gladioli* ATCC 10248 when introducing pHZ, pHZ-*stnYp*-FR or pHZ-*stnYp*-FR-MTI1-*tipAp*

29. Figure S25: LC-MS analysis of FK228 standard and FK228 production in *Chromobacterium sp.* Beijing

30. Figure S26: Growth phenotypes of *Chromobacterium* sp. Beijing when introducing strong promoters (a) or weak promoter (b) controlled MTI1-series plasmids

31. Figure S27. Confirmation of MTI1-mediated exconjugants that high-efficiently produced UK-2 (a) and spinosyns J/L (b) after five-round passages.

32. Figure S28: UK-2 production of *S. albus* J1074/pCAP-UK-C2-MTI-7 and *S. huiliensis* GDMCC 4.215 when feeding L-Valine

33. Extended methods

Table S1. Microbial strains used in this study

Strains	Relevant features	Source
Escherichia coli		
BL23	The DNA editing strain with the integration of the Cas9/λRed expression cassettes, *recA* deletion and simultaneously insertion of the *lacI*^{q} expression cassette and the Ptrc-driven transcription cassette of sgRNA targeting the spectinomycin resistance gene of the editing plasmid pCB003	Zheng et al., 2025
DH5α	F^- 80Φ*dlacZ D*M15Δ(*lacZYA−argF*)*U169deoR recA1 endA1 hsdR17* (rk$^-$ mk$^+$) *supE44 λ*$^-$*thi*$^-$*1gyrA96 relA1*	Our lab
DH10B	F^- *mcr*A Δ(*mrr-hsd*RMS-*mcr*BC) Φ80*lac*ZΔM15 Δ*lac*X74 *rec*A1 *end*A1 *ara*D139Δ(*ara*, *leu*)7697 *gal*U *gal*K λ$^-$*rps*L *nup*G *ton*A	Our lab
EPI300	F^- *mcrA* Δ(*mrr-hsdRMS-mcrBC*) Φ80*dlacZ*ΔM15 Δ*lacX74 recA1 endA1 araD139* Δ(*ara, leu*)*7697 galU galK λ- rpsL* (*StrR*) *nupG trfA ton*	Our lab
ET12567/pUZ8002	ET12567 containing the non-transmissible RP4 derivative plasmid pUZ8002	Our lab
S17-1	*supE44* Δ*lacU169* (Φ*lacZ*ΔM15) *recA1 endA1 hsdR17 thi-1 gyrA96 relA1 par* phage lysogenic	Our lab
WM3064	A diaminoheptanedioic acid-deficient donor strain for conjugate transfer with *Chromobacterium* sp. Beijing or *Burkholderia gladioli* ATCC 10248	Bai et al., 2023
Saccharomyces cerevisiae		
V6-48	Host strain for *in vivo* homologous recombination: *MAT alpha, his3-D200, trp1-D1, ura3-52, lys2, ade2-101, met14, psi + cir0*	Yamanaka et al., 2014
Streptomyces albus		
J1074	Model *Strptomyces, S. albus* G mutant	Chater and Wilde, 1976
J1074/pLC01	J1074 containing the plasmid pLC01	This study
J1074/Tn315	J1074 containing the plasmid Tn315	This study
J1074/pMTI_1737	J1074 containing the plasmid pMTI_1737	This study
J1074/pMTI_1737-*stnYp*	J1074 containing the plasmid pMTI_1737-*stnYp*	This study
J1074/pMTI_2871 (pMTI)	J1074 containing the plasmid pMTI_2871 (pMTI)	This study
J1074/pMTI_2871-*stnYp*	J1074 containing the plasmid pMTI_2871-*stnYp*	This study
J1074/pMTI_6538	J1074 containing the plasmid pMTI_6538	This study
J1074/pMTI_6538-*stnYp*	J1074 containing the plasmid pMTI_6538-*stnYp*	This study
J1074/pMTI_Cp36	J1074 containing the plasmid pMTI_Cp36	This study
J1074/pMTI_Cp36-*stnYp*	J1074 containing the plasmid pMTI_Cp36-*stnYp*	This study
J1074/pLC01-*idgS*	J1074 containing the plasmid pLC01-*idgS*	This study
J1074/Tn315-*idgS*	J1074 containing the plasmid Tn315-*idgS*	This study
J1074/pMTI-*idgS*	J1074 containing the plasmid pMTI-*idgS*	This study
J1074/pMTI-*stnYp-idgS*	J1074 containing the plasmid pMTI-*stnYp-idgS*	This study
J1074/pCAP01	J1074 containing the plasmid pCAP01	This study
J1074/pCAP-UK-C1	J1074 containing the one-copy of plasmid pCAP-UK	This study
J1074/pCAP-UK-C2	J1074 containing the two copies of plasmid pCAP-UK	This study
J1074/pCAP-UK-MTI1	J1074 containing the plasmid pCAP-UK-MTI1	This study
J1074/pCAP-UK-C2-B1	J1074/pCAP-UK-C2 containing the one-copy of the plasmid pCAP-UK-BT1	This study
J1074/pCAP-UK-C2-MTI1	J1074/pCAP-UK-C2 containing the plasmid pCAP-UK-MTI1	This study
J1074/pBAC-SalRefFad	J1074 containing the plasmid pBAC-SalRefFad	This study
J1074/pBAC-SalRefFad-MTI1	J1074 containing the plasmid pBAC-SalRefFad-MTI1	This study

Streptomyces lividans		
SBT5	*S. lividans* TK24 Δ*act*Δ*redKL* Δ*cdaPS3-SLI3600*::*afsRS*$_{cla}$	Bai et al., 2015
SBT5/pLC01	SBT5 containing the plasmid pLC01	This study
SBT5/Tn315	SBT5 containing the plasmid Tn315	This study
SBT5/pMTI	SBT5 containing the plasmid pMTI	This study
SBT5/pLC01-*idgS*	SBT5 containing the plasmid pLC01-*idgS*	This study
SBT5/Tn315-*idgS*	SBT5 containing the plasmid Tn315-*idgS*	This study
SBT5/pMTI-*idgS*	SBT5 containing the plasmid pMTI-*idgS*	This study
SBT5/pCAP01	SBT5 containing the plasmid pCAP01	This study
SBT5/pCAP-UK	SBT5 containing one-copy of the plasmid pCAP-UK	This study
RedStrep1.7	*S. lividans* TK24 Δ*act* Δ*red* Δ*cda* Δ*cpk* Δ*mel*	Novakova et al., 2018
RedStrep1.7/pCAP01	RedStrep1.7 containing the plasmid pCAP01	This study
RedStrep1.7/pCAP-UK	RedStrep1.7 containing one-copy of the plasmid pCAP-UK	This study
Streptomyces coelicolor		
M1152	*S. coelicolor* M145 Δ*act* Δ*red* Δ*cpk* Δ*cda* *rpoB*(C1298T)	Gomez-Escribano and Bibb, 2010
M1152/pLC01	M1152 containing the plasmid pLC01	This study
M1152/Tn315	M1152 containing the plasmid Tn315	This study
M1152/pMTI	M1152 containing the plasmid pMTI	This study
M1152/pLC01-*idgS*	M1152 containing the plasmid pLC01-*idgS*	This study
M1152/Tn315-*idgS*	M1152 containing the plasmid Tn315-*idgS*	This study
M1152/pMTI-*idgS*	M1152 containing the plasmid pMTI-*idgS*	This study
Streptomyces. huiliensis		
GDMCC 4.215	A novel UK-2-producing actinobacterial strain	GDMCC
Streptomyces atratus		
SCSIO ZH16NSEP-Δligase (ZH16)	An ARTP engineered strain from *S. atratus* SCSIO ZH16NS	Sun et al., 2019
ZH16/pCAP01	ZH16 containing the plasmid pCAP01	This study
ZH16/pCAP-UK	ZH16 containing the plasmid pCAP-UK	This study
Streptomyces venezuelae		
ATCC 10712	A model actinobacterial strain producing chloramphenicol	ATCC
ATCC 10712/pLC01	ATCC 10712 containing the plasmid pLC01	This study
ATCC 10712/pMTI	ATCC 10712 containing the plasmid pMTI	This study
ATCC 10712/pMTI-*sp44*	ATCC 10712 containing the plasmid pMTI-*sp44*	This study
ATCC 10712/pMTI-*stnYp*	ATCC 10712 containing the plasmid pMTI-*stnYp*	This study
Streptomyces peucetius		
ATCC 27952	A model actinobacterial strain producing epirubicin	ATCC
ATCC 27952/pLC01	ATCC 27952 containing the plasmid pLC01	This study
ATCC 27952/pMTI	ATCC 27952 containing the plasmid pMTI	This study
ATCC 27952/pMTI-*sp44*	ATCC 27952 containing the plasmid pMTI-*sp44*	This study
ATCC 27952/pMTI-*stnYp*	ATCC 27952 containing the plasmid pMTI-*stnYp*	This study
Streptomyces avermitilis		
NRRL 8165	A model actinobacterial strain producing avermectin	NRRL
NRRL 8165/pLC01	NRRL 8165 containing the plasmid pLC01	This study
NRRL 8165/pMTI	NRRL 8165 containing the plasmid pMTI	This study
Saccharopolyspora spinosa		

301	A non-model actinobacterial strain producing the broad-spectrum macrolide spinetoram (spinosyns J/K)	Wang et al., 2024
301/pSI01	*S. spinosa* 301 containing the plasmid pSI01	This study
301/pMTI	*S. spinosa* 301 containing the plasmid pMTI	This study
301/pMTI-*sp44*	*S. spinosa* 301containing the plasmid pMTI-*sp44*	This study
301/pMTI-*stnYp*	*S. spinosa* 301 containing the plasmid pMTI-*stnYp*	This study
Burkholderia gladioli		
ATCC 10248	A model *Burkholderia* strain	Johnson et al., 2015
10248/pMTI	10248 containing the plasmid pMTI	This study
10248/pMTI-*tipAp*	10248 containing the plasmid pMTI-*tipAp*	This study
10248/pMTI-*Rhap*	10248 containing the plasmid pMTI-*Rhap*	This study
10248/pMTI-*sp44*	10248 containing the plasmid pMTI-*sp44*	This study
10248/pMTI-*stnYp*	10248 containing the plasmid pMTI-*stnYp*	This study
10248/pHZ-*stnYp*-FR-MTI1-*tipAp*	10248 containing the plasmid pHZ-*stnYp*-FR-MTI1-*tipAp*	This study
***Chromobacterium* sp. Beijing**		
Beijing	A newly identified *Chromobacterium* strain producing FK228	This study
Beijing/pMTI	Beijing containing the plasmid pMTI	This study
Beijing/pMTI-*tipAp*	Beijing containing the plasmid pMTI-*tipAp*	This study
Beijing/pMTI-*Rhap*	Beijing containing the plasmid pMTI-*Rhap*	This study
Beijing/pMTI-*sp44*	Beijing containing the plasmid pMTI-*sp44*	This study
Beijing/pMTI-*stnYp*	Beijing containing the plasmid pMTI-*stnYp*	This study

Table S2. Plasmids used in this study

Plasmids	Relevant features	Source
pLC01	PhiC31 *int*/*attP*, *acc(3)IV*, pUC19 *ori*, *oriT* RK2 and *kasOp** promoter	Li et al., 2019
Tn315	Transposition plasmid based on the IS204 transposon	Our lab
pHZ	pHZAUFXJ BAC vector, *Φ*C31 *int/attP*, *oriT* RK2, *acc(3)IV*, plasmid for the construction of BAC library, *parA*, *parB*, *parC*, *oriV*, *redF*, *repE*, *lacZα*	Our lab
pSET-*sp44*-*indC*	A derivative of pSET-*indC* with the gene *indC* under the control of the strong promoter *sp44*	Guo et al., 2023
pSET-*stnYp*-*indC*	A derivative of pSET-*indC* with the gene *indC* under the control of the strong promoter *stnYp*	Guo et al., 2023
pKCCas9dO	*acc(3)IV*, pSG5, *tipA-Scocas9*, j23119, *actII-orf4* guide-RNA, homologous region flanking *act-orf4*	Huang et al., 2015
pBBR1-Rha-Km-Redγ-BAS	pBBR1 with the Redγ-BAS system under the control of the inducible promoter *Rhap*	Bai et al., 2023
pUC57-MTI_1737	The plasmid pUC57 with the codon-optimized gene MTI_1737	This study
pUC57-MTI_2871	The plasmid pUC57 with the codon-optimized gene MTI_2871 and the strong promoter *ermEp**	This study
pUC57-MTI_6538	The plasmid pUC57 with the codon-optimized gene MTI_6538	This study
pUC57-MTI_Cp36	The plasmid pUC57 with the codon-optimized gene MTI_Cp36	This study
pMTI_2871 (pMTI)	pLC01 with the MTI1 (MTI_2871) *int*/*attP* system under the control of the promoter *ermEp** instead of the Phi31 *int*/*attP* system	This study
pMTI-*sp44*	pMTI with the MTI1 (MTI_2871) *int*/*attP* system under the control of the promoter *sp44* instead of the promoter *ermEp**	This study
pMTI-*stnYp*	pMTI with the MTI1 (MTI_2871) *int*/*attP* system under the control of the strong promoter *stnYp* instead of the strong promoter *ermEp**	This study
pMTI_1737	pMTI with the MTI_1737 *int*/*attP* system instead of the MTI1 (MTI_2871) *int*/*attP* system	This study
pMTI_1737-*stnYp*	pMTI_1737 with the MTI_1737 *int*/*attP* system under the control of the promoter *stnYp* instead of the promoter *ermEp**	This study
pMTI_6538	pMTI with the MTI_6538 *int*/*attP* system instead of the MTI1 (MTI_2871) *int*/*attP* system	This study
pMTI_6538-*stnYp*	pMTI_6538 with the MTI_6538 *int*/*attP* system under the control of the promoter *stnYp* instead of the promoter *ermEp**	This study
pMTI_Cp36	pMTI with the MTI_Cp36 *int*/*attP* system instead of the MTI1 (MTI_2871) system	This study
pMTI_Cp36-*stnYp*	pMTI_Cp36 with the MTI_Cp36 *int*/*attP* system under the control of the promoter *stnYp* instead of the promoter *ermEp**	This study
pLC01-*idgS*	pLC01 with the reporter gene cassette *idgS*-*sfp* under the control of the promoter *kasOp**	Our lab
Tn315-*idgS*	Tn315 with the reporter gene cassette *idgS*-*sfp* under the control of the promoter *kasOp**	Our lab
pMTI-*idgS*	pMTI with the reporter gene cassette *idgS*-*sfp* under the control of the promoter *kasOp**	This study
pMTI-*stnYp*-*idgS*	pMTI-*stnYp* with the reporter gene cassette *idgS*-*sfp* under the control of the promoter *kasOp**	This study
pMTI-*tipAp*	pMTI with the MTI1 *int*/*attP* system driven by the promoter *tipAp* instead of the promoter *ermEp**	This study
pMTI-*Rhap*	pMTI with the MTI1 *int*/*attP* system driven by the promoter *Rhap* instead of the	This study

	promoter *ermEp**	
pCAP01	BGC cloning vector; ARSH4/CEN6, TRP1, pUC *ori*, PhiC31 *int*/*attP*, *aphII*, *oriT* RK2	Yamanaka et al., 2014
pCAP-UK-HA	pCAP01 with two homologous arms for cloning the entire UK-2 BGC from *S. huiliensis* GDMCC 4.215	This study
pCAP-UK	pCAP01 with the entire UK-2 BGC	This study
pCB003	pMB1*ori*, *aadA,* the sgRNA scaffold under the control of the promoter j23119	Jiang et al., 2015
pCB006	*repA101*(Ts) *aphII Pcas-cas9 ParaB-Red lacI*^{q} *Ptrc-sgRNA-pMB1*	Jiang et al., 2015
pCB003-C31	pMB1*ori*, *aadA*, the sgRNA targeting the PhiC31 gene under the control of the promoter j23119	This study
pCAP-UK-MTI1	pCAP-UK with the MTI1 *int*/*attP* & *aac(3)IV* system instead of the PhiC31 *int*/*attP* & *aphII* system	This study
pCAP-UK-BT1	pCAP-UK with the PhiBT1 *int*/*attP* & *aac(3)IV* system instead of the PhiC31 *int*/*attP* & *aphII* system	This study
pBAC-SalRefFad	The plasmid pHZ with the 106-kb multi-operon artificial salinomycin BGC	Jiang et al., 2021
pBAC-SalRefFad-MTI1	pBAC-SalRefFad with the MTI1 *int*/*attP* system instead of the PhiC31 *int*/*attP* system	This study
pSI01	pCM265 derived plasmid by removing *Bsa* I and *BamH* I sites within the Int32 integrase encoding gene via site-directed mutagenesis and adding the multiple cloning sites including *Bgl* II, *Bam*H I, *Nde* I and *Swa* I	Wang et al., 2025
pHZ-FR-3C6	The plasmid pHZ with the complete FR900359 BGC under the control of the original promoter and upstream as well as downstream regions out of this BGC	This study
pHZ-*stnYp*-FR	pHZ-FR-3C6 with the promoter *stnYp* instead of the original promoter	This study
pHZ-*stnYp*-FR-MTI1	pHZ-*stnYp*-FR with the MTI1 *int*/*attP* system instead of the PhiC31 *int*/*attP* system	This study

Table S3. Oligonucleotide sequences used in this study

Primers	Sequence (5'-3')
**Primers for the construction and identification of pMTI_2871 (pMTI)**	
pLC-skeleton-HindIII-fw	cgcaattgatttaaattgaagccgccgaagccccgAAGCTT
pLC-skeleton-EcoRI-rev	actactcccgaaaaccgcttctgacctggGAATTCgtgaagccc
ID-MTI-fw	cgccctcctacatcgaagct
ID-MTI-rev	aatcgccctgggtgggtt
**Primers for the construction of pMTI-*sp44* and pMTI-*stnYp***	
MTI-fw	catatgaacaaccgcatcga
pMTI-skeleton-rev	gaattcgtgaagccccggggcat
skeleton-*sp44*-fw	ccggggcttcacgaattctgttcacattcgaacggt
sp44-MTI-rev	gatgcggttgttcatatgcgttgacgatcccccgagt
skeleton-*stnYp*-fw	ccggggcttcacgaattcgcatccggtccgcgaaggat
stnYp-MTI-rev	tcgatgcggttgttcatatgcgttgacgatcccccgagt
**Primers for the construction and identification of other MTI-series plasmids**	
pMTI-skeleton-fw	cgcaattgatttaaattgaa
*ermEp**-rev	atgtggatcctaccaaccggcacgat
stnYp-rev	tgcgttgacgatcccccgagt
*ermEp**-1737-fw	cggttggtaggatccacatatgaagcagcagatctaca
stnYp-1737-fw	tcgggggatcgtcaacgcatatgaagcagcagatctaca
1737-rev	cttcaatttaaatcaattgcgggctgtttcttaatatctt
*ermEp**-6538-fw	ccggttggtaggatccacatatgctccagaccgacaagat
stnYp -6538-fw	tcgggggatcgtcaacgcatatgctccagaccgacaagat
6538-rev	ttcaatttaaatcaattgcgggtgttttcaaaagtatcgtt
*ermEp**-Cp36-fw	ccggttggtaggatccacatatgaagcagctcaacatcca
stnYp-Cp36-fw	tcgggggatcgtcaacgcatatgaagcagctcaacatcca
Cp36-rev	ttcaatttaaatcaattgcgcttaaccgcttttgaatgt
**Primers for the construction of pMTI-*idgS* and pMTI-*stnYp-idgS***	
XbaI-*idgS*-fw	ccttaattaaggatccTCTAGAtgactcttcaggagaccagcgt
idgS-ter-rev	tgggctgcaggtcgactctagtaagtgtacacatcccctt
**Primers for the construction of pMTI-*tipAp***	
MTI-*tipAp*-fw	ccggggcttcacgaattccaaccgagcgttctgaacaaat
tipAp-MTI-rev	gatgcggttgttcatatgcgctcccttctctgacgccgt
**Primers for the construction of pMTI-*Rhap***	
MTI-*Rhap*-fw	gccccggggcttcacgaattcattaatctttctgcgaatt
Rhap-MTI-rev	tcgatgcggttgttcatatgttcattacgaccagtcta
**Primers for the direct cloning and identification of pCAP-UK**	
pCAP-up-fw	ctgcgccgatggtttctacaaagatcgACTAGTtcacgcgggcgttccttcgg
pCAP-up-rev	GTTTAAACgcagttgtcgaggacgaggagc
pCAP-down-fw	ggcgggcgctcctcgtcctcgacaactgcGTTTAAACaagcggatgtggtgctggtcg
pCAP-down-rev	acccctatttgtttatttttctaaatacaGGTACCcaccatgttcggcgtgctgtt
UK-up-sgRNA-fw	gactgacactgataatacgactcactataggacggcgcgaacggcgcgggttttagagctagaaatagc
UK-down-sgRNA-fw	gactgacactgataatacgactcactataggtgccgagcagggtgcccggttttagagctagaaatagc
UK-sgRNA-rev	ctcaaaaaaagcaccgactcgg
ID-pCAP-UK-1-fw	cgttccaccgctcgaacctg
ID-pCAP-UK-1-rev	tggagccgttcgtgggcgta
ID-pCAP-UK-2-fw	gccaggtacggatcggtggtc

ID-pCAP-UK-2-rev	acgcccaatggctcgacacc
ID-pCAP-UK-3-fw	gacggttccgcactggttcctg
ID-pCAP-UK-3-rev	agctcgtcaccctcgctcatcc
**Primers for the construction of pCB003-C31**	
pCB003-skeleton-fw	aagcttagatctattaccct
pCB003-skeleton-rev	ACTAGTattatacctaggact
PhiC31-sgRNA-fw	cctaggtataatACTAGTgggcttcggcggcttcaagttgttttagagctagaaatag
sgRNA-rev	agggtaatagatctaagcttctcaaaaaaagcaccgact
**Primers for the construction and identification of pCAP-UK-MTI1**	
acc(3)IV-MTI1-fw	gcgtcgcttggtcggtcatttcgaaccccagagtcccgcaatctacggggtctgacgct
acc(3)IV-MTI1-rev	cttctcgacagacgtagatcaggcttcccgggtgtctcgtctgcgtgtagttccttgta
ID-MTI1-fw	atcctgttccacggcaagga
ID-MTI1-rev	atctgctggtagacgacggc
**Primers for the construction and identification of pCAP-UK-BT1**	
acc(3)IV-BT1-fw	gcgtcgcttggtcggtcatttcgaaccccagagtcccgccctttgatctACTAGTagct
acc(3)IV-BT1-rev	cttctcgacagacgtagatcaggcttcccgggtgtctcgctacagcgccgcaagctc
ID-BT1-fw	ctgaagggtgcgaaagacg
ID-BT1-rev	cgctaggtcgtgctgaagg
**Primers for the construction and identification of pBAC-SalRefFad-MTI1**	
Sal-MTI1-up-fw	ctcgtgttccaactgagtgtataga
Sal-MTI1-up-rev	gtcgacagcgacacacttgc
Sal-MTI1-down-fw	actactcccgaaaaccgctt
Sal-MTI1-down-rev	gtttcatcagccatccgctt
Sal-MTI1-fw	taaccgggctgcatccgatgcaagtgtgtcgctgtcgactctgcgtgtagttccttgta
Sal-MTI1-rev	tcacgttttcccaggtcagaagcggttttcgggagtagtggatccctctagtatgcatg
ID-MTI1-up-fw	ctcgtgttccaactgagtgtataga
ID-MTI1-up-rev	atcctgttccacggcaagga
ID-MTI1-down-fw	agcttgtagaccacgacctt
ID-MTI1-down-rev	gtttcatcagccatccgctt
ID-salQP-fw	tcgccaggaggatccaggaca
ID-salQP-rev	ctcggtggtcggcatgca
ID-salCX-fw	agccggacaccggtgacct
ID-salCX-rev	agtcgagcctgtgccggat
ID-salB-fw	gacgtcgacggcgttgat
ID-salB-rev	atctcacgagcattttccgca
ID-salFD-fw	tgacgcggatggcctcca
ID-salFD-rev	tacgaggaccgcgagatctt
ID-salAII-fw	accacccggacccggaca
ID-salAII-rev	tcgaccacgtcgacgtcgcg
ID-salAV-fw	agcggctcgtcctggagg
ID-salAV-rev	cttcggcaggacgccgtg
**Primers for the construction and identification of pCB003-FR-*stnYp***	
pCB003-skeleton-fw	aagcttagatctattaccct
pCB003-skeleton-rev	actagtattatacctaggactgagctagct
FR-Rep-up-fw	ggacccgttggcaggaagca
FR-Rep-up-rev	ggatccccgggtaccgagct
FR-Rep-down-fw	atgaaaaacagtgaatcgcc

FR-Rep-down-rev	gtaggataacagggtaatagatctAAGCTTctcggatgcgtacaaatcga
FR-upstream-sgRNA-fw	agctagctcagtcctaggtataatACTAGTtctcgagcatggtctcggccgttttagagctagaaatagc
FR-upstream-sgRNA-rev	cttccggaagtgcttcctgccaacgggtccctcaaaaaaagcaccgactc
stnYp-fw	ggcgaattcgagctcggtacccggggatccgcatccggtccgcgaaggat
stnYp-rev	atgatggattggcgattcactgtttttcatcgttgacgatcccccgagtg
ID-pCB003-fw	ccgctcgccgcagccgaacga
ID-pCB003-rev	cgccaactacctctgatagt
**Primers for identification of pHZ-FR-3C6, pHZ-*stnYp*-FR and pHZ-*stnYp*-FR-MTI1-*tipAp***	
ID-FR-3C6-up-fw	ttgaaaatggcgcatcaggc
ID-FR-3C6-up-rev	acacaccacattgccaaacg
ID-FR-3C6-down-fw	taccggtaccaacgctttcc
ID-FR-3C6-down-rev	cgccagctggttagactgaa
ID-FR-frsA-fw	cgcagcattcagccgaatgg
ID-FR-frsA-rev	gcttgacagcggtgacagac
ID-FR-frsBC-fw	ctggttctgatcaatgacga
ID-FR-frsBC-rev	gcctgccatggatcatcgct
ID-FR-frsD-fw	ggctggcgcaacagctgatg
ID-FR-frsD-rev	gcaggcgtcttccggaatca
ID-FR-frsE-fw	ctatgcgcagcgccgattgt
ID-FR-frsE-rev	cgataggccagttccagatc
ID-FR-frsF-fw	cgttgcagccgctgtcgggcta
ID-FR-frsF-rev	gattcatacaaggcacctag
ID-FR-frsG-fw	gctgttcgacatgccgacct
ID-FR-frsG-rev	gcgatcagactctccggatc
ID-FR-frsH-fw	ggctttgctgcaattgcgag
ID-FR-frsH-rev	cactcctcgacgatgcgcca
ID-FR-*stnYp*-fw	aacagggtaatcgtcagcg
ID-FR-*stnYp*-rev	agaccatcgtgacggaag
ID-FR-*tipAp*-fw	agcgattccagacgtcccga
ID-FR-*tipAp*-rev	cgaagacgatgcagatgttc
ID-FR-MTI1-fw	agctgacagtcgaggcgatc
ID-FR-MTI1-rev	cgctgacgattaccctgtt

Note: The capital letters represent the restriction enzyme sites. The blue letters represent the sequences of overlaps. The red letters represent the guide sequence of sgRNA.

Table S4. Comparison of integrase-based MNGE and other methods for multi-copy genomic integration of large DNA fragments

Method	Design principle	Host range	Size of integrated DNA fragment	Copies of integrated DNA fragment	DNA Integration efficiency	Copy control accuracy	Reference
MNGE	One multi-targeting integrase (i.e., MTI1 and Cp36) & Multiple native *attB* sites	Bacteria and human cell	up to 106 kb	One to three copies	7-100%	Low	This study
MSGE	One site-specific integrase (i.e., PhiC31 and PhiBT1) & Multiple artificial *attB* sites	Bacteria	up to 67 kb	One to four copies	10-100%	High	Li et al., 2017
aMSGE	Multiple site-specific integrases (i.e., PhiC31, PhiBT1, R4 and SV1) & Multiple native *attB* sites	Actinobacteria	up to 72 kb	One to four copies	30-100%	High	Li et al., 2019
PASTE	One nCas9-RT-serine integrase (i.e., Bxb1 and PhiBT1) & One artificial *attB* site	Human cell	up to 36 kb	One copy	50–60%	High	Yarnall et al., 2021

Protein ID: MTI_1737

Protein seq：
MKQQIYNTALYLRLSRDDELQGESSSITTQRSMLRLYAKEHHLNVIDEYIDDGWSGTNFDRPSFQRMIEDIEAGKINCVVTKDLSRLGRNYIMTGQYTELYFPSHNVRYIAIDDGVDS
EKGESEIAPFKNIINEWVARDTSRKVKSAFKTKFAEGAHYGAYAPLGYKKHPDIKGKLLVDDETKWIIEKIFSLAYQGYGSAKITKQLRAEKVPTASWLNFTRYGTFAHIFEGKPESK
RYEWTIAHVKAILKSEVYIGNSVHNMQSTVSFKSKKKVRKPESEWFRVENTHEPIIDKEVFYRVQEQIKSRRRQTKEKATPIFAGLVKCADCGWSMRFGTNKTNKTPYSYYACSYY
GQFGKGNCSMHYIRYDVLYQAVLERLQYWAKAVQQDEEKVLNKIQKAGNAERIREKKKKASTLKKAENRQNEIDRLFAKMYEDRACEKITERNFVMLSSKYQKEQIELEQQITSL
REELSKMEQDMIGAEKWIELIKEYSVPKELTAPLLNAMIEKILIHEATTNEDNERIQEIEIYYRFIGKVE

Codon-optimized gene seq:
ATGAAGCAGCAGATCTACAACACGGCGCTCTACCTGCGTCTGTCGCGCGACGACGAGCTGCAGGGAGAGTCCTCCTCGATCACCACGCAACGCTCGATGCTGCGGCTGTAT
GCCAAAGAGCACCACCTGAACGTCATCGACGAATACATCGATGACGGCTGGAGCGGTACGAACTTCGACCGGCCCAGCTTCCAGCGGATGATCGAGGACATCGAGGCGGG
AAAGATCAACTGCGTCGTCACGAAGGACCTGAGTCGGCTGGGCCGGAACTACATCATGACCGGCCAGTACACGGAACTGTACTTCCCCTCCCACAACGTGCGCTACATCGC
CATCGACGACGGGGTTGACAGCGAGAAGGGCGAGAGCGAGATAGCCCCCTTCAAGAATATCATCAACGAGTGGGTGGCCCGGGACACCAGCCGCAAGGTCAAGAGCGCG
TTCAAGACGAAGTTCGCCGAGGGCGCCCACTACGGCGCCTACGCGCCGCTTGGCTACAAGAAGCACCCCGACATCAAGGGCAAGCTGCTGGTCGACGACGAGACGAAGTG
GATCATCGAGAAGATCTTCTCCCTCGCCTACCAGGGCTACGGGTCCGCCAAGATCACCAAGCAGCTCCGGGCCGAGAAGGTGCCCACCGCATCCTGGCTCAACTTCACCCG
GTACGGCACCTTCGCGCACATCTTCGAGGGCAAGCCGGAGTCCAAGCGGTACGAATGGACCATCGCGCACGTCAAGGCGATCCTCAAGTCCGAGGTCTACATTGGCAACA
GCGTGCACAACATGCAGTCGACGGTCTCGTTCAAGTCCAAAAAGAAAGTGCGCAAGCCCGAGTCGGAGTGGTTCCGCGTCGAGAACACCCATGAGCCGATCATCGACAAG
GAAGTGTTCTACAGGGTGCAGGAGCAGATCAAGTCCCGCCGCCGGCAGACCAAGGAGAAGGCTACTCCGATCTTCGCCGGGCTGGTGAAGTGCGCCGACTGCGGCTGGTC
CATGCGCTTCGGCACCAACAAGACCAACAAGACCCCGTACTCGTACTACGCCTGCTCGTACTACGGGCAGTTCGGCAAGGGTAACTGTTCCATGCACTACATCCGGTACGA
CGTGCTCTACCAGGCGGTCCTGGAGAGACTCCAGTACTGGGCGAAGGCCGTCCAGCAGGACGAGGAGAAGGTGCTCAACAAGATCCAGAAGGCCGGTAACGCCGAGCGC
ATCCGAGAGAAGAAGAAGAAGGCCTCCACCCTGAAGAAGGCGGAGAACCGCCAGAACGAGATCGACCGCCTCTTCGCGAAGATGTACGAGGACCGTGCGTGCGAGAAGA
TCACCGAGCGCAACTTCGTGATGCTGTCCAGCAAGTACCAGAAGGAACAGATCGAGCTGGAGCAGCAGATCACGTCCCTGCGCGAGGAGCTGTCGAAGATGGAGCAGGAC
ATGATCGGCGCCGAGAAGTGGATCGAACTGATCAAGGAGTACTCGGTCCCGAAGGAACTCACCGCCCCGCTGCTCAACGCGATGATCGAGAAGATCCTCATCCACGAGGC
CACCACCAACGAGGACAACGAACGGATACAGGAGATCGAAATCTACTACCGGTTCATCGGGAAGGTCGAGTAA

***attP* site :** TCAACAAGAGTAATATTTTTAACTAAGGGAAACCGGTGTTTCCCAAACAAAAAGATATTAAGAAACAGCC

Figure S1. Protein sequence, codon-optimized gene sequence and *attP* site of MTI_1737

Protein ID: MTI_2871 (MTI1)

Protein seq:
MNNRIDAIYARQSVDKKDSISIESQIEFCKYELKGGNCKEYTDKGYSGKNTDRPKFQELVRDIKRGLIAKVVVYKLDRISRSILDFANMMELFQQYNVEFVSSTEKFDTSTPMGRAM
LNICIVFAQLERETIQKRVTDAYYSRSQRGFKMGGKAPYGFHTEPIKMDGINTKKLVVNPEEAANIRLMFEMYAQPTTSYGDITRYFAEQGILFHGKELIRPTLAQMLRNPVYVQADL
DVYEFFKSQGTVIVNDVADFTGMNGCYLYQGRDVKASKKNDLKDQMLVLAPHEGIVPSDTWLTCRKKLMNNMKIQSARKATHTWLAGKIKCGNCGYALMSIYNPSGKQYLRCT
KRLDNKSCPGCGKIITSELEAVVYQQMVKKLASYKTLTGKKKAAKANPKITALQVELAHVDSEIEKLVDSLTGANNVLFSYVNVKIAELDGRKQELLARIAELTVEAISPEQVSQISG
YLDTWENVSFDDKRRVVDLMITTIAATSDSLNITWKI

Codon-optimized gene seq:
ATGAACAACCGCATCGACGCCATCTACGCGCGGCAGTCGGTGGACAAGAAGGACTCCATCTCCATCGAGTCCCAGATCGAGTTCTGCAAGTACGAACTCAAGGGCGGCAA
CTGCAAGGAGTACACCGACAAGGGGTACAGCGGAAAGAACACCGACCGGCCGAAGTTCCAGGAGCTGGTCCGGGACATCAAGCGCGGTCTCATTGCGAAGGTCGTGGTCT
ACAAGCTGGACCGAATCTCGCGCTCGATCCTGGACTTCGCGAACATGATGGAGTTATTCCAGCAGTACAACGTCGAGTTCGTCAGCAGTACGGAGAAGTTCGACACCTCTA
CCCCGATGGGCCGGGCGATGCTGAACATCTGCATCGTCTTCGCCCAGCTGGAGCGCGAGACCATCCAGAAGCGCGTGACCGACGCGTACTACTCCCGGTCACAGCGGGGCT
TCAAGATGGGCGGGAAGGCACCCTACGGCTTCCACACCGAGCCGATCAAGATGGACGGCATCAACACGAAGAAGCTGGTGGTCAACCCCGAGGAGGCCGCCAACATCCGC
CTGATGTTCGAGATGTACGCCCAGCCCACCACCTCGTACGGCGACATCACGCGGTACTTCGCCGAGCAGGGCATCCTGTTCCACGGCAAGGAGCTGATCCGGCCCACCCTG
GCGCAGATGCTGCGCAACCCCGTATACGTGCAGGCCGACCTGGACGTGTACGAGTTCTTCAAGTCGCAGGGCACGGTGATCGTGAACGACGTGGCCGACTTCACCGGCATG
AACGGCTGCTACCTGTACCAAGGTAGGGACGTCAAGGCCAGCAAGAAGAACGACCTGAAGGACCAGATGCTGGTCCTCGCCCCGCACGAGGGCATCGTTCCGAGCGATAC
TTGGCTCACCTGCCGGAAGAAGCTGATGAACAACATGAAGATCCAGTCCGCGCGCAAGGCCACCCACACCTGGCTGGCCGGCAAGATCAAGTGCGGGAACTGCGGCTACG
CCCTGATGAGCATCTACAACCCGTCGGGGAAGCAGTACCTGCGCTGCACCAAGCGGCTGGACAACAAGTCCTGCCCCGGCTGTGGGAAGATAATCACCTCCGAATTGGAA
GCCGTCGTCTACCAGCAGATGGTGAAGAAACTCGCCTCCTACAAGACGCTGACCGGGAAGAAGAAGGCGGCCAAGGCCAATCCGAAGATCACCGCCCTGCAGGTGGAGCT
CGCGCACGTCGACTCGGAGATCGAGAAGCTCGTCGACTCCCTCACGGGTGCCAACAACGTGCTGTTCTCGTACGTCAACGTGAAGATCGCTGAGCTCGACGGCCGCAAGCA
GGAGCTGCTCGCCCGCATCGCGGAGCTGACAGTCGAGGCGATCTCCCCGGAGCAGGTGAGCCAGATCTCCGGATATCTCGACACCTGGGAGAACGTCTCCTTCGACGACAA
ACGTCGCGTCGTAGACCTCATGATCACCACGATCGCGGCGACGAGCGACAGCCTCAACATCACGTGGAAGATCTGA

***attP* site:** ACCCTGTGTAGTCCCTTGTAAACTGTACTTTAGGTCAGTTTACAAGGAACTACACGCAGA

Figure S2. Protein sequence, codon-optimized gene sequence and *attP* site of MTI_2871 (MTI1)

Protein ID: MTI_6538

Protein seq:
MLQTDKITALYCRLSQEDMQAGESESIQNQKLILQKYADEHHFFNTRFFVDDGFSGVSFEREGLQAMLHEVEAGNVATVITKDLSRLGRNYLKTGELIEIVFPEYEVRYIAINDGVDT
AREDNEFTPLRNWFNEFYARDTSKKIRAVKQAKAQKGERVNGEAPYGYLIDPDNRNHLIPDPETAHVVKQIFAMYVRGDRMCEIQNWLRDNEILTVGELRYRRTGSKRHPRPQLNA
WYNWPDKTLYDILTRKEYLGHTITGKTYKVSYKSKKTKKNPEEKRYFFPNTHEPLIDEETFELAQKRIATRQRPTKVDEIDLFSGLLFCGDCGYKMYAVRGAGTLERKHAYTCGNY
RNRARNDMLCTTHYIRKSVLKELVLADLQRVTSYVKEHEQEFIETANECSAKAVQKTLTQQRKELDKAQNRINELNILFRKLYEDNALGKLSDEQFAFLTSGYDEEKKTLTRRIAEL
SQEIDNATERSADVKRFVALVRRYTAIEELTYENVHEFIDRILIHELDKETNTRKIEIFYSFVGRVDTGDKPTESISYFRQIGADVKSYAI

Codon-optimized gene seq:
ATGCTCCAGACCGACAAGATCACCGCGCTGTACTGCCGGCTGTCCCAGGAGGACATGCAGGCGGGTGAGAGCGAGTCCATCCAGAACCAGAAGCTCATCCTCCAGAAGTAC
GCCGACGAACACCACTTCTTCAACACCCGCTTCTTCGTGGACGACGGCTTCAGCGGGGTCTCCTTCGAGCGGGAGGGCCTGCAGGCCATGCTGCATGAGGTGGAGGCGGGC
AACGTCGCGACGGTGATCACCAAGGACCTCTCCCGCCTCGGCCGCAACTACCTCAAGACCGGGGAGCTGATAGAGATCGTCTTCCCGGAGTACGAGGTCCGTTACATCGCCA
TCAACGACGGGGTCGACACCGCGCGCGAGGATAACGAGTTCACCCCCCTGCGCAACTGGTTCAACGAGTTCTACGCCCGGGACACCAGCAAGAAGATCCGGGCCGTGAAGC
AGGCCAAAGCGCAGAAGGGAGAACGGGTGAACGGCGAGGCCCCGTACGGCTACCTCATCGATCCGGACAATCGGAACCACCTGATCCCGGACCCCGAGACCGCCCACGTC
GTCAAGCAGATCTTCGCGATGTACGTCCGCGGAGACCGGATGTGCGAGATCCAGAACTGGCTGCGCGACAACGAGATCCTGACGGTCGGCGAGCTTCGCTACAGGCGCACC
GGCAGCAAGCGGCACCCGCGGCCCCAGCTCAACGCGTGGTACAACTGGCCCGACAAGACGCTCTACGACATCCTGACCCGGAAGGAGTACCTGGGCCACACCATCACGGGC
AAGACCTACAAGGTGTCGTACAAGTCGAAGAAGACCAAGAAGAACCCGGAAGAGAAGCGGTACTTCTTCCCCAACACCCACGAGCCGCTGATCGACGAGGAGACCTTCGA
GCTGGCCCAGAAGCGTATCGCCACCCGCCAGCGGCCGACCAAGGTCGACGAGATCGACCTCTTCTCCGGCCTGCTGTTCTGCGGTGACTGCGGGTACAAGATGTACGCGGTG
AGAGGCGCCGGCACCCTGGAGCGCAAGCACGCCTACACCTGTGGGAACTACCGGAACCGCGCCCGCAACGACATGCTGTGCACCACGCACTACATCCGCAAGTCGGTGCTG
AAGGAACTGGTCCTCGCCGACCTGCAGCGGGTGACGTCGTACGTAAAGGAGCACGAACAGGAGTTCATCGAAACAGCCAACGAGTGCAGCGCGAAGGCGGTCCAGAAGAC
TCTCACCCAGCAGCGCAAAGAGCTCGACAAGGCCCAGAACCGGATCAACGAACTGAACATCCTGTTCCGCAAGTTGTATGAGGACAACGCCCTCGGCAAGCTCTCCGACGA
GCAGTTCGCCTTCCTCACGTCAGGCTACGACGAGGAGAAGAAAACGCTGACGCGACGGATCGCGGAGCTCAGCCAAGAGATCGACAACGCCACGGAGCGTTCCGCCGACG
TCAAGCGCTTCGTTGCATTAGTGAGGCGCTACACCGCGATCGAGGAGCTGACCTACGAAAATGTGCACGAGTTCATCGACCGTATCCTGATCCACGAGCTGGACAAGGAGAC
CAACACGCGGAAGATCGAGATCTTCTACTCGTTCGTCGGGCGCGTCGACACGGGCGACAAGCCCACCGAGTCGATCTCCTACTTCCGGCAGATCGGCGCGGACGTGAAGTCC
TACGCTATCTAA

***attP* site:** TCTGCAAAAAAGGTATCGTTATCTTACCTCAACAAAAGCTGACGTTAGATAACGATACTTTTGAAAACAC

Figure S3. Protein sequence, codon-optimized gene sequence and *attP* site of MTI_6538

Protein ID: MTI_Cp36

Protein seq：
MKQLNIQSSSKITALYCRLSRDDELQGTSNSILNQKMMLEKYARDNNFTNLEFFIDDGYSGTNFNRPDWSRLQSLIDEGKIGCIIVKDMSRLGRDYLRVGYYTDIVFPEADIRFIAINN
GIDSNESTENDLTPFINIINEFYAKDTSKKIRAVFKAKGESGKPLATIPPYGYLKDKEDKYKWVIDEEASKVVKKIFQLCVQGYGPSQIASELIKEGIPTPTEHFDKLGINVSSPLSEIKGN
WQPKTISLILEKMEYLGHTVNFKTYKKSYKSKKKLENPKEKWQIFENTHEAIIDQETFDIVQRIRQGRRVRNNLGEMPALSGMLYCADCGAKLYQVRGKGWEHEKEYFVCASYRK
HKGLCTSHQIKNVQVEELLLHELKKITEYARQYEDDFVKLVQSKTQNELNKSLKESKKDLVHVKERINKLDTIIQRLYEDMVEGKLSEDRFQKLSSNYETEQCELEKKATMLEKIIH
DTEQTTLNTTAFLKQVREHTTINKLTPEIIRMFVDKIIVEKPEKIEGTRTKKQTIWIYWNYIGILDIEKTAE

Codon-optimized gene seq:
ATGAAGCAGCTCAACATCCAGAGCTCTTCGAAGATCACCGCGCTCTACTGCCGCCTCTCCCGCGACGATGAACTGCAGGGTACCAGCAACTCCATACTCAACCAGAAGATG
ATGCTGGAGAAGTACGCGCGTGACAACAACTTCACCAACTTAGAGTTCTTCATCGACGACGGCTACAGTGGCACGAACTTCAACCGCCCCGACTGGTCCCGGCTGCAGTCG
CTGATCGACGAAGGAAAGATCGGCTGCATCATCGTGAAGGACATGTCGCGGCTCGGCCGGGACTACCTGCGCGTCGGTTACTACACCGACATCGTCTTCCCCGAGGCGGAC
ATCAGGTTCATCGCGATCAACAACGGCATCGATTCGAACGAATCCACTGAGAATGACCTGACCCCCTTCATCAACATCATCAACGAGTTCTACGCCAAGGACACCTCGAAG
AAGATCCGGGCGGTGTTCAAGGCGAAGGGCGAGTCCGGTAAGCCCCTCGCCACGATCCCGCCGTACGGGTACCTCAAGGACAAGGAGGACAAGTACAAGTGGGTGATCGA
CGAGGAGGCCTCCAAGGTCGTCAAGAAGATCTTCCAGCTGTGCGTGCAAGGCTACGGCCCGTCCCAGATCGCCTCGGAGCTGATCAAGGAGGGCATCCCCACCCCGACCG
AGCACTTCGACAAGCTGGGGATCAACGTCTCCAGCCCGCTGAGCGAGATCAAGGGCAACTGGCAGCCCAAGACCATCAGCCTCATCCTGGAGAAGATGGAGTATCTGGGC
CATACGGTCAACTTCAAGACCTACAAGAAGTCCTACAAGTCCAAGAAGAAGCTCGAGAACCCGAAGGAGAAGTGGCAGATCTTCGAGAACACCCACGAGGCCATCATCGA
CCAGGAGACCTTCGACATCGTCCAGCGGATCCGCCAGGGACGGCGGGTCCGCAACAACCTGGGGGAGATGCCGGCCCTGTCCGGCATGCTCTACTGCGCGGACTGCGGCG
CCAAGCTCTACCAGGTGCGGGGGAAGGGCTGGGAGCACGAAAAGGAGTACTTCGTCTGCGCCTCGTACCGCAAGCACAAGGGCCTGTGTACCAGCCACCAGATCAAGAAC
GTGCAGGTTGAAGAACTGCTGCTGCACGAGCTCAAGAAGATCACGGAGTACGCCCGTCAGTACGAGGACGACTTCGTGAAGCTCGTGCAGTCGAAGACCCAGAACGAGCT
GAACAAGAGCCTCAAGGAGAGCAAGAAGGACCTGGTCCACGTCAAGGAGCGCATCAACAAGCTCGACACAATTATCCAGAGACTGTACGAGGACATGGTCGAGGGCAAA
CTGTCCGAGGACCGGTTCCAGAAGCTGTCGTCCAACTACGAAACGGAGCAGTGCGAGCTGGAGAAGAAGGCCACCATGCTGGAGAAAATAATCCACGACACCGAGCAGAC
CACGCTCAACACCACGGCCTTCCTGAAGCAGGTGCGCGAGCACACCACCATCAACAAGCTCACGCCCGAGATCATCCGCATGTTCGTCGACAAGATCATCGTGGAGAAGCC
GGAGAAAATCGAGGGGACCCGCACCAAGAAGCAGACGATCTGGATCTACTGGAACTACATCGGCATCCTGGACATCGAGAAGACGGCATAA

***attP* site :** ACGATTATGCCGTTAATTTTAAAAGGAATAAATCCCTATTGGAAGTCACCTTTTGGAGCGTTCAATTAGGGATTAACTAAGACGGACATTCAAAAGCGGTTAAG

Figure S4. Protein sequence, codon-optimized gene sequence and *attP* site of MTI_Cp36

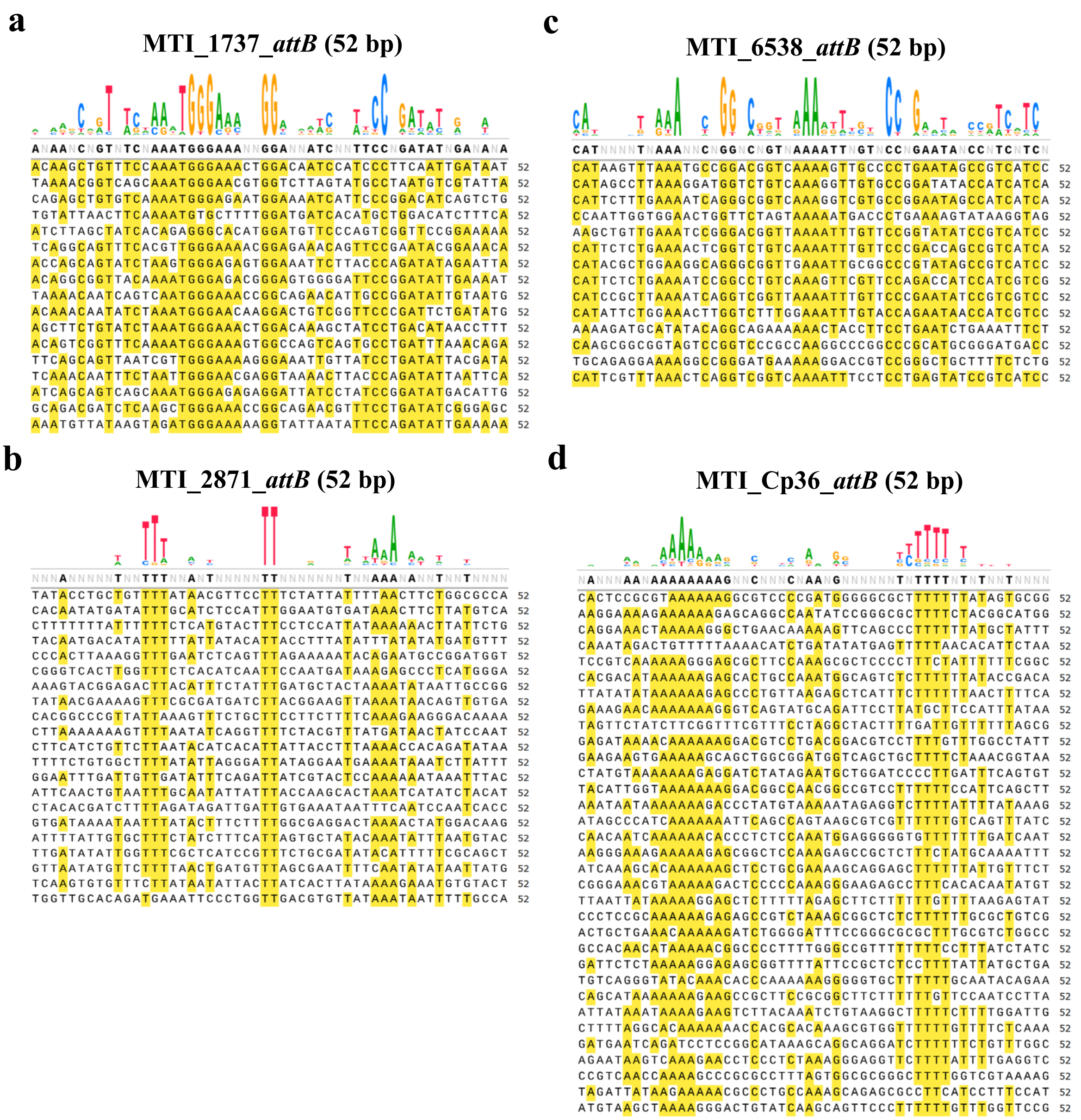

Figure S5. Schematic of an alignment of diverse *attB* sequences that are targeted by four tested MTIs. Each target sequence is aligned with respect to the core dinucleotide (GG, TT or AA). Sequence logo above the alignment indicates conservation across target native or previously identified *attB* sites. The alignment is colored according to the consensus.

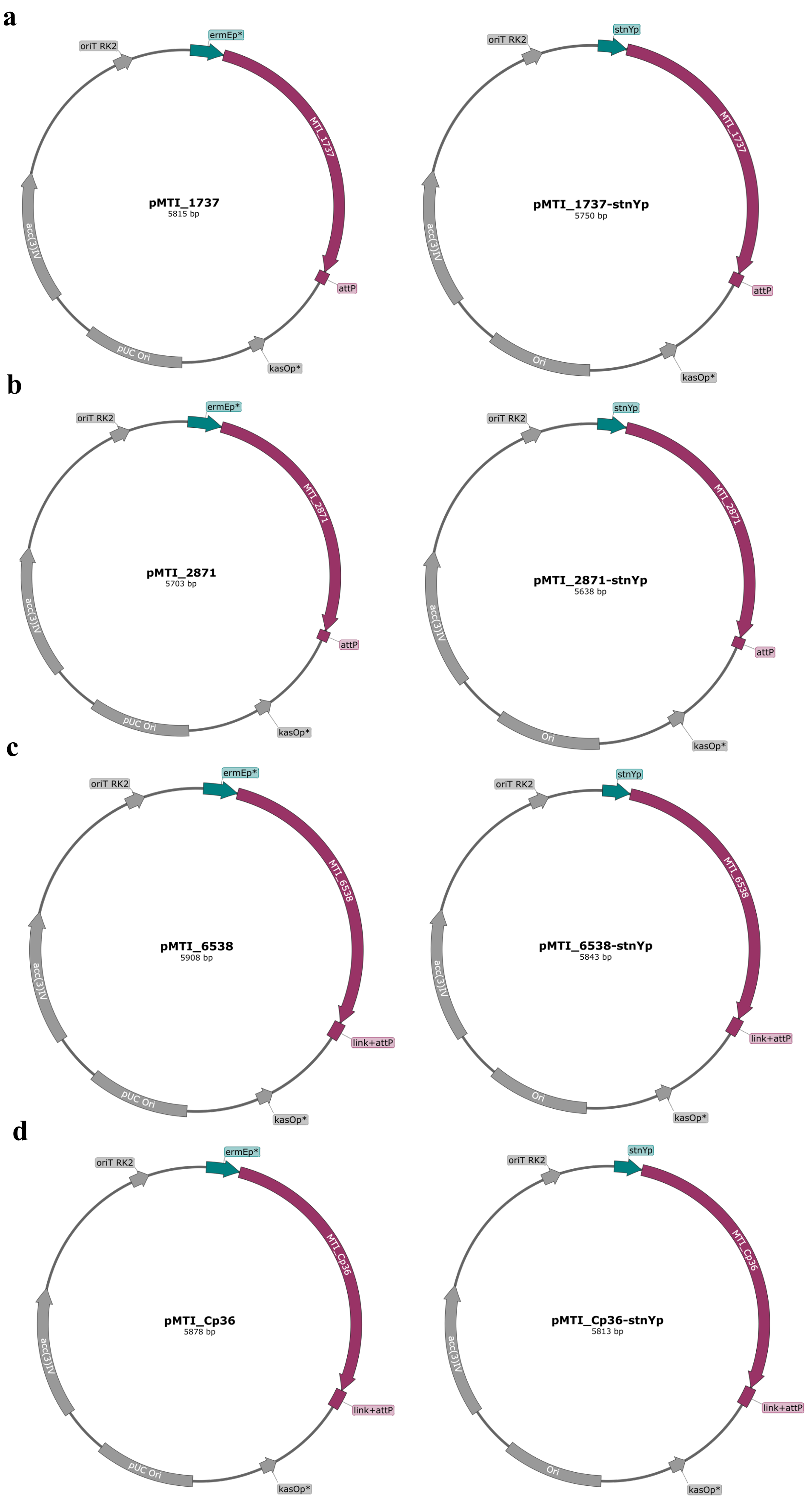

Figure S6. Genetic maps of eight MTI-series plasmids. **a**, Genetic maps of pMTI_1737 and pMTI_1737-*stnYp*; **b**, Genetic maps of pMTI_2871 and pMTI_2871-*stnYp*; **c**, Genetic maps of pMTI_6538 and pMTI_6538-*stnYp*; **d**, Genetic maps of pMTI_Cp36 and pMTI_Cp36-*stnYp*.

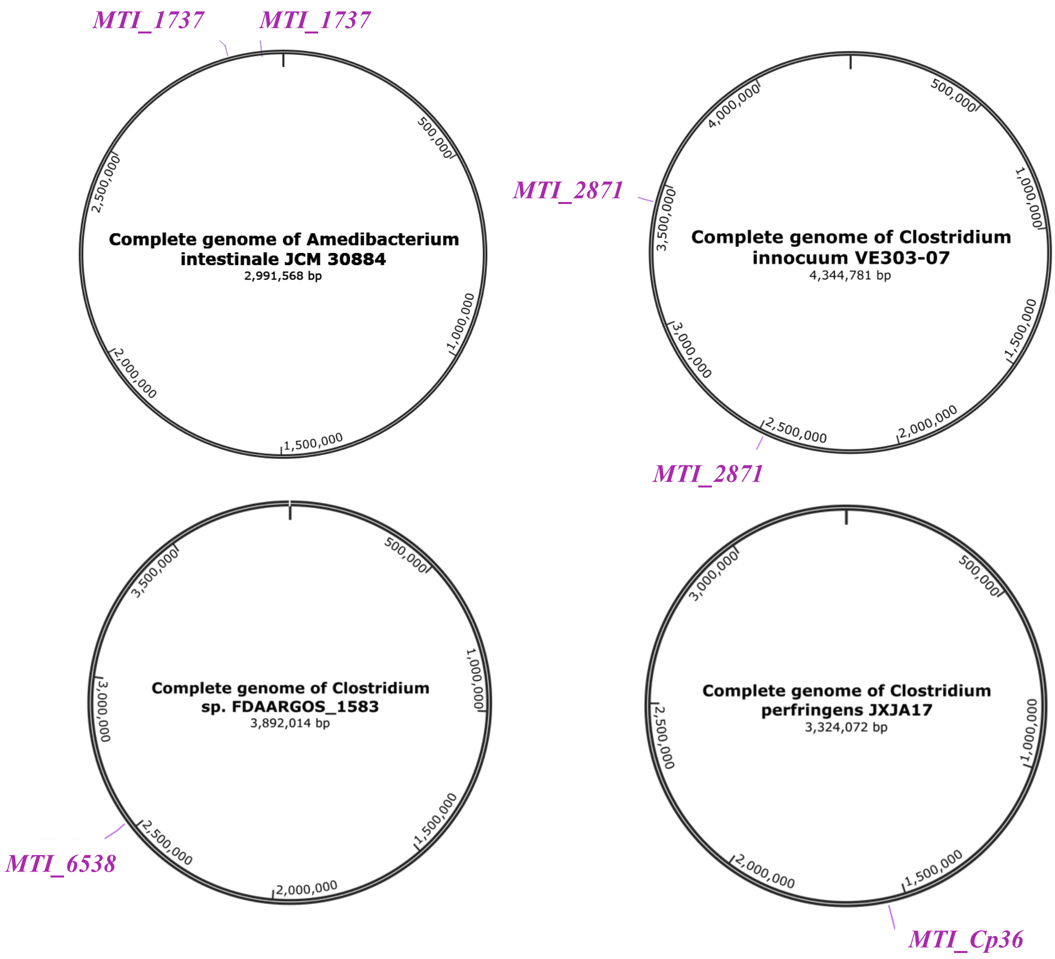

Figure S7. Chromosomal positions for MGE integration mediated by four different MTIs in native hosts

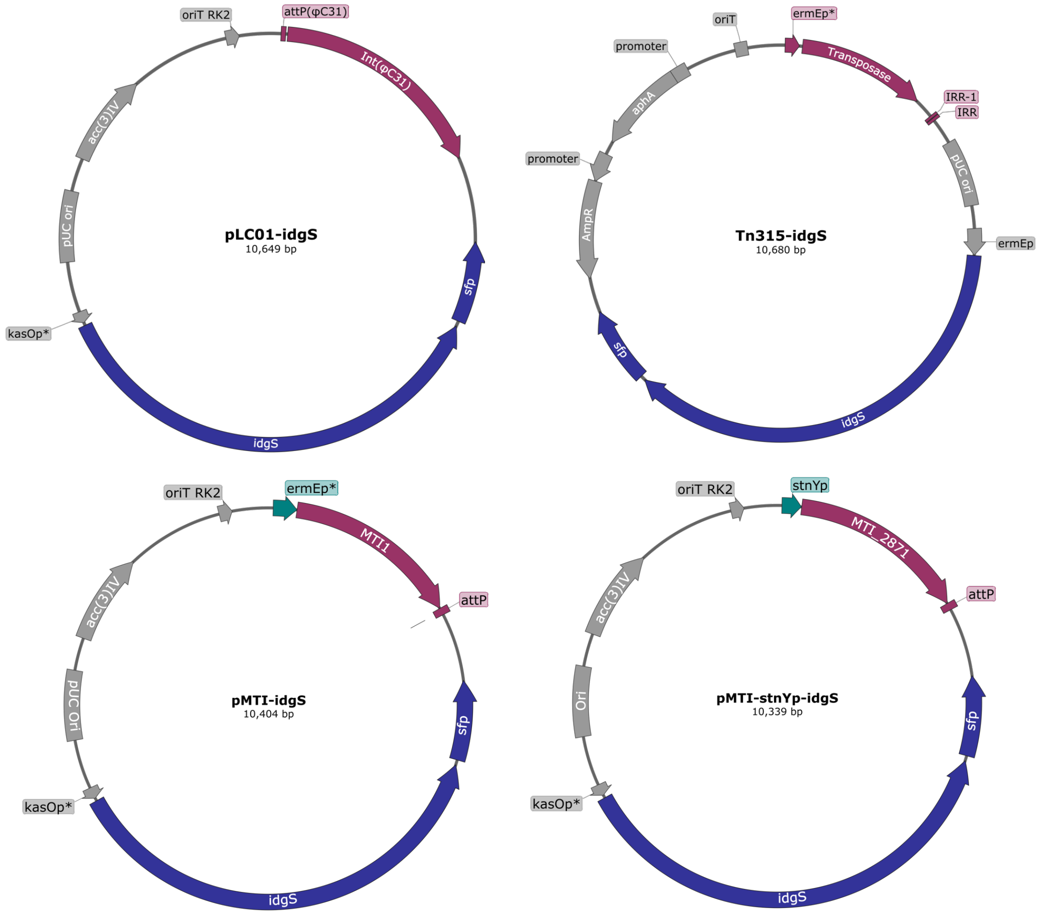

Figure S8: Genetic maps of the *idgS*-*sfp*-expressing plasmids pLC01-*idgS*, Tn315-*idgS*, pMTI-*idgS* and pMTI-*stnYp*-*idgS*

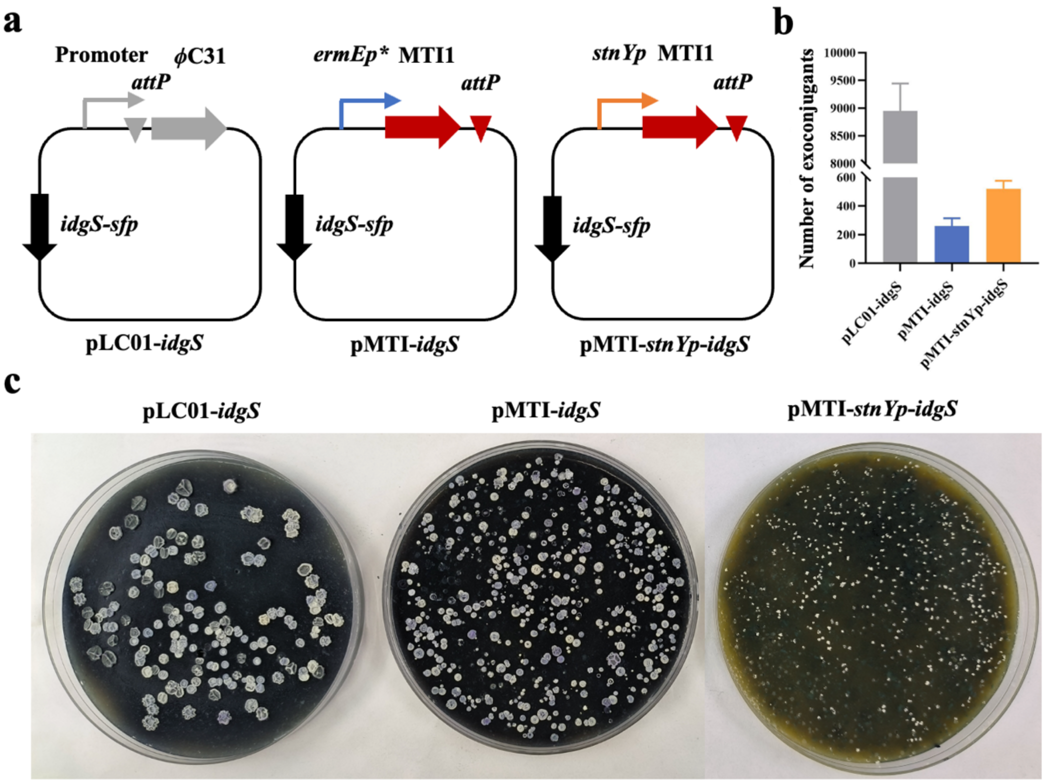

Figure S9. Integration of *idgS-sfp* mediated by MTI1 under the contol of two different promoters in *S. albus*. **a**, Genetic maps of the *idgS-sfp*-expressing plasmids with MTI1 under the control of the two different strong promoters (*ermEp** and *stnYp*). **b**, Integration efficiency of *idgS-sfp* mediated by MTI1 under the control of the two different strong promoters in *S. albus* J1074. **c**, Growth phenotypes of *S. albus* J1074 when integrating *idgS-sfp* mediated by MTI1 under the control of the two different strong promoters. The plasmid pLC01-*idgS* was used as the control.

a

	Day 0	Day 1	Day 2	Day 3	Day 4	Day 5
J1074/pLC01-*idgS*-1	0.0	34.5	78.9	110.3	189.2	167.5
J1074/pLC01-*idgS-2*	0.0	42.7	89.9	122.9	193.1	173.0
J1074/pMTI-*idgS* No. 1-1	0.0	10.1	46.0	67.5	86.6	111.5
J1074/pMTI-*idgS* No. 1-2	0.0	0.0	14.1	33.1	34.0	42.4
J1074/pMTI-*idgS* No. 1-3	0.0	104.2	183.1	258.5	350.4	315.5
J1074/pMTI-*idgS* No. 1-4	0.0	63.3	89.3	132.2	139.9	118.6
J1074/pMTI-*idgS* No. 1-5	0.0	43.1	82.9	108.2	104.8	143.0
J1074/pMTI-*idgS* No. 1-6	0.0	51.8	70.8	109.1	190.1	168.4
J1074/pMTI-*idgS* No. 1-7	0.0	46.9	66.5	98.5	101.1	85.2
J1074/pMTI-*idgS* No. 1-8	0.0	18.7	73.8	73.6	75.7	104.3
J1074/pMTI-*idgS* No. 1-9	0.0	59.0	89.9	139.0	224.7	181.9
J1074/pMTI-*idgS* No. 1-10	0.0	24.4	79.1	95.4	130.5	150.0
J1074/pMTI-*idgS* No. 1-11	0.0	53.0	85.0	121.3	138.9	127.1
J1074/pMTI-*idgS* No. 1-12	0.0	67.4	130.8	145.8	150.1	118.5
J1074/pMTI-*idgS* No. 1-13	0.0	45.1	110.8	145.2	159.8	217.3
J1074/pMTI-*idgS* No. 1-14	0.0	57.2	76.7	82.9	106.7	124.1

b

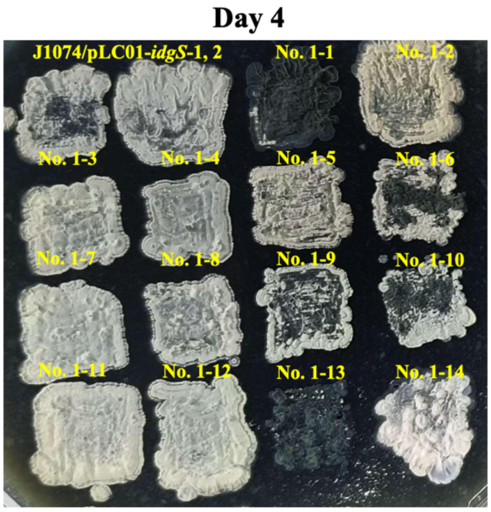

c

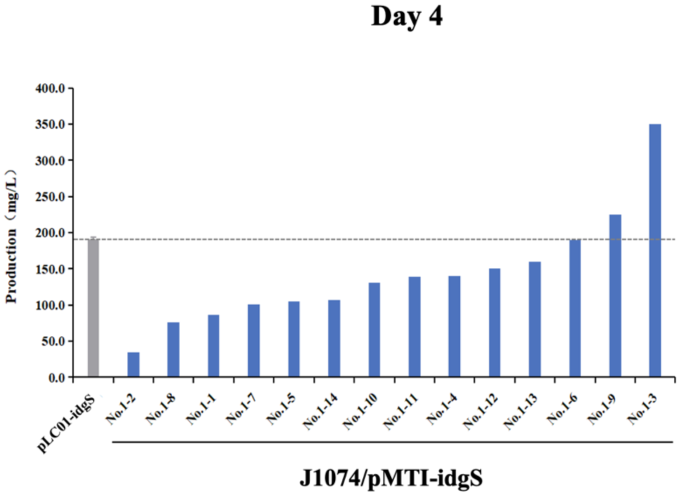

Figure S10. Indigoidine titers and growth phenotypes of 14 MTI1-mediated, *idgS-sfp*-integrated *S. albus* exconjugants. **a**, Time-course production titers of the blue pigment indigoidine in 14 MTI1-mediated, *idgS-sfp*-integrated exconjugants of *S. albus* J1074. **b**, Growth phenotypes of 14 MTI1-mediated, *idgS-sfp*-integrated exconjugants of *S. albus* J1074. **c**, Indigoidine titers of 14 MTI1-mediated, *idgS-sfp*-integrated exconjugants of *S. albus* J1074 on the fourth day. J1074/pLC01-*idgS* was used as the control.

a

attB	Integrated position	Gene function	Strain No.
A	XNR_0005	Dihydrolipoamide S-succinyltransferase	1-5
B	XNR_0236	Heat shock protein	1-14
C	258 bp downstream of XNR_0919	Isocitrate dehydrogenase	1-2 and 1-8
D	XNR_1174	Putrescine transport system permease protein	1-11
E	XNR_1837	Beta-hexosaminidase	1-3
G	XNR_2089	Hypothetical protein	1-7 and 1-12
H	338 bp upstream of XNR_2188	Hypothetical protein	1-9
I	XNR_2396	Threonine synthase	1-13
J	100 bp upstream of XNR_2412	Dimeric protein	1-13
K	XNR_2799	ATP/GTP-binding protein	1-1
L	XNR_3373	AAA ATPase	1-4
M	107 bp downstream of XNR_3571	Membrane protein	1-6
N	82 bp upstream of XNR_4644	Extracellular solute-binding protein	1-10
O	XNR_5563	DNA-binding protein	1-5 and 1-8
P	XNR_5735	Acyl-coenzyme A oxidase 1, peroxisomal	1-8
Q	XNR_5751	Two-component system response regulator	1-14

b

Strain No.	1-1	1-2	1-3	1-4	1-5	1-6	1-7
attB position	K	C	E	L	A/O	M	G
Indigoidine production (mg/L)	86.6	34.0	350.4	139.9	104.8	190.1	101.1

Strain No.	1-8	1-9	1-10	1-11	1-12	1-13	1-14
attB position	C/O/P	H	N	D	G	I/J	B/Q
Indigoidine production (mg/L)	75.7	224.7	130.5	138.9	150.1	159.8	106.7

c

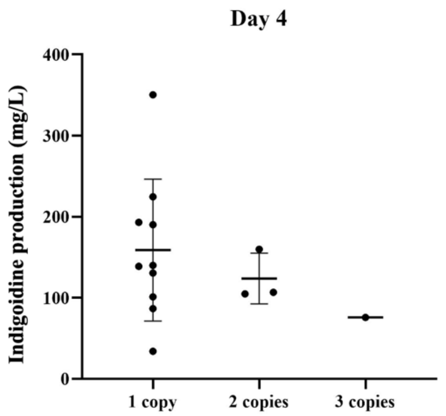

Figure S11. MTI1-mediated integration sites and copy numbers of *idgS-sfp* in 14 independent *S. albus* exconjugants. **a**, MTI1-mediated integration sites of *idgS-sfp* in 14 exconjugants of *S. albus* J1074. **b**, MTI1-integrated copy numbers of *idgS-sfp* in 14 exconjugants of *S. albus* J1074. **c**, Relationship between indigoidine titers and integrated copy numbers of *idgS-sfp* in 14 exconjugants of *S. albus* J1074.

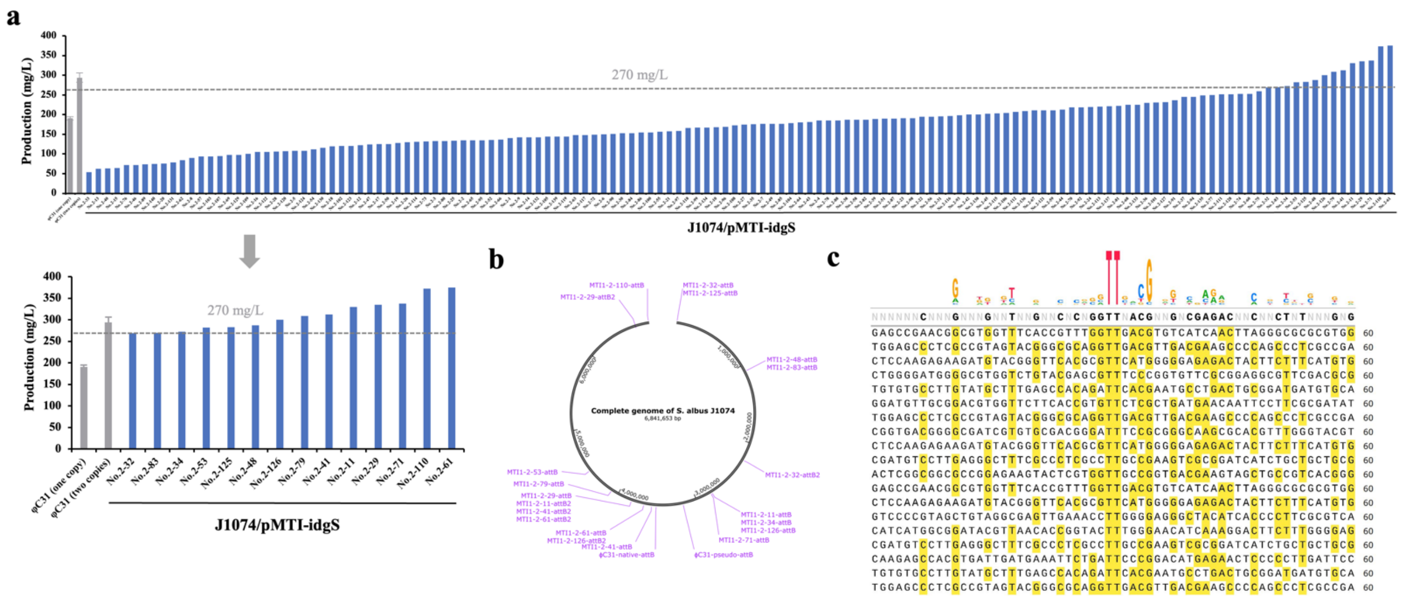

Figure S12. MTI1-mediated integration of *idgS-sfp* in 140 independent *S. albus* exconjugants. a, Indigoidine titers of 14 MTI1-mediated, *idgS-sfp*-integrated exconjugants of *S. albus* J1074 on the fourth day. J1074/pLC01-*idgS*-C1 (one-copy integration of *idgS-sfp*) and J1074/pLC01-*idgS*-C2 (two-copy integration of *idgS-sfp*) were used as the controls. **b**, Genetic map of MTI1-mediated integration of *idgS-sfp* in *S. albus* J1074. A total of 14 independent, *idgS-sfp*-integrated exconjugants that high-efficiently produced indigodine were sequenced. **c**, Schematic of an alignment of diverse *attB* sequences that were targeted by MTI1 for the genomic integration of *idgS-sfp* in *S. albus* J1074.

a

attB site	Integrated position	Gene function	Strain No.
a	XNR_0005	Dihydrolipoamide S-succinyltransferase	2-32 and 2-125
b	258 bp downstream of XNR_0919	Isocitrate dehydrogenase	2-48 and 2-83
c	XNR_1890	LuxR family ranscriptional regulator	2-32
d	XNR_2396	Threonine synthase	2-11, 2-34 and 2-126
e	100 bp upstream of XNR_2412	Dimeric protein	2-71
f	XNR_3133	Peptidase C14 caspase catalytic subunit	2-41
g	15 bp upstream of XNR_3207	Hydrolase	2-61 and 2-126
h	XNR_3373	AAA ATPase	2-11, 2-29, 2-41 and 2-61
i	107 bp downstream of XNR_3571	Membrane protein	2-79
j	XNR_3922	Integral membrane protein	2-53
k	XNR_5858	t1PKS-NRPS gene cluster	2-29
l	XNR_5933	hypothetical protein	2-110

b

Strain No.	2-11	2-29	2-32	2-34	2-41	2-48	2-53
attB position	d/h	h/k	a/c	d	f/h	b	j
Indigoidine production (mg/L)	330.3	335.5	269.9	272.3	312.3	287.7	281.9

Strain No.	2-61	2-71	2-79	2-83	2-110	2-125	2-126
attB position	g/h	e	i	b	l	a	d/g
Indigoidine production (mg/L)	375.5	337.4	309.0	269.7	372.9	283.2	300.0

c

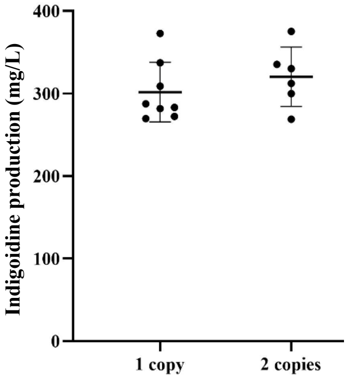

d

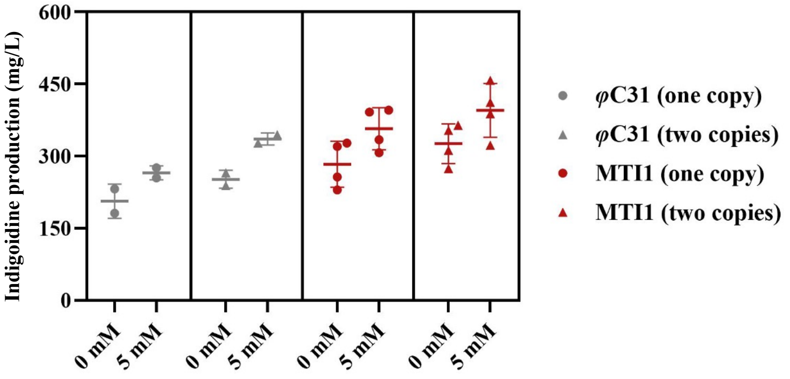

Figure S13. MTI1-mediated integration sites and copy numbers of *idgS-sfp* in 14 independent *S. albus* exconjugants that high-efficiently produced indigodine. a, MTI1-mediated integration sites of *idgS-sfp* in 14 selected exconjugants of *S. albus* J1074. **b**, MTI1-integrated copy numbers of *idgS-sfp* in 14 selected exconjugants of *S. albus* J1074. **c**, Relationship between indigoidine titers and integrated copy numbers of *idgS-sfp* in 14 selected exconjugants of *S. albus* J1074. **d**, Relationship between indigoidine titers and integrated copy numbers of *idgS-sfp* in 14 selected exconjugants of *S. albus* J1074 after feeding 5 mM L-Glutamine. J1074/pLC01-*idgS*-C1 (one-copy integration of *idgS-sfp*) and J1074/pLC01-*idgS*-C2 (two-copy integration of *idgS-sfp*) were used as the controls.

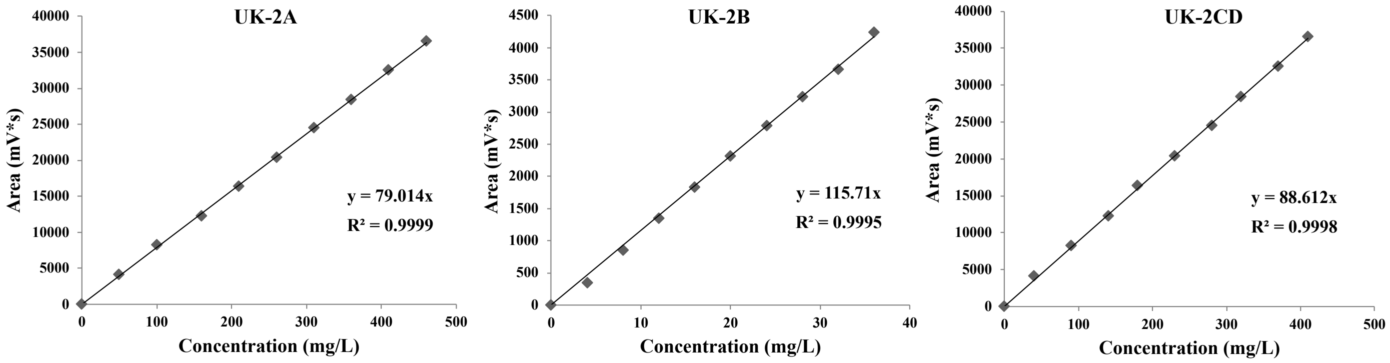

Figure S14. Standard curves of UK-2A, UK-2B and UK-2CD.

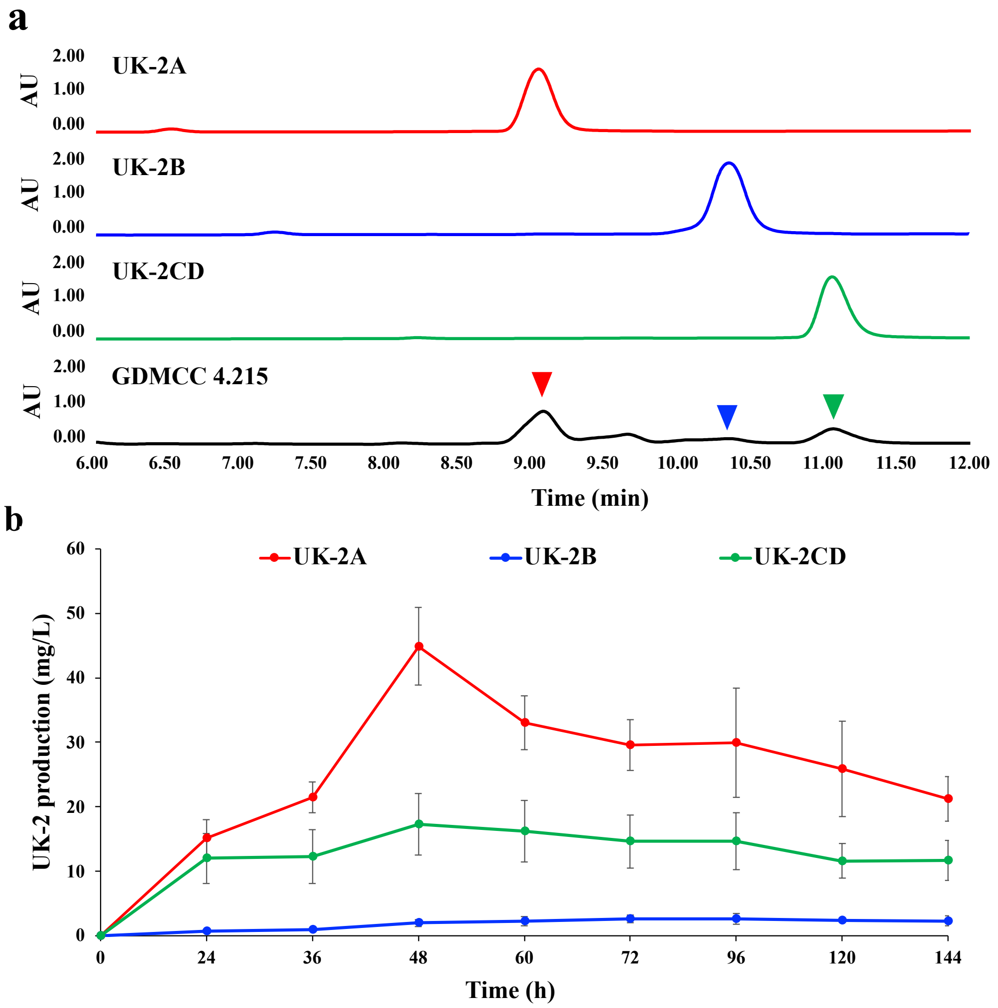

Figure S15. HPLC analysis of UK-2 standards and UK-2 production in *S. huiliensis* GDMCC 4.215. **a**, HPLC analysis of UK-2 standards and UK-2 production of *S. huiliensis* GDMCC 4.215. **b**, Time-course UK-2 production titers of *S. huiliensis* GDMCC 4.215 in the MS medium.

a

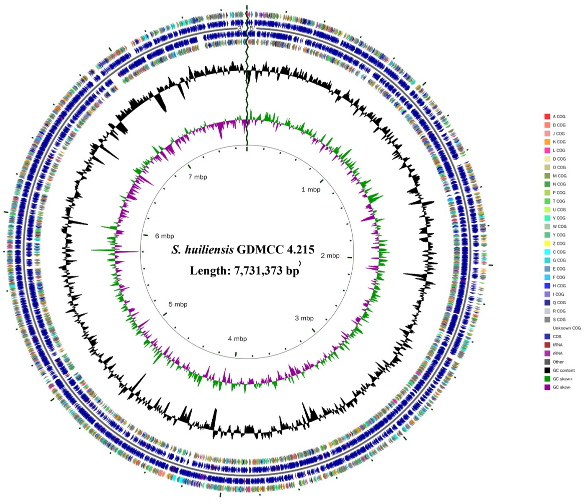

From the inside out of the figure:

The first circle represents the scale;

The second circle represents the GC Skew;

The third circle represents the GC content;

The fourth and seventh circles represent the COG to which each CDS belongs;

The fifth and sixth circles represent the positions of CDS, tRNA and rRNA on the genome.

b

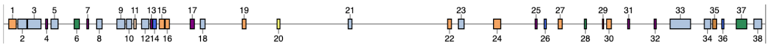

Cluster	Position of cluster (start)	Position of cluster (termination)	Most similar known cluster	Type of gene cluster	Similarity/%
1	55,721	131,912	A54145	NRP	6%
2	147,316	239,326	lagmysin	RIPP	80%
3	246,430	382,634	cyclothiazomycin b1	RIPP:Thiopeptide	100%
4	429,896	449,328	-	-	-
5	483,828	556,018	simocyclinone D8	Saccharide + Polyketide:Modular type I + Polyketide: Type II + Other-Aminocoumarin	18%
6	719,147	772,193	matlystatin A	NRP	55%
7	846,886	865,537	-	-	-
8	946,552	1,001,998	gobichelin A / gobichelin B	NRP	22%
9	1,151,886	1,233,124	napyradiomycin	Terpene	23%
10	1,248,958	1,302,472	hopene	Terpene	76%
11	1,324,156	1,347,845	AT2433 - A1	Alkaloid	14%
12	1,402,122	1,476,332	nataxazole	Polyketide	48%
13	1,492,567	1,512,817	kanamycin	Saccharide	53%
14	1,514,960	1,553,834	moomysin	RIPP	50%
15	1,581,815	1,634,401	paulomycin	Other	11%
16	1,641,629	1,685,002	meilingmycin	Polyketide	3%
17	1,895,521	1,937,929	5-isoprenylindole-3-carboxylate β-D-glycosyl ester	Other	14%
18	1,998,234	2,046,767	kinamycin	Polyketide	20%
19	2,423,373	2,461,749	violapyrone B	Polyketide	28%
20	2,776,849	2,806,406	chejuenolide A/chejuenolide B	Polyketide	7%
21	3,498,290	3,536,163	vazabitide A	NRP	10%
22	4,506,064	4,545,541	merochlorins	-	4%
23	4,615,137	4,673,457	5-isoprenylindole-3-carboxylate β-D-glycosyl ester	Other	19%
24	4,972,229	5,044,777	aranciamycin	Polyketide: Type II + Saccharide Hybrid tailoring	78%
25	5,393,579	5,414,184	lomofungin	Other	13%
26	5,485,223	5,507,825	anantin C	RIPP	75%
27	5,627,501	5,668,625	-	-	-
28	5,893,253	5,913,999	albonoursin	Other	50%
29	6,076,659	6,088,320	-	-	-
30	6,118,697	6,166,898	abyssomicin C/atrop-abyssomicin C	Polyketide: Modular type I	10%
31	6,331,655	6,352,150	-	-	-
32	6,601,045	6,622,283	-	-	-
33	6,762,325	6,970,119	piericidin A1	Polyketide	100%
34	7,109,339	7,174,315	A-503083 A/A-503083 B/A-503083 E/A - 503083 F	NRP	7%
35	7,193,110	7,234,267	hygromycin A	Saccharide	75%
36	7,286,153	7,308,650	ketomemicin B3/ketomemicin B4	Other	100%
37	7,432,975	7,540,529	qinichelins	-	83%
38	7,611,800	7,691,630	pyridomycin	NRP + Polyketide	19%
37	7,432,975	7,540,529	qinichelins	-	83%

Figure S16. Genetic map and antiSMASH analysis of the complete genome of *S. huiliensis* GDMCC 4.215. a, Genetic map of the complete genome of *S. huiliensis* GDMCC 4.215. **b**, antiSMASH analysis for BGC annotation of the complete genome of *S. huiliensis* GDMCC 4.215.

a

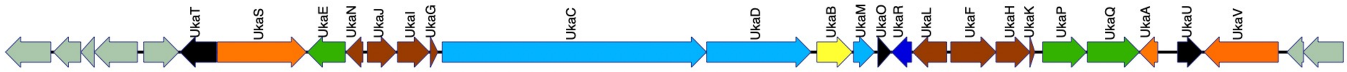

b

ORF	Proposed function	ORF	Proposed function
A	LuxR-family regulator	L	P450 hydroxylase
B	Acyltransferase	M	Ketoreductase
C	NRPS: C-C*-A-T-C-A-KR-T	N	Methyltransferase
D	PKS: KS-AT-T-TE	O	N-acetyltransferase
E	Crotonyl-CoA reductase	P	Ammonia lyase
F	AMP-binding protein	Q	CoA ligase like protein
G	Acyl carrier protein (T)	R	Thioesterase
H	Lysine 2-aminotransferase	S	SARP-family regulator
I	Sarcosine oxidase	T	Alkene reductase
J	Oxidoreductase	U	NAD(P)H-binding protein
K	Tautomerase	V	LuxR family regulator

c

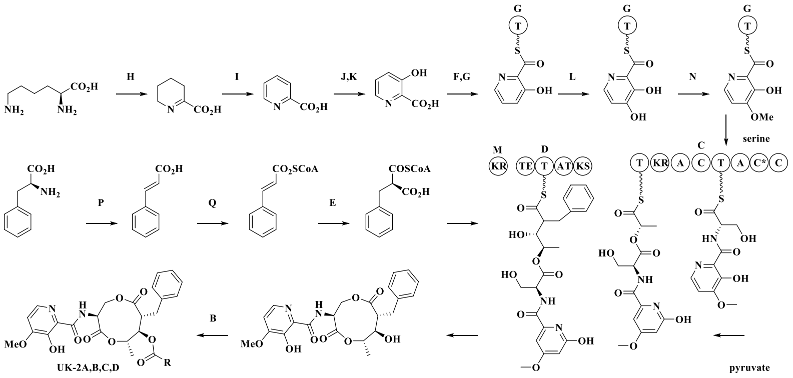

Figure S17. Biosynthetic gene cluster and proposed pathway of UK-2. a, Gene organization of the UK-2 BGC. **b**, Proposed functions of the *uka* genes. **c**, Predicted biosynthetic pathway of UK-2. A, adenylation; C, condensation; C*, inactive condensation; T, thiolation; TE, thioesterase; AT, acyltransferase; KR, ketoreductase; KS, ketosynthase.

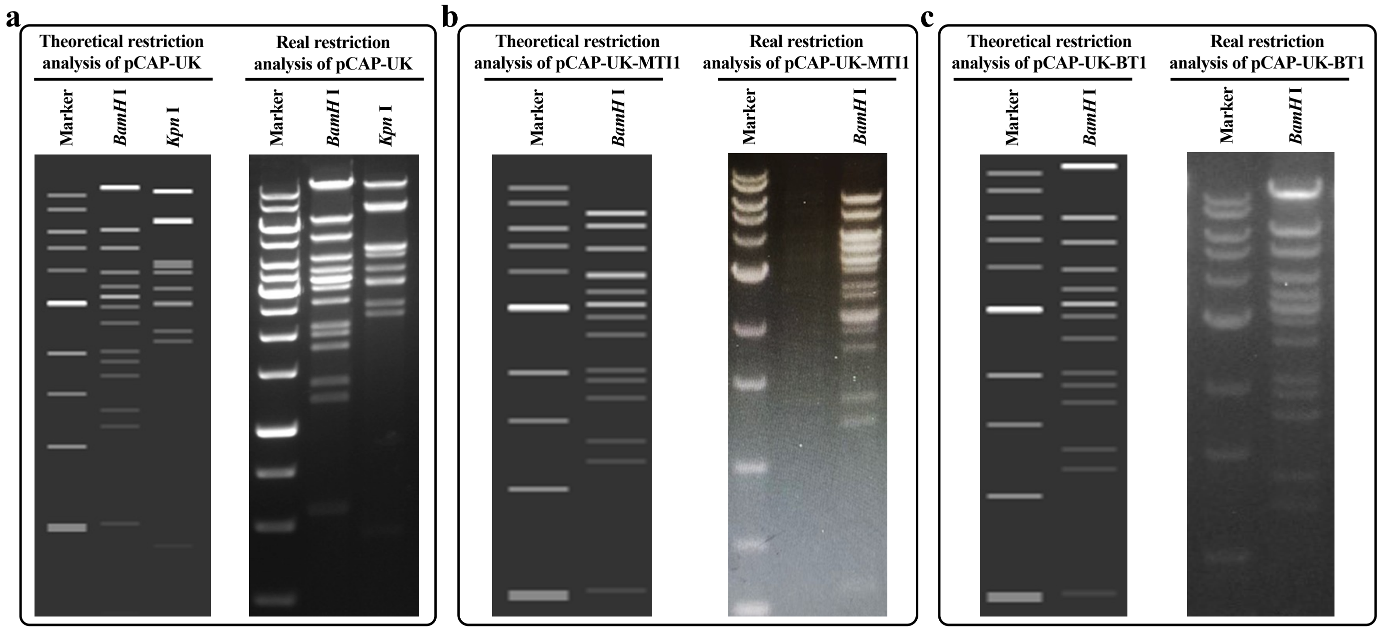

Figure S18. Restriction analysis of the plasmids pCAP-UK (a), pCAP-UK-MTI1(b) and pCAP-UK-BT1 (c).

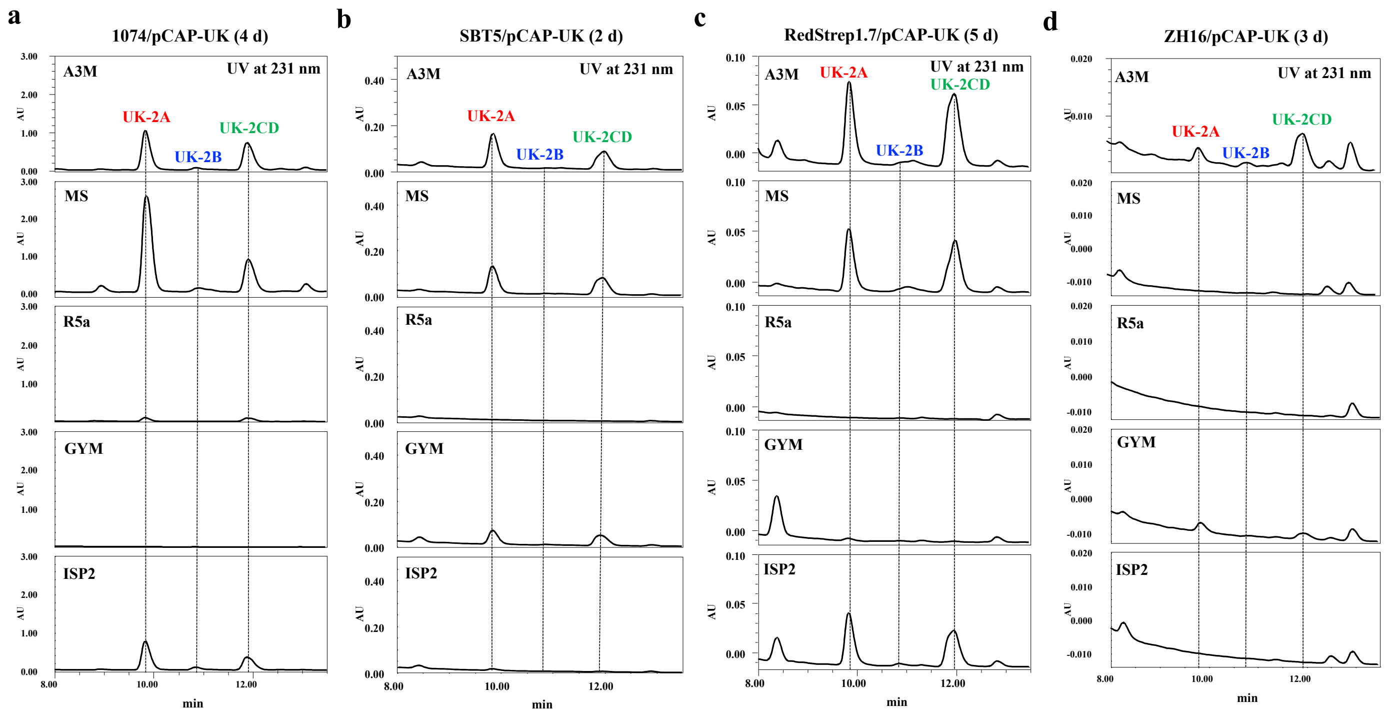

Figure S19. HPLC analysis of UK-2 production in four heterologous *Streptomyces* hosts. a, HPLC analysis of UK-2 production when the UK BGC was heterologously expressed in *S. albus* J1074 in the five media (A3M, MS, R5a, GYM and ISP2). **b**, HPLC analysis of UK-2 production when the UK BGC was heterologously expressed in *S. lidivans* SBT5 in the five media (A3M, MS, R5a, GYM and ISP2). **c**, HPLC analysis of UK-2 production when the UK BGC was heterologously expressed in *S. lividans* RedStrep1.7 in the five media (A3M, MS, R5a, GYM and ISP2). **d**, HPLC analysis of UK-2 production when the UK BGC was heterologously expressed in *S. atratus* ZH16 in the five media (A3M, MS, R5a, GYM and ISP2).

a

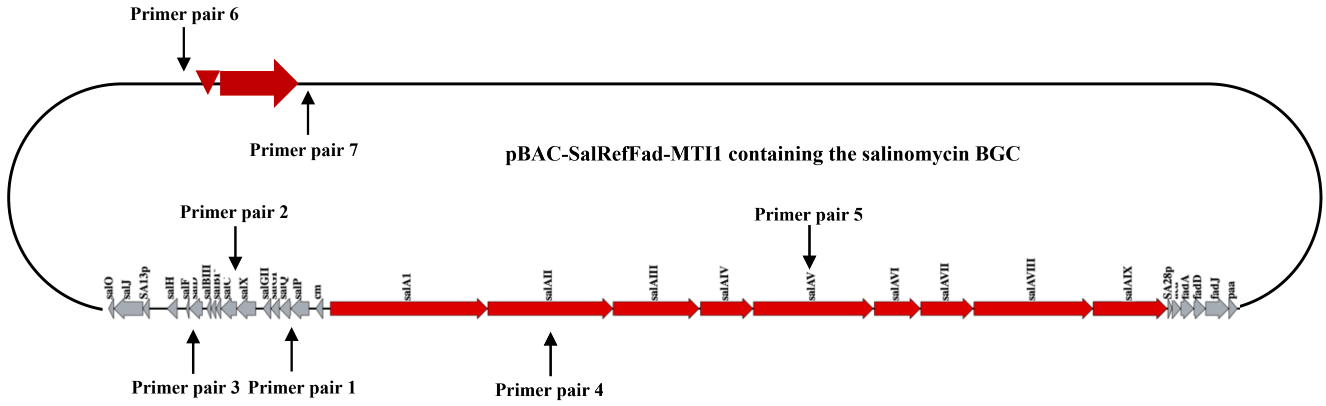

b

Primer pair	Primer name
Primer pair 1	ID-salQP-fw
	ID-salQP-rev
Primer pair 2	ID-salCX-fw
	ID-salCX-rev
Primer pair 3	ID-salFD-fw
	ID-salFD-rev
Primer pair 4	ID-salAII-fw
	ID-salAII-rev
Primer pair 5	ID-salAV-fw
	ID-salAV-rev
Primer pair 6	ID-MTI1-up-fw
	ID-MTI1-up-rev
Primer pair 7	ID-MTI1-down-fw
	ID-MTI1-down-rev

c

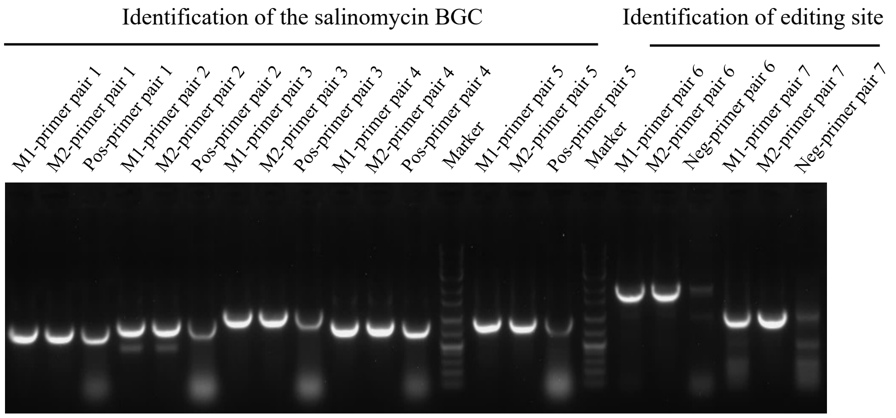

Pos: pBAC-SalRefFad, positive control; Neg: pHZ, negative control; M1/M2: pBAC-SalRefFad-MTI1

Figure S20. Identification of the edited plasmid pBAC-SalRefFad-MTI1. **a**, Map of the edited plasmid pBAC-SalRefFad-MTI1. **b**, Primer paris for confirming the correction of the edited plasmid pBAC-SalRefFad-MTI1. **c**, PCR identification for the correction of the edited plasmid pBAC-SalRefFad-MTI1.

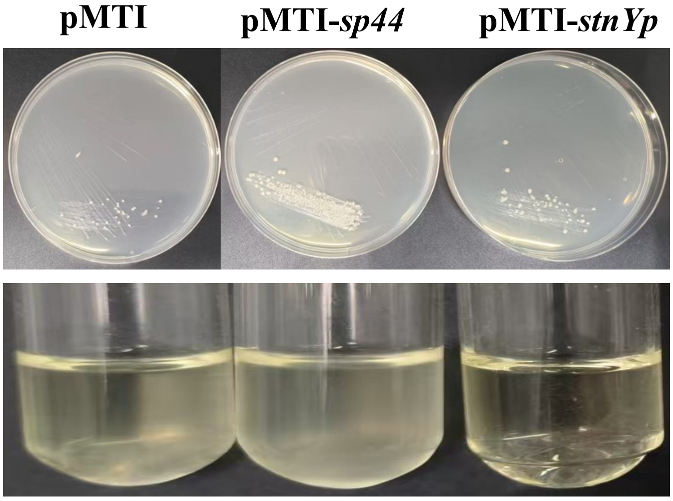

Figure S21. Growth phenotypes of *B. gladioli* ATCC 10248 when introducing three strong promoters controlled MTI1-series plasmids. The expression of MTI1 in the plasmids pMTI, pMTI-*sp44* and pMTI-*stnYp* was under the control of three strong promoters *ermEp**, *sp44* and *stnYp*, respectively. The solid (up) and liquid (down) CYMG media were used for observing growth phenotypes.

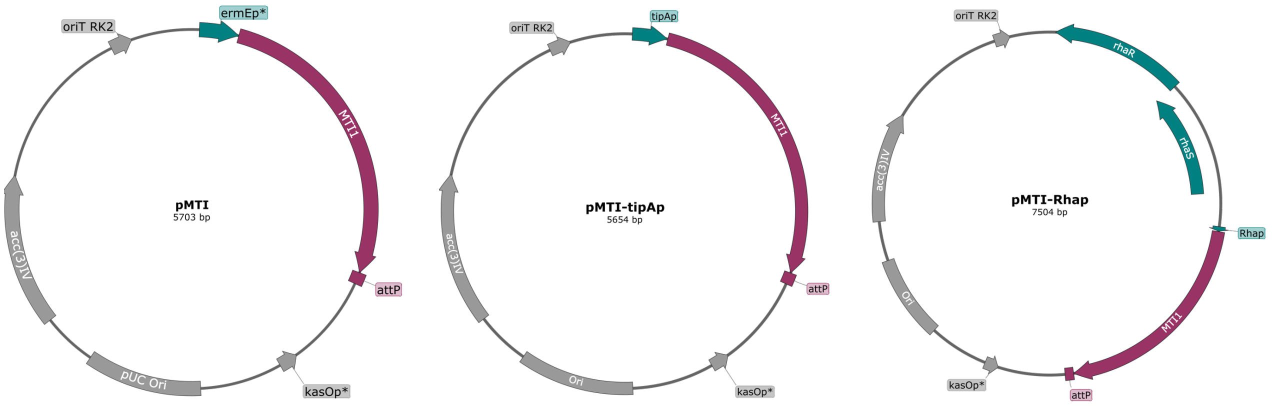

Figure S22. Genetic maps of the plasmids pMTI, pMTI-*tipAp* and pMTI-*Rhap*.

a

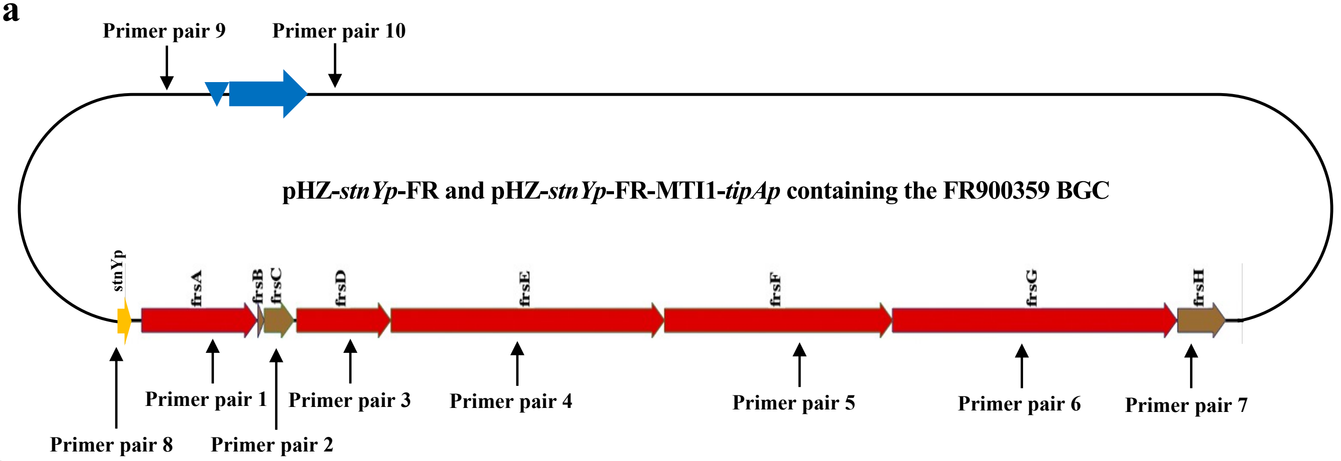

b

Primer pair	Name
Primer pair 1	ID-frsA-fw
	ID-frsA-rev
Primer pair 2	ID-frsBC-fw
	ID-frsBC-rev
Primer pair 3	ID-frsD-fw
	ID-frsD-rev
Primer pair 4	ID-frsE-fw
	ID-frsE-rev
Primer pair 5	ID-frsF-fw
	ID-frsF-rev
Primer pair 6	ID-frsG-fw
	ID-frsG-rev
Primer pair 7	ID-frsH-fw
	ID-frsH-rev
Primer pair 8	ID-*stnYp*-fw
	ID-*stnYp*-rev
Primer pair 9	ID-*tipAp*-fw
	ID-*tipAp*-rev
Primer pair 10	ID-MTI1-fw
	ID-MTI1-rev

c

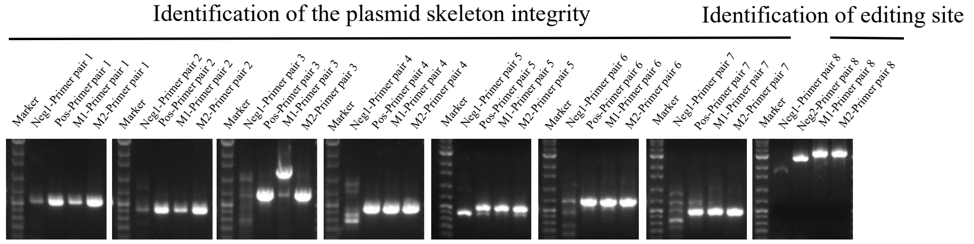

Pos: pHZ-FR900359-3C6, positive control; Neg: pHZ, negative control; M: pHZ-*stnYp*-FR

d

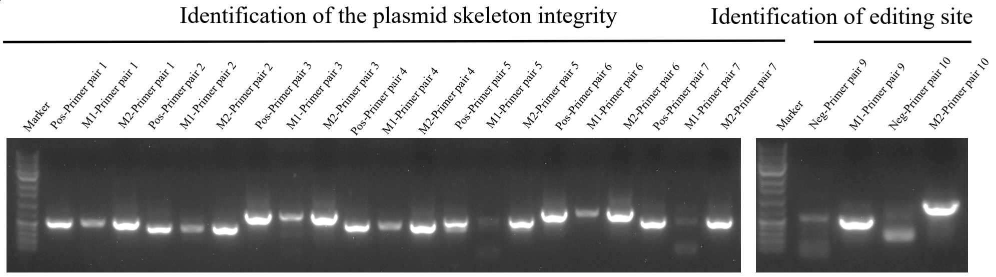

Pos: pHZ-*stnYp*-FR, positive control; Neg: pHZ, negative control; M: pHZ-*stnYp*-FR-*stnYp*-MTI1-*tipAp*

Figure S23. Identification of the plasmids pHZ-*stnYp*-FR and pHZ-*stnYp*-FR-MTI1-*tipAp*. **a**, Maps of the plasmids pHZ-*stnYp*-FR or pHZ-*stnYp*-FR-MTI1-*tipAp*. **b**, Primer paris for confirming the correction of the edited plasmids pHZ-*stnYp*-FR or pHZ-*stnYp*-FR-MTI1-*tipAp*. **c**, PCR identification for the correction of the plasmid pHZ-*stnYp*-FR. **d**, PCR identification for the correction of the plasmid pHZ-*stnYp*-FR-MTI1-*tipAp*.

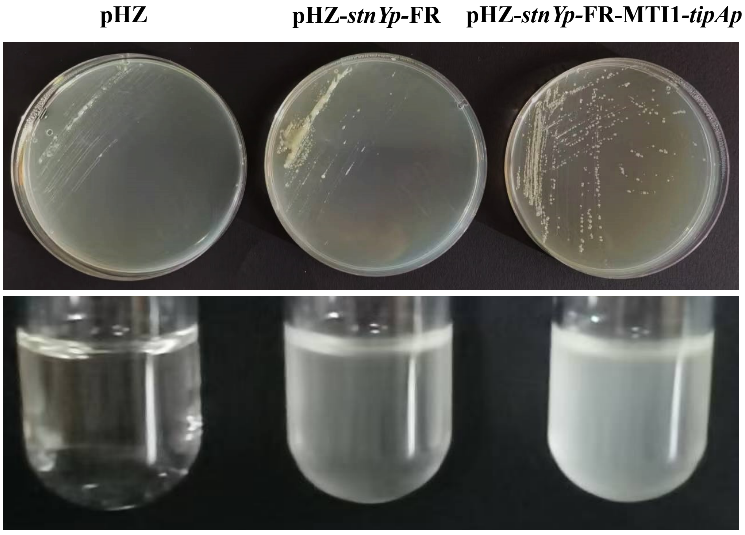

Figure S24. Growth phenotypes of *B. gladioli* ATCC 10248 when introducing pHZ, pHZ-*stnYp*-FR or pHZ-*stnYp*-FR-MTI1-*tipAp*. The solid (up) and liquid (down) CYMG media were used for observing growth phenotypes.

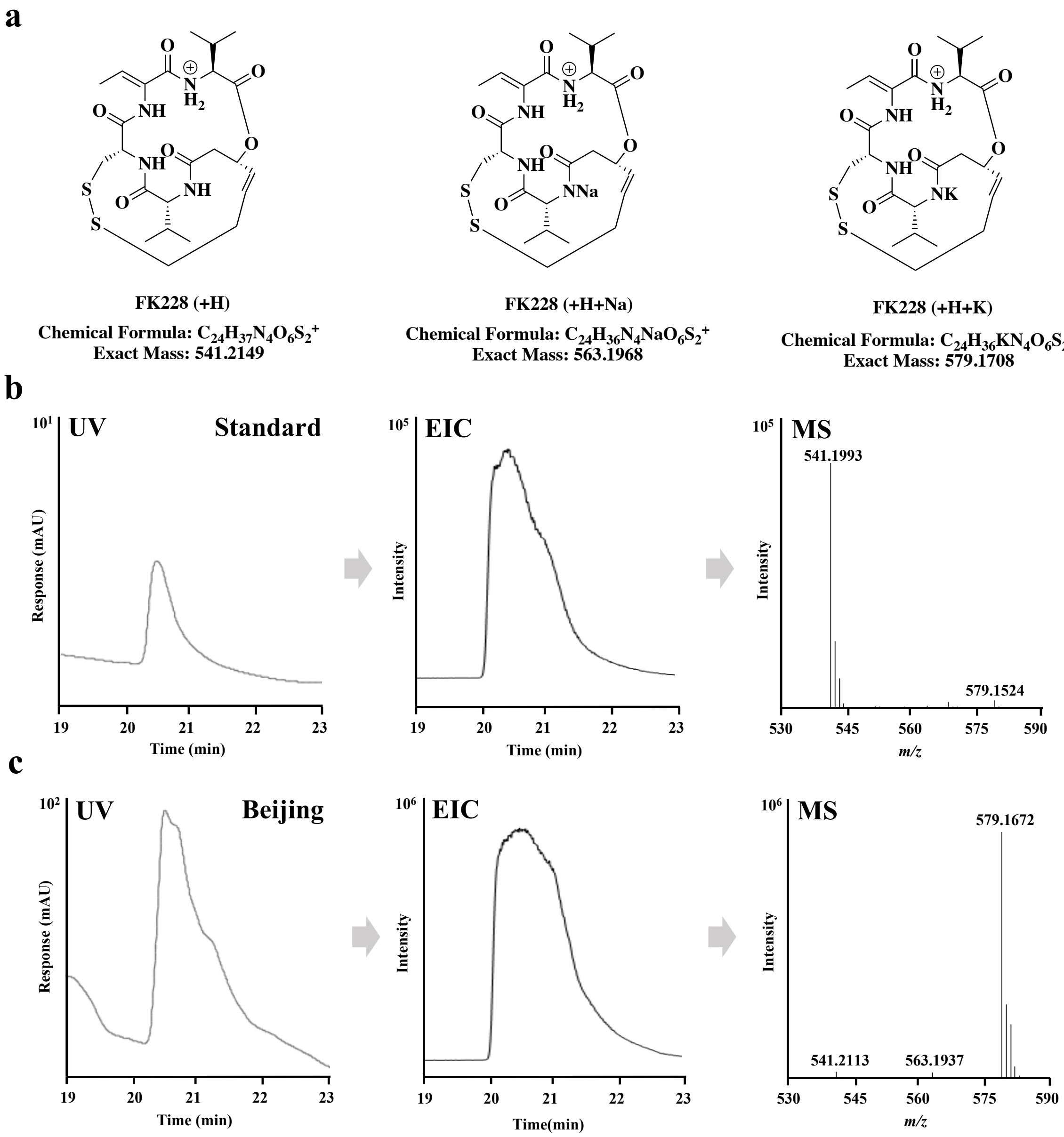

Figure S25. LC-MS analysis of FK228 standard and FK228 production in *Chromobacterium* sp. Beijing. a, The chemical structures of FK228 (+H), FK228 (+Na) and FK228 (+K); **b**, LC-MS analysis of FK228 standard; **c**, LC-MS analysis FK228 production in *Chromobacterium sp.* Beijing.

a **pMTI** **pMTI-*sp44*** **pMTI-*stnYp*** **b** **pMTI** **pMTI-*tipAp*** **pMTI-*Rhap***

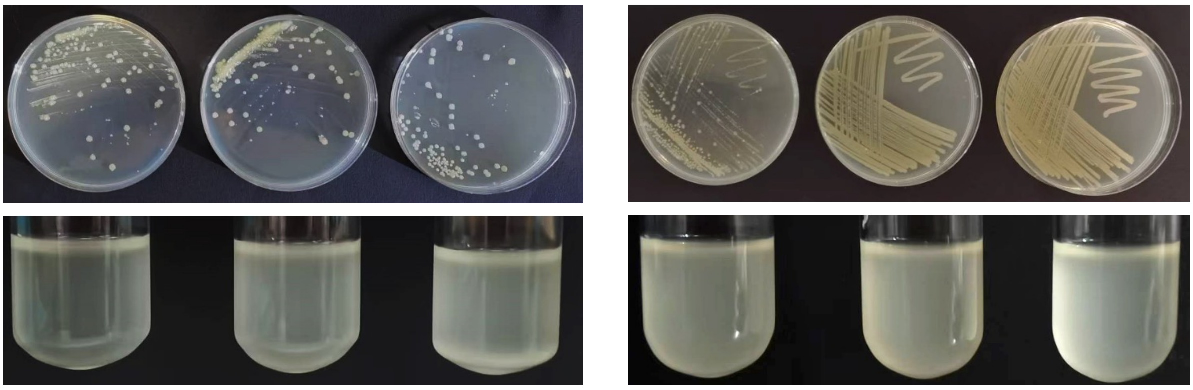

Figure S26. Growth phenotypes of *Chromobacterium* sp. Beijing when introducing strong promoters (a) or weak promoters (b) controlled MTI1-series plasmids. The expression of MTI1 in the plasmids pMTI-*sp44* and pMTI-*stnYp* was under the control of the strong promoters *sp44* and *stnYp*, respectively. The expression of MTI1 in the plasmids pMTI-*tipAp* and pMTI-*Rhap* was under the control of the two weak, inducible promoters *tipAp* and *Rhap*, respectively. The plasmid pMTI with the expression of MTI1 under the control of the strong promoter *ermEp** was used as the control. The solid (up) and liquid (down) CYMG media were used for observing growth phenotypes.

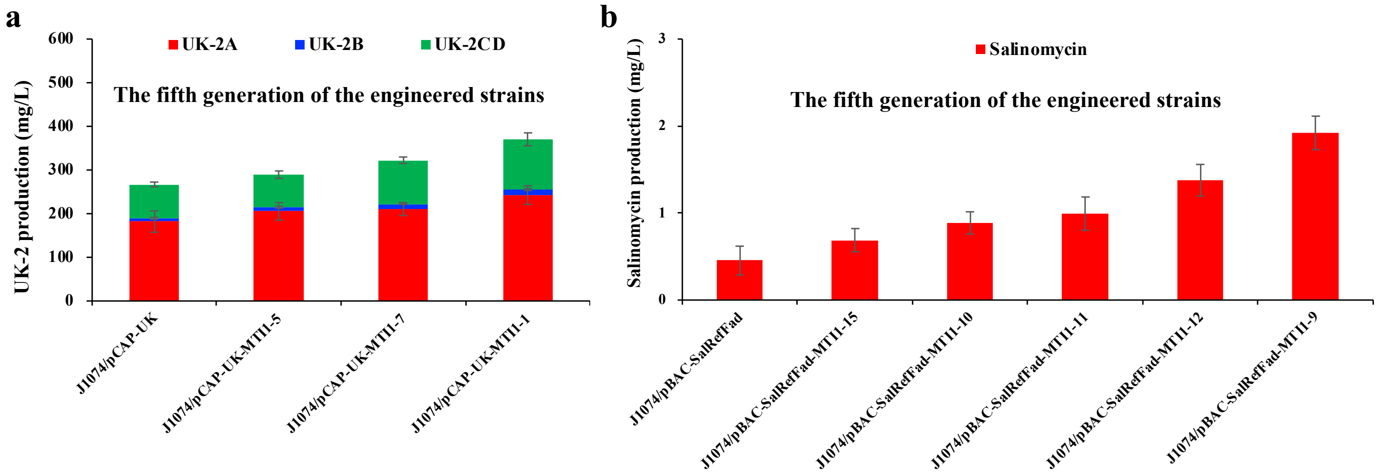

Figure S27. Confirmation of MTI1-mediated exconjugants that high-efficiently produced UK-2 (a) and salinomycin (b) after five-round passages. Fermentation samples of UK-2 and salinomycin for HPLC analysis were collected on the fifth and fourth days, respectively.

Extended methods

Cloning the UK-2 BGC by CRISPR-assisted yeast transformation-associated recombination (TAR)

The UK-2 BGC was cloned from the genome of *S. huiliensis* GDMCC 4.215 using the CRISPR/Cas9-assisted TAR cloning approach in yeast as described previously and pCAP01 was used as the cloning vector (Lee et al., 2015). Using the genome of *S. huiliensis* GDMCC 4.215 as DNA template, the upstream and downstream homologous arms of the UK-2 BGC were amplified using the primer pairs pCAP-UK-up-fw/rev and pCAP-UK-down-fw/rev, respectively. Then, the two PCR products were assembled into the *Kpn*I/*Spe*I-linearized pCAP01 through *in vitro* recombination (2×Ezmax® Ultra Universal CloneMix, Tolo Biotech., China), thus generating the cloning plasmid pCAP-UK-HA. Second, using the plasmid pCB003 as the template, two sgRNA transcription cassettes with the T7 promoter, including sgRNA-UK-DNA-up and sgRNA-UK-DNA-down, were obtained by PCR using the primer pairs UK-up-sRNA-fw/UK-sgRNA-rev and UK-down-sgRNA-fw/UK-sgRNA-rev, respectively. Then, the *in vitro* transcription of the two purified PCR products was conducted using MEGAScriptTM T7 Kit (Thermo Fisher Scientific, USA), followed by purification using MEGAClearTM Kit (Thermo Fisher Scientific, USA), thus generating the two sgRNAs (sgRNA-UK-up and sgRNA-UK-down) targeting the upstream or downstream regions of the UK-2 BGC. Finally, the genomic DNA of *S. huiliensis* GDMCC 4.215 was digested with the sgRNA/Cas9 (TOLO Biotechnology, China) complex for 12 h at 37°C using the cleavage reaction mix (3 μg of GDMCC 4.215 genomic DNA, 6 μg of sgRNA-UK-up, 6 μg of sgRNA-UK-down, 5 μL of 10×Cas9 reaction buffer, 3 μL of Cas9 and DEPC H_2O up to 50 μL). The digested genomic DNA was purified by ethanol precipitation and dissolved in 50 μL of ddH_2O. Meanwhile, the cloning plasmid pCAP-UK-HA was linearized by *Pme* I. Both the purified Cas9/sgRNA-digested GDMCC 4.215 genomic DNA (2 μg) and the linearized pCAP-UK-HA fragment (1 μg) were transformed into the spheroplasts of *S. cerevisiae* VL6-48. The transformation product was mixed with SD-Trp agar (synthetic tryptophan dropout agar) and then overlaid on SD-Trp agar plate, followed by incubation at 30°C for three days. The transformants were verified by PCR using the three primer pairs, including ID-pCAP-UK-1-fw/rev, ID-pCAP-UK-2-fw/rev and ID-pCAP-UK-3-fw/rev. The plasmids from the PCR-positive transformants were extracted using the E.Z.N.A.BAC/PAC DNA Kit (Omega Bio-Tek, USA) and then electroporated into *E. coli* EPI300. Finally, the plasmid pCAP-UK was extracted from *E. coli* EPI300 and confirmed by restriction analysis (Fig. S18a).

Editing of the plasmid pBAC-SalRefFad containing the salinomycin BGC

The iCASRED approach that we developed previously was used for the editing of the plasmid pBAC-SalRefFad containing the salinomycin BGC to generate the plasmid pBAC-SalRefFad-MTI1 (Zheng et al., 2025). The cassette *ermEp**-MTI1-*attP*, the upstream homology arm and the downstream homology arm, were obtained by PCR using the primer pairs Sal-MTI1-fw/rev, Sal-MTI1-up-fw/rev and Sal-MTI1-down-fw/rev, respectively. Then, the three PCR

products were assembled through *in vitro* recombination, thus generating the editing fragment Up-MTI1-Down. Second, the plasmid pCB006 was electroporated into *E. coli* DH10B/pBAC-SalRefFad and the single colony of *E. coli* DH10B/pBAC-SalRefFad/pCB006 was inoculated into 4 mL LB liquid medium. After growth overnight at 37 °C, 100 μL cultures were transferred to 50 mL LB liquid medium in 250 mL flasks. When OD_{600} reached ~0.3, 10 mM arabinose was added to induce the expression of λRed system for 0.5 h. The cultures were collected and washed twice with 10% glycerol. The editing plasmid pCB003-C31 (100-200 ng) and the edited fragment Up-MTI1-Down were electroporated into the competent cells, followed by growth overnight at 30 °C on LB agar plates with 50 μg/mL apramycin and 200 μg/mL spectinomycin. After each transformation experiment, 15 colonies were randomly selected by PCR using the primer pairs (i.e., ID-MTI1-up-fw/rev and ID-MTI1-down-fw/rev) followed by sanger sequencing, thus generating the plasmid pBAC-SalRefFad-MTI1.

Cloning of the FR900359 BGC by constructing BAC library

The FR900359 BGC was cloned from the genome of *Chromobacterium vaccinii* DSM 25150 using the approach for the construction of BAC library as described previously and pHZ was used the cloning vector (Luo et al., 2003 and Xu et al., 2016). Briefly, the genomic DNA of *C. vaccinii* DSM 25150 was prepared in agarose plugs, partially digested with *BamH* I and then separated by pulsed-field gel electrophoresis (PFGE). Then, high-molecular-weight DNA fragments were recovered from the agarose gel by electroelution using a Bio-Rad model 422 Electro-Eluter at 10 mA/tube for 2 h at 4°C in 1×TAE buffer. The plasmid pHZ was also linearized with *BamH* I, followed by gel purification, dephosphorylation and ligation with the partially digested genomic DNA. The ligation mixture was desalted and concentrated on a Millipore 0.025-μm-pore filter with 10% PEG8000 for 2 h at 4°C and electroporated into *E. coli* DH10B, followed by incubation for 12 h at 37°C in LB agar plates with 50 μg/mL apramycin. Then, 384 single BAC colonies were selected for constructing the BAC library. The primer pairs, including ID-FR-3C6-up-fw/rev and ID-FR-3C6-down-fw/rev, were used for selecting the correct BAC constructs which contain the complete FR900359 BGC, thus generating the plasmid pHZ-FR-3C6 (Table S3).

Editing of the plasmid pHZ-FR-3C6 containing the FR900359 BGC

The iCASRED approach that we developed previously was used for the editing of the plasmid pHZ-FR-3C6 to generate the plasmids pHZ-*stnYp*-FR and pHZ-*stnYp*-FR-MTI1-*tipAp* (Zheng et al., 2025). Briefly, the plasmid pCB003 skeleton, the transcription cassette of sgRNA targeting the upstream region of the FR900359 BGC, the promoter *stnYp*, the upstream homology arm and the downstream homology arm were obtained by PCR using the primer pairs pCB003-skeleton-fw/rev, *stnYp*-fw/rev, FR-upstream-gRNA-fw/rev, FR-Rep-up-fw/rev and FR-Rep-down-fw/rev, respectively (Table S3). Then, the five PCR products were assembled through *in vitro*

recombination, thus generating the plasmid pCB003-FR-*stnYp*. Second, the plasmid pHZ-FR-3C6 was electroporated into *E. coli* BL23 and the resulting strain BL23/pHZ-FR-3C6 was inoculated into LB medium. After growth overnight at 37 °C, 100 μL cultures were transferred to 50 mL LB liquid medium. When OD_{600} reached 0.3-0.4, arabinose (10 mM) was added to induce the expression of the λRed system for 30 min. The cultures were then collected and washed twice with 10% glycerol. The editing plasmid pCB003-FR-*stnYp* (~100 ng) was electroporated into the competent cells, followed by growth overnight on LB agar plate with both apramycin (50 μg/mL) and spectinomycin (100 μg/mL). After each transformation experiment, 20 colonies were identified by PCR using the primer pairs (i.e., ID-FR-del-fw/rev and ID-FR-promoter-fw/rev), thus generating the edited plasmid pHZ-*stnYp*-FR. Similarly, the iCASRED approach was also used for constructing the plasmid pHZ-*stnYp*-FR-MTI1-*tipAp*.

References

1. Bai, T. L., Yu, Y. F., Xu, Z. & Tao M. F. Construction of *Streptomyces lividans* SBT5 as an efficient heterologous expression host. *J. Huazhong Agric. Univ.* **33,** 1–6 (2014).

2. Bai, X. P. et al. Heterologous biosynthesis of complex bacterial natural products in *Burkholderia gladioli*. *ACS Synth. Biol.* **12,** 3072–3081 (2023).

3. Chater, K. F. & Wilde, L. C. Restriction of a bacteriophage of *Streptomyces albus* G involving endonuclease *Sal*I. *J. Bacteriol.* **128,** 644–650 (1976).

4. Gomez-Escribano, J. P. & Bibb, M. J. Engineering for heterologous expression of secondary metabolite gene clusters. *Microb. Biotechnol.* **4,** 207–215 (2011).

5. Guo, W. L. et al. Identification and characterization of a strong constitutive promoter *stnYp* for activating biosynthetic genes and producing natural products in *Streptomyces*. *Microb. Cell Fact.* **22**, 127 (2023).

6. Jiang, C. et al. Establishing an efficient salinomycin biosynthetic pathway in three heterologous *Streptomyces* hosts by constructing a 106-kb multioperon artificial gene cluster. *Biotechnol. Bioeng.* **118**, 4668-4677 (2021).

7. Jiang, Y., Chen, B., Duan, C. L., Sun, B. B., Yang, J. J. & Yang, S. Multigene editing in the *Escherichia coli* genome via the CRISPR-Cas9 system. *Appl. Environ. Microbiol.* **81**, 2506-14 (2015).

8. Johnson, S. L. et al. Complete genome sequences for 59 *Burkholderia* isolates, both pathogenic and near neighbor. *Genome Announc.* **3**, e00159–15 (2015).

9. Huang, H., Zheng, G. S., Jiang, W. H., Hu, H. F. & Lu, Y. H. One-step high-efficiency CRISPR/Cas9-mediated genome editing in *Streptomyces*. *Acta. Bioch. Bioph. Sin.* **47,** 231–243 (2015).

10. Lee, N. C., Larionov, V. & Kouprina, N. Highly efficient CRISPR/Cas9-mediated TAR cloning of genes and chromosomal loci from complex genomes in yeast. *Nucleic Acids Res.* **43**, e55 (2015).

11. Li, L., Zheng, G. S., Chen, J., Ge, M., Jiang, W. H. & Lu Y. Multiplexed site-specific genome engineering for overproducing bioactive secondary metabolites in actinomycetes. *Metab. Eng.* **40**, 80–92 (2017).

12. Li, L. et al. aMSGE: advanced multiplex site-specific genome engineering with orthogonal modular recombinases in actinomycetes. *Metab. Eng.* **52**, 153–167 (2019).

13. Luo, M. & Wing, R. A. An improved method for plant BAC library construction. *Methods Mol. Biol.* **236**, 3–20 (2003)

14. Novakova, R. et al. Increased heterologous production of the antitumoral polyketide mithramycin A by

engineered *Streptomyces lividans* TK24 strains. *Appl. Microbiol. Biotechnol.* **102**, 857–869 (2018).

15. Wang, W. F. et al. Developing a robust genome editing tool based on an endogenous type I-B CRISPR-Cas system in *Saccharopolyspora spinosa*. *Sci. China Life Sci.* **68**, 1324–1336 (2025).

16. Xu, M. et al. Functional genome mining for metabolites encoded by large gene clusters through heterologous expression of a whole-genome bacterial artificial chromosome library in *Streptomyces spp*. *Appl. Environ. Microbiol.* **82**, 5795–5805 (2016).

17. Yamanaka, K. et al. Direct cloning and refactoring of a silent lipopeptide biosynthetic gene cluster yields the antibiotic taromycin A. *Proc. Natl. Acad. Sci. U. S. A.* **111,** 1957–1962 (2014).

18. Yang, Z. J. et al. MGCEP 1.0: A genetic-engineered marine-derived chassis cell for a scaled heterologous expression platform of microbial bioactive metabolites. *ACS Synth. Biol.* **11**, 3772–3784 (2022).

19. Zheng, G. S. et al. iCASRED, a scarless DNA editing tool in *E. coli* for high-efficiency engineering of natural product biosynthetic gene clusters. *Synth. Syst. Biotechnol.* **10**, 751–763 (2025).

20. Yarnall, M. T. N. et al. Drag-and-drop genome insertion of large sequences without double-strand DNA cleavage using CRISPR-directed integrases. *Nat. Biotechnol.* **41**, 500-512 (2021).
